# Vital@Work reintegration programme for sick-listed workers with stress-related complaints in occupational healthcare in the Netherlands: study protocol of a randomised controlled trial

**DOI:** 10.1136/bmjopen-2026-116576

**Published:** 2026-09-30

**Authors:** Eva E M van Doeveren, Allard W de Smalen, Roosmarijn M C Schelvis, Aartjan T F Beekman, Johannes R Anema, Trees T Juurlink

**Affiliations:** 1 Department Public and Occupational Health, Amsterdam UMC location Vrije Universiteit Amsterdam, Amsterdam, North Holland, Netherlands; 2 Societal Participation and Health, Amsterdam Public Health Research Institute, Amsterdam, North Holland, Netherlands; 3 Department Public and Occupational Health, Amsterdam UMC location University of Amsterdam, Amsterdam, North Holland, Netherlands; 4 Department of Psychiatry, Amsterdam University Medical Centre location VUmc, Amsterdam, North Holland, Netherlands

**Keywords:** MENTAL HEALTH, eHealth, Health Workforce, Occupational Health Services, Occupational Stress, Workplace

## Abstract

**Introduction:**

Mental health problems, including stress-related complaints, are an increasing concern in today’s workforce. Therefore, long-term sickness absence becomes a risk, yet effective return to work (RTW) interventions for employees suffering from mental health problems are scarce and they lack tailoring. The aim of this study is to describe the design of a randomised controlled trial (RCT) evaluating the effectiveness of the tailored Vital@Work reintegration programme for sick-listed workers with stress-related complaints.

**Methods and analysis:**

An RCT involving 208 sick-listed workers with stress-related complaints will be conducted with measurements taking place at baseline and after 3, 6 and 12 months. Stress-related complaints will be assessed with a distress screener. All participants will receive care as usual. The intervention group will receive a tailored eHealth programme, followed by the participatory approach if full RTW has not been achieved after 6 weeks in the study. Participants assigned to the control group will be provided with general information via the eHealth programme. The primary outcome is sustainable RTW, defined as lasting at least 28 consecutive days without any relapse. Secondary outcomes are stress-related complaints, psychosocial job characteristics and behaviour change. A mixed-methods approach will be used for the process evaluation.

**Ethics and dissemination:**

This study has been approved by the Medical Ethics Committee of Amsterdam UMC (approval number: 2024.0021). Results are expected to be published in peer-reviewed journals. In addition, interim and final results will be shared and discussed with the participating organisations involved in this study.

**Trial registration number:**

NCT06543732.

**STRENGTHS AND LIMITATIONS OF THIS STUDY**

- The use of standardised outcome measures, such as the Four-Dimensional Symptom Questionnaire, Patient Reported Outcomes Measurement Information System Questionnaire and Job Content Questionnaire, enhances the validity and reliability of our study findings.
- The mixed-methods approach provides insights into strengths and limitations for sustainable implementation of the intervention within different workplace contexts.
- The long-term follow-up of 1 year offers the opportunity to examine sustainable return to work.
- Only a single blinding of participants is possible due to the nature of the intervention.

## Introduction

In 2021, more than one billion people, representing about 14% of the global population, experienced poor mental health.^1^ This number is likely to increase in the coming years due to growing economic pressure, insufficient access to mental health services and poor psychosocial working conditions (eg, high workload and lack of support).^1–3^ Mental health problems have a profound impact on the working society. On an individual scale, they are associated with low productivity and long-term sickness absence.^4 5^ Mental health problems were even among the leading causes of long-term sickness absence in the Netherlands in 2023, constituting 40% of all absenteeism cases, of which 29% were attributable to stress-related complaints.^6^ From a societal perspective, the most prominent consequence of mental health complaints is the financial burden.^5 7^ For instance, anxiety and depressive complaints result in an estimated annual loss of 12 billion productive workdays across 36 countries, which together represent 80% of the global population.^1 8^ In the Netherlands, employers spent approximately €3.3 billion in 2021 on sickness absence costs caused by work stress alone.^5 7 9^ In the US the estimated costs amounted to US$187 billion.^5 9 10^ Moreover, prolonged absence from work is associated with a lower likelihood of eventual return to work (RTW) and a higher risk of depending on social benefits or choosing early retirement.^11 12^ This highlights the need for effective support throughout sickness absence.

Several interventions aimed at facilitating RTW of sick-listed workers have been developed and evaluated.^13–16^ The systematic review by Mikkelsen & Rosholm^13^ showed that interventions at the workplace are strong predictors for faster RTW of sick-listed workers with mental health complaints. Also, integrating multiple components, such as workplace contact combined with therapy, resulted in a faster RTW.^13^ Workplace involvement can thus lay the foundation for successful RTW at a later stage, as early contact initiatives are perceived as supportive by sick-listed workers.^17 18^ Additionally, the length of sickness absence is an important factor in determining the intervention’s effectiveness. In a review examining the effectiveness of interventions for individuals at risk or on long-term sickness absence, Tingulstad *et al*^14^ reported a considerable variation in sickness absence length across included studies, ranging from 1 to 24 months. The authors concluded that RTW rates were high within the first 8 weeks of sickness absence but gradually declined after 12 weeks, suggesting that early RTW efforts may be critical for successful reintegration.^14^ Notably, participants in this review could have been absent for any reason, and only two of the included studies specifically addressed workers with stress-related complaints. The interventions in these studies, workplace-based cognitive behavioural therapy (CBT) and stress management, showed a potentially positive effect on RTW, although the long-term impact remained relatively small.^16 19^ Furthermore, RTW interventions have shown effectiveness for workers with musculoskeletal disorders, but no evidence has yet been found for workers reporting (stress-related) mental health complaints.^20 21^

The present study will therefore combine effective elements of previous studies to support the RTW of sick-listed workers with stress-related complaints. It is grounded in two evidence-based interventions, which are subsequently explained: an eHealth programme and the participatory approach (PA). The eHealth programme is based on the Return@Work intervention by Volker *et al*^22^ and embodies the principles of CBT and problem-solving therapy.^22^ In this study, sick-listed participants in the eHealth programme group showed a faster time to first RTW than participants in the control group receiving only care as usual (CAU). No significant effects were found for time to full RTW.^22 23^ The PA, on the other hand, intervenes at the early onset of sickness absence and involves a structured process between the sick-listed worker and supervisor guided by an independent process leader to establish a consensus-based plan to facilitate RTW.^24^ A previous study evaluating the effectiveness of the PA for sick-listed workers with distress found no overall effect on RTW.^24^ However, the PA was effective in facilitating RTW among a subgroup of workers who, at baseline, reported a positive intention to resume work, despite the presence of stress-related complaints.^17 24^

Therefore, this paper describes the design of the Vital@Work reintegration programme. This programme combines the effective elements of the eHealth programme and the PA to improve sustainable RTW among sick-listed workers with stress-related complaints. Based on a triage assessment, the reintegration programme is tailored for each participant in the intervention group. It is hypothesised that participants receiving the tailored reintegration programme show a faster and more sustainable RTW as compared with the participants receiving the control condition. Next to the effectiveness evaluation, a process evaluation will be conducted to describe intervention adoption and understand barriers and facilitators to implementation across contexts.

## Methods and analysis

### Study design

This study is a two-arm randomised controlled trial (RCT) to evaluate the effectiveness of the Vital@Work reintegration programme for sick-listed workers with stress-related complaints. The study compares participants in the intervention group, who receive the reintegration programme, with a group receiving a control condition. All participants will receive CAU. Participants in both groups will be monitored for 1 year with measurements taking place at baseline and after 3, 6 and 12 months. In the last phase of the study period, a process evaluation will be conducted following the Reach, Effectiveness, Adoption, Implementation and Maintenance (RE-AIM) and Practical, Robust Implementation and Sustainability Model (PRISM) frameworks.^25–27^ In the process evaluation, the process and feasibility of the Vital@Work reintegration programme will be assessed to gain insight into the adoption and barriers and facilitators for implementation of the intervention. The Standard Protocol Items: Recommendations for Interventional Trials (SPIRIT) guidelines were followed while writing this protocol (online supplemental material 1).^28^

10.1136/bmjopen-2026-116576.supp1 Supplementary data

### Study population and setting

The study population consists of sick-listed workers across four participating organisations in the Netherlands. The organisations differ in size, sector (aviation, cleaning, hospital care and psychiatric healthcare), type (private or public), employee characteristics and target groups (eg, unskilled, practically trained or theoretically trained). These organisations represent a diverse group of sick-listed workers, allowing for research into which aspects of the intervention work for whom and under which conditions. Inclusion criteria for participants at enrolment of the study are: (1) on sickness absence between 2 and 12 weeks, (2) aged between 18 and 68 years, (3) having an employment contract for ≥12 hours per week and (4) suffering from stress-related complaints assessed by a distress screener. Exclusion criteria are: (1) suffering from severe psychiatric disorders (eg, suicidality, schizophrenia) or other chronic conditions (eg, terminal illness) that substantially interfere with RTW, (2) insufficient Dutch language skills to complete eHealth modules and/or questionnaires, (3) legal conflict with the employer and (4) pregnancy.

### Recruitment procedure

Workers who have been on sickness absence for 2–12 weeks will automatically receive an invitation email from their organisation’s Occupational Health Service (OHS). Every 1–2 weeks, such an email will be sent to newly sick-listed workers. The email contains brief information about the study, and the sick-listed workers can leave their contact details via an online link when interested to participate. Potential participants will then receive a screening questionnaire sent to their email address, including a distress screener, in which the inclusion and exclusion criteria are assessed. The distress screener comprises three questions derived from the Four-Dimensional Symptom Questionnaire (4DSQ), a validated instrument for identifying distress in sick-listed workers.^29^ The questions, which focus on stress-related complaints experienced during the past week, are rated on a scale from (0) ‘never’ to (4) ‘constantly’, and a worker meets the inclusion requirement if ≥3 points are scored. If the potential participant also fits the other inclusion criteria, a researcher will contact the potential participant by phone to provide further details about study participation. If the potential participant verbally agrees, an information letter and informed consent form will be sent by email (online supplemental material 2). Participation is official once the informed consent is digitally signed, after which the participant gains access to the eHealth programme and the baseline measurement questionnaires via a personal account.

10.1136/bmjopen-2026-116576.supp2 Supplementary data

### Intervention

Each participant, regardless of intervention or control group, will receive CAU from their organisation’s OHS. In the Dutch context, under the Dutch Gatekeeper Improvement Act, CAU consists of employees’ entitlement to occupational healthcare, whereby an occupational physician conducts a problem analysis and provides guidance based on the employee’s medical condition to facilitate (eventual) RTW. Participants assigned to the intervention group will, in addition to CAU by the occupational healthcare provider and eventual other care or treatment they receive for their condition, receive the Vital@Work reintegration programme, consisting of a tailored eHealth programme (see below under *eHealth programme*) and followed by the PA (see below under *Participatory approach*) if full RTW has not been achieved after 6 weeks. To ensure blinding, participants in the control group are provided with general information via the eHealth programme, which includes links to existing websites about health and RTW. All participants receive a personal Werk@Herstel account for the eHealth programme and questionnaires.

#### Process facilitators

The intervention group will be guided by a designated and trained process facilitator, who acts as a primary contact person and facilitator during the intervention period. The intervention period starts at enrolment of the study and will last until the PA is finished, typically after 3–4 months. The process facilitators are occupational health practitioners (eg, case managers, occupational nurses, company social workers) employed at the participating organisations. Prior to the start of the intervention, the process facilitator attends a day-long training organised by the researchers. The training includes a detailed explanation of the content of the eHealth programme, the PA components and role-play scenarios to practice the facilitators' role. Furthermore, the process facilitator adheres to a predefined protocol and remains neutral towards both the participant and supervisor. Process facilitators supporting participants in this study will not deliver CAU to these participants while administering the intervention.

#### eHealth programme

The eHealth programme is based on the original Return@Work intervention and adapted for the current study by incorporating the Dutch guidelines for employees with common mental disorders.^22 30^ Participants will follow either a psychological or a physical pathway, with or without extra psychoeducational, cognitive behavioural and/or problem-solving modules, depending on their scores on navigating questions at baseline. Figure 1 displays a simplified representation of the navigation of eHealth pathways. The eHealth pathways are determined by participants’ responses to two navigating questions and the 4DSQ in the baseline measurement. The 4DSQ is a self-report questionnaire designed to assess stress-related complaints across four dimensions: distress, depression, anxiety and somatisation.^29^ For each dimension, previously used cut-off scores are used to determine the tailored pathways.^29 31^ Depending on their responses, the participants may also receive psychoeducational, cognitive behavioural and/or problem-solving modules, ‘helping thoughts’ and/or ‘RTW with physical complaints’ from the other pathway. Pathways are individually tailored, as it is expected this will lead to a higher adherence within the intervention.

**Figure 1:**
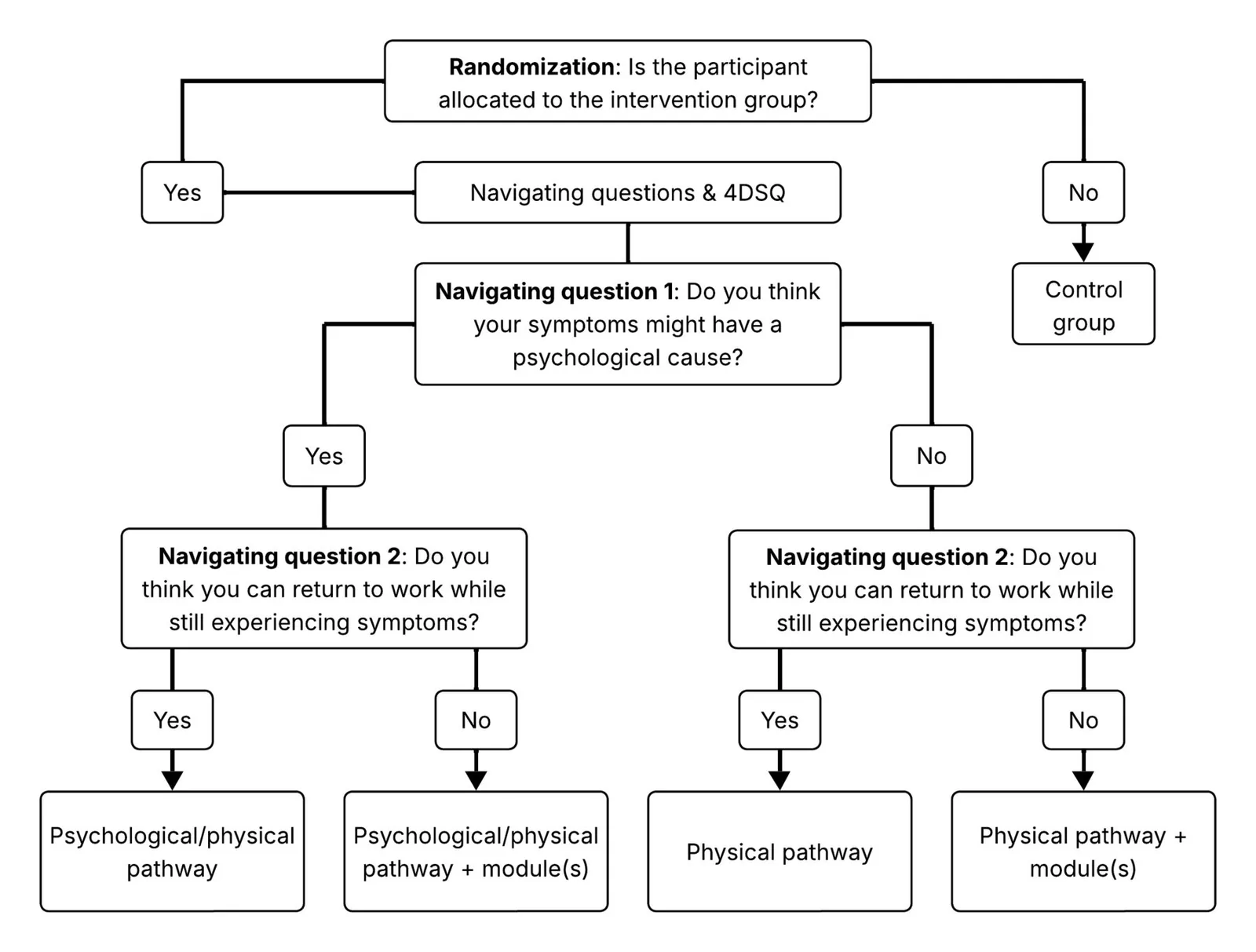
Decision aid of tailored eHealth pathways intervention group. Figure 1 displays a simplified representation of eHealth pathways. The pathways are determined by participants’ randomisation, followed by the two navigating questions and 4DSQ. Cut-off scores are used to determine the tailored pathways.^29 31^ The figure was created by EEMvD using Lucidchart. 4DSQ, Four-Dimensional Symptom Questionnaire.

The eHealth modules, based on the tailored pathways, potentially encompass the following courses:

- Psychoeducation on support for (work-related) psychological complaints.
- Problem-solving exercises for a bottleneck analysis by mapping work activities and related hindering factors.
- Problem-solving exercises using a stepwise approach to identify and cope with RTW barriers and other hindering factors at work.
- Information and exercises for early detection and/or maintaining good mental health.
- Cognitive behavioural exercises to identify (un)helpful thinking and reframing unhelpful thoughts, based on the CBT principles (*extra module*).
- Information about RTW with physical complaints and the impact of poor mental health on physical health (*extra module*).

Regardless of the tailored pathway, all participants receive eHealth modules focusing on psychoeducation, perceptions of RTW, including problem-solving exercises, relapse prevention and continuous monitoring. The modules serve as preparation for formulating an RTW plan, which is addressed in the PA that is offered if one is not fully returned to work after 6 weeks. Hence, participants can use their responses in the modules during the PA. For those fully resumed to work within 6 weeks, the eHealth programme and RTW plan can serve as a reflection and a useful reference in case of relapse. The participants progress through the eHealth programme independently, and each module must be completed before progressing to the next one. The assigned process facilitator will reach out 2 weeks after starting to check in on their progress and encourage module completion. Ideally, the eHealth programme is completed within 6 weeks after start. After 6 weeks, the participants are asked if they have returned to work or not. If participants have not yet fully returned to work, the PA is started regardless of the completion of the eHealth modules.

#### Participatory approach

After 6 weeks in the eHealth programme, all participants who did not return to work receive the PA. The PA is a stepwise process between the sick-listed worker (participant) and their supervisor aimed at identifying obstacles for RTW and subsequently developing a consensus-based RTW plan. The process facilitator will guide this process to enhance equal participation. Also, in the PA, tailoring is applied, as two variants of the PA are offered. That is: *individual work modifications* or *job coaching*, based on the participants’ perception of whether their complaints are work-related or not. This perception is questioned in the baseline screening questionnaire. If the participant perceives their stress-related complaints as work-related, the PA variant: *individual work modifications* will be offered. Conversely, if the issues are perceived to be unrelated to work, the participant will receive the PA variant: *job coaching*. Below, the six steps of PA are explained in detail:

- *Step 1 – Preparation:* The process facilitator will contact the participant and their supervisor to inform them about the PA and the upcoming steps. Also, meetings for the execution of the PA are planned. When the participant has already fully returned to work within 6 weeks, the PA is not started. The process facilitator will then inform the occupational physician (when permission is granted) and end their role as facilitator.
- *Step 2 – Barriers:* In this step, the most important barriers for returning to work are identified. First, individually between the process facilitator and the participant and second, between the process facilitator and the supervisor. This is done in two separate meetings in order to guide the participant and their supervisor in determining the most important and pressing task-related barriers to work. Further, the main responsibilities and activities of the participant are mapped out, indicating how often each task occurs and to what extent it impedes RTW.
- *Step 3 – Solutions:* In a three-way meeting (participant, supervisor and process facilitator), the participant and supervisor discuss the most important barriers for RTW. The aim is to reach consensus on which barriers should be addressed first for the RTW plan, guided by the process facilitator. Subsequently, both the participant and the supervisor prioritise solutions for the selected barriers.
- *Step 4 – RTW plan:* The selected barriers and solutions, as identified in steps 1–3, are documented in the RTW plan, including deadlines and responsibilities.
- *Step 5 – Implementation:* In this phase, the RTW plan is implemented within a time frame of 3 months.
- *Step 6 – Evaluation:* During the evaluation session, the RTW plan is evaluated. If necessary, the RTW plan will be adjusted.

The difference between the two PA variants lies in the presence of the process facilitator during the meetings. In the individual work modifications variant, the process facilitator will be physically present during all meetings with the participant and supervisor and guide the process. While for the job coaching variant, the process facilitator will remotely coach the process of the PA and can be consulted by the participant and/or supervisor when needed.

### Sample size

The sample size calculation is based on the primary outcome: time to sustainable RTW. Based on previous, similar research, time to sustainable RTW needs at least an HR of 2.0 to detect a clinical difference and to be relevant from a societal point of view.^24^ An HR of 2.0 indicates that participants in the intervention condition RTW two times as quickly as participants in the control condition. In the power analysis, an alpha of 0.05, a power of 80% and a potential dropout rate of 40% were considered. Hence, based on both the power analysis and comparable research, a sample size of 208 participants is needed for this study.^22–24^ A total of 208 participants is regarded as highly feasible, as the four participating organisations have a pool of more than 20 000 employees and a sickness absence rate of 2–5% each.

### Randomisation

Participants will be allocated to either the intervention or control group with a randomisation ratio of 1:1. Randomisation will be carried out via Castor EDC, an electronic system that manages data collection and security.^32^ The randomisation will be performed after the participants have signed the informed consent. Table 1 shows the timeline for enrolment, interventions and assessment procedure of the RCT. This is a single-blinded study, in which the participants are unaware of their allocation to the intervention or control group. Due to the need to assign participants to the PA intervention, it is not possible to blind research team members and the process facilitators. All parties involved will be instructed not to disclose the detailed research design to the participants.

**Table 1:** Participant timeline: schedule of enrolment, interventions and assessments

	Study period					
	Enrolment	Post-allocation				Close-out
Timepoint		*t_0_* *baseline*	*6 weeks*	*t_1_* *3 months*	*t_2_* *6 months*	*t_3_* *12 months*
Enrolment:						
Eligibility screening	x					
Informed consent	x					
Randomisation	x					
Interventions:						
eHealth modules		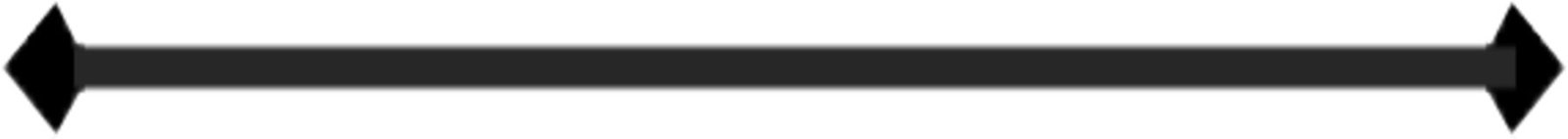				
Participatory approach			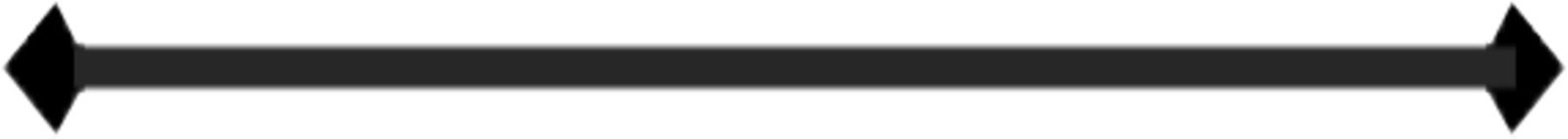			
Care as usual (all participants)		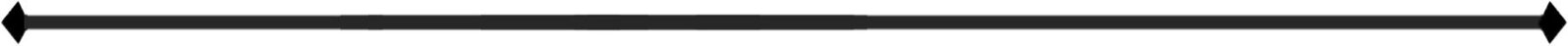				
Assessments:						
Participant characteristics		x				
RTW	x	x	x	x	x	x
Stress-related complaints	x	x		x	x	x
Self-reported health		x		x	x	x
Psychosocial job characteristics		x		x	x	x
Self-efficacy for RTW		x		x	x	x
Behavioural change for RTW		x				

CAU, care as usual; PA, participatory approach; RTW, return to work.

### Primary outcome

#### Return to work

The primary outcome is time to sustainable RTW, defined as a return to the employee’s previous or a similar position for at least 28 consecutive days without a partial or full relapse. RTW will be assessed through monthly self-reports via the online personal accounts in the eHealth programme Werk@Herstel. Participants will receive 12 monthly self-reports during the trial period. The report starts with the question, ‘Are you currently completely absent from work?’, and the participant indicates absence status with a ‘yes’ or ‘no’ and notes any work adjustments. Those not fully absent specify the percentage (%) of work resumed. Permission will be requested to obtain this data through sick leave register systems from the OHS of the employed organisation after 12 months (end of trial). Additionally, other work-related outcomes will be similarly measured after 12 months, such as RTW in any type of work and the number of sick days.

### Secondary outcomes

#### Stress-related complaints

Stress-related complaints will be measured using the 4DSQ self-report questionnaire across the four dimensions of distress, depression, anxiety and somatisation.^29^ The questionnaire consists of 50 items, measuring the severity of stress-related symptoms experienced in the past 7 days. The 4DSQ is scored on a 5-point Likert scale ranging from ‘no complaints’ to ‘very often or continuously’. The scores are converted to a 3-point scale with (0) ‘no complaints’ to (2) ‘regularly’ and upwards. The 4DSQ has demonstrated both validity and reliability in general practice, mental healthcare and research settings (Cronbach’s alpha of respective domains: 0.96, 0.96, 0.96 and 0.92).^33 34^ The 4DSQ will be disseminated at baseline and after 3, 6 and 12 months. The Patient Reported Outcomes Measurement Information System Questionnaire (PROMIS) will be used to measure the remaining self-reported health outcomes.^35^ The PROMIS in this study consists of a short version with 10 questions measuring physical, mental and social well-being across various domains. The questions about experienced well-being in the past 7 days are scored on a 5-point Likert scale ranging from (1) ‘poor well-being’ to (5) ‘excellent well-being’. In addition, a slider ranging from (0) 'no pain’ to (10) 'worst pain imaginable’ can be used to indicate pain experienced in the past week. In a previous study, the PROMIS short form showed excellent internal consistency (Cronbach’s alpha: >0.906).^36^ The PROMIS will be disseminated at baseline and after 3, 6 and 12 months.

#### Psychosocial job characteristics

Psychosocial job characteristics will be measured by the Job Content Questionnaire (JCQ), a validated instrument for measuring psychosocial job characteristics.^37^ Psychosocial job characteristics refer to the psychological and social aspects of work and the work environment. These include the mental workload, decision latitude, social support, physical demands and job insecurity. A 4-point Likert scale, ranging from (1) ‘strongly disagree’ to (4) ‘strongly agree’, will be used to assess levels of agreement to certain statements. An example of a statement is: 'I have many opportunities to participate in decisions about what happens in my work’. The degree of job insecurity in the past year will be asked in direct questions. The scale reliability of the JCQ is dependent on occupation but overall acceptable (Cronbach’s alpha: 0.61–0.87).^38^ The JCQ will be administered at baseline and after 3, 6 and 12 months.

#### Behaviour change

Behaviour change will be assessed by means of the attitude, social influence and self-efficacy (ASE) and return-to-work self-efficacy (RTW-SE).^22 39^ The ASE is a questionnaire that identifies the three factors influencing behaviour, specifically in the context of returning to work. For this questionnaire a 5-point Likert scale is used, ranging from (1) ‘strongly agree’ to (5) ‘strongly disagree’, for statements like *‘*Despite my complaints, I am going back to work because that contributes to my recovery*’*. The RTW-SE is a similar questionnaire but focuses on an individual’s self-efficacy regarding their confidence in successfully returning to work. The RTW-SE is scored on a 6-point Likert scale ranging from (1) ‘strongly disagree’ to (6) ‘strongly agree’. The RTW-SE has proven to be of excellent internal consistency in a similar setting (Cronbach’s alpha: 0.91).^40^ The ASE will be administered only at baseline, while the RTW-SE will be administered at baseline as well as after 3, 6 and 12 months.

### Covariates

The following covariates will be collected: (1) demographic data, (2) job characteristics and (3) psychosocial risk factors. Demographic data include age, gender, nationality, educational level and family situation. Job characteristics include type of work, type of employment contract (permanent or temporary) and working hours. Both demographic data and job characteristics will be administered at baseline. Psychological risk factors will be measured by the JCQ, which will be administered at baseline and after 3, 6 and 12 months.

### Process evaluation

The process and feasibility of the intervention will be assessed by means of a process evaluation to gain insight into the adoption as well as the barriers and facilitators to implementation of the intervention among the four organisations, based on the RE-AIM and PRISM frameworks.^25–27^ The aim of this process evaluation is to study the adoption and implementation of the intervention to gain insight into factors that improve or restrain sustainable implementation among sick-listed workers, supervisors and process facilitators. Therefore, the following process indicators will be studied: satisfaction, acceptability, reach, adoption, dose delivered and dose received and penetration and sustainability among different end-users (sick-listed worker, supervisor and process facilitator). Data will be gathered and analysed both quantitatively and qualitatively. Via logs, the process measures, reach, adoption and dose delivered and received will be gathered. To illustrate, the usage of the eHealth programme and attendance logs will be tracked. At approximately 9 months after commencement of the study, qualitatively the satisfaction, acceptability and barriers and facilitators to penetration and sustainability of the implementation of the intervention in individual semi-structured interviews with employees (n~10), a focus group interview or individual interview with supervisors (n~6) (depending on participant preference) and a focus group interview among process facilitators (n~6) as the implementers of the intervention will be conducted. The interviews will be audio-recorded and transcribed verbatim. Analyses will be performed in SPSS/R for the quantitative measures and MAXQDA software for the qualitative measures.

### Statistical analyses

Analyses will be conducted according to the intention-to-treat principle, meaning that participants will be analysed in the groups to which they were originally assigned. A descriptive analysis will be conducted for baseline characteristics and other variables, such as gender, age, educational level, occupation and sick leave duration. Longitudinal survival analyses will be conducted to compare the effect of the intervention group with the control group over time, including multiple subgroup analyses. The Cox proportional hazard model will be employed to compute HRs and standard errors will be appropriately adjusted for clustering if needed. Furthermore, confounding and/or effect modification of several factors (eg, gender, age, education) will be checked. The quantitative data will be analysed using SPSS/R, and the qualitative MAXQDA will be used.

### Patient and public involvement

Patients and the public were involved at several stages of this study. Prior to the start of the trial, focus groups with employees, supervisors and management of the participating organisations were conducted. During these focus groups, barriers and insights for implementation and intervention readiness were discussed to shape the current study design.^23^ The process evaluation of Volker *et al*^23^ also found that the occupational physicians, who acted as process facilitators, provided limited support and applied the intervention only to a limited extent.^22^ Therefore, in the current study, other occupational health professionals working at the participating organisation have been trained and deployed as dedicated process facilitators.

## Ethics and dissemination

The trial has been approved by the Medical Ethics Committee of Amsterdam UMC, which ruled that the Medical Research Involving Human Subjects Act (WMO) does not apply to this study (approval number: 2024.0021). All participants will be informed that participation is voluntary and that they can withdraw from the study at any time. Data collected prior to withdrawal will still be used. Participants will receive both written and verbal information and will have the opportunity to ask questions prior, during and after the study. Access to participant data is restricted to the researchers directly involved in the study. All participants receive an individual login code for the eHealth programme. Only the researcher team members and the web administrator can access all individual accounts. At the start of the study, each participant will be assigned a unique study code under which all collected data will be stored. Identification keys will be stored in a separate location and secured with a password. The trial is registered at ClinicalTrials.gov on 8 August 2024 (trial registration number: NCT06543732). Recruitment of the study started on 6 January 2025, and the trial is expected to be completed in December 2026.

Results are expected to be published in peer-reviewed journals and will be presented at national and international conferences. In addition, interim and final results will be shared with the participating organisations involved in this study. During committee meetings with these organisations, also the potential continuation of the intervention within their organisation will be discussed. Participants may receive updates or results of the study on request.

## Discussion

In this paper, the design of an RCT evaluating the effectiveness of the Vital@Work reintegration programme for sick-listed workers with stress-related complaints is described. Participants in the intervention group, who receive a tailored reintegration programme, will be compared with a control group. This intervention combines the effective components of an eHealth programme and the PA from previous research.^22 24^ By combining and using a tailored approach, we strive to fill the research gaps of effective RTW programmes for workers suffering from stress-related complaints. A key strength of this study lies in its tailored study design, which allows us to study different subgroups and contextual factors in participants with stress-related complaints. Another strength concerns the follow-up period in our study. Assessments take place at baseline after 3, 6 and 12 months, providing the opportunity to study participants’ RTW and stress-related complaints over the long term. This is relevant, as relapse within 1 year after RTW frequently occurs,^41 42^ and thus evaluating the intervention’s effectiveness in the long term is significant. A few limitations should also be considered. One of the limitations is that the researchers and the process facilitators cannot be blinded due to intervention allocation. However, all involved parties will be instructed not to disclose the research design to the participants, ensuring that the participants remain blinded as the control group is provided with general information via the eHealth programme. Another limitation is that the outcome measures are based on self-reported questionnaires (4DSQ, PROMIS, JCQ, ASE and RTW-SE). Participants may experience recall problems with the monthly reports, and a response bias may occur. Hence, to limit potential recall bias for the primary outcome (time to sustainable RTW), sick leave data from the register systems of the OHS of the employed organisation will be obtained (when permission is granted). By using both self-reported and register data, validity could be increased.^43^

## Supplementary Material

Reviewer comments

Author's manuscript
