# Supplementary material for: Vital@Work reintegration programme for sick-listed workers with stress-related complaints in occupational healthcare in the Netherlands: study protocol of a randomised controlled trial
Source: BMJ Open. 2026 Sep 30;16(9):e116576. doi: 10.1136/bmjopen-2026-116576 (PMC13636312; doi:10.1136/bmjopen-2026-116576)
Supplement: Supplementary file 2 [file bmjopen-16-9-s002.pdf]

Informed consent form (participant)

Vital@Work: a re-integration programme for employees with stress-related complaints

- I have read the information letter. I was also able to ask questions. My questions have been sufficiently answered. I had enough time to decide whether I want to participate.
- I understand that participation is voluntary. I also understand that I can withdraw from the study at any time. I do not have to provide a reason for this.
- I give permission for my data to be collected and used in the manner and for the purposes as described in the information letter.
- I give permission for my data to be stored within Amsterdam UMC for another 10 years after this study.
- I would like to participate in this study.

I give permission for the collection of my data:
O yes
O no

Date of permission (dd-mm-yyyy):

____

I give permission for requesting my (partial) recovery notifications from the absence database at my occupational health service:
O yes
O no

I give permission to inform my occupational physician about my participation in the study:
O yes
O no

I give permission to be contacted to take part in an interview:
O yes
O no

I give permission to be contacted again after this study for follow-up research:
O yes
O no

van Doeveren EEM, *et al. BMJ Open* 2026; 16:e116576. doi: 10.1136/bmjopen-2026-116576
