# Supplementary material for: Vital@Work reintegration programme for sick-listed workers with stress-related complaints in occupational healthcare in the Netherlands: study protocol of a randomised controlled trial
Source: BMJ Open. 2026 Sep 30;16(9):e116576. doi: 10.1136/bmjopen-2026-116576 (PMC13636312; doi:10.1136/bmjopen-2026-116576)
Supplement: Supplementary file 3 [file bmjopen-2026-116576.draft_revisions.pdf]

BMJ Open is committed to open peer review. As part of this commitment we make the peer review history of every article we publish publicly available.

When an article is published we post the peer reviewers' comments and the authors' responses online. We also post the versions of the paper that were used during peer review. These are the versions that the peer review comments apply to.

The versions of the paper that follow are the versions that were submitted during the peer review process. They are not the versions of record or the final published versions. They should not be cited or distributed as the published version of this manuscript.

BMJ Open is an open access journal and the full, final, typeset and author-corrected version of record of the manuscript is available on our site with no access controls, subscription charges or pay-per-view fees (http://bmjopen.bmj.com).

If you have any questions on BMJ Open's open peer review process please email info.bmjopen@bmj.com

BMJ Open

Vital@Work reintegration program for sick-listed workers with stress-related complaints: study protocol of a randomised controlled trial

Journal:	*BMJ Open*
Manuscript ID	bmjopen-2026-116576
Article Type:	Protocol
Date Submitted by the Author:	08-Jan-2026

Complete List of Authors:	van Doeveren, Eva; Amsterdam UMC Location VUmc, Public and Occupational Health; Amsterdam Public Health Research Institute, Societal Participation and Health<br>de Smalen, Allard; Amsterdam UMC Location VUmc, Public and Occupational Health; Amsterdam Public Health Research Institute, Societal Participation and Health<br>Schelvis, Roosmarijn; Amsterdam UMC Location AMC, Public and Occupational Health; Amsterdam Public Health Research Institute, Societal Participation and Health<br>Beekman, Aartjan ; Amsterdam UMC Locatie VUmc, Psychiatry; Amsterdam Public Health Research Institute, Societal Participation and Health<br>Anema, Johannes R.; Amsterdam UMC Location VUmc, Public and Occupational Health; Amsterdam Public Health Research Institute, Societal Participation and Health<br>Juurlink, Trees T; Amsterdam UMC Location VUmc, Public and Occupational Health ; Amsterdam Public Health Research Institute, Societal Participation and Health

Keywords:	MENTAL HEALTH, eHealth, Health Workforce, Occupational Health Services, Occupational Stress, Workplace

SCHOLARONE MANUSCRIPTS

STUDY PROTOCOL

Vital@Work reintegration program for sick-listed workers with stress-related complaints: study protocol of a randomised controlled trial

Eva E. M. van Doeveren[1,2], Allard W. de Smalen[1,2], Roosmarijn M. C. Schelvis[2,3], Aartjan T. F. Beekman[2,4], Johannes R. Anema[1,2] and Trees T. Juurlink[1,2]

Author affiliations:

[1]Amsterdam UMC location Vrije Universiteit Amsterdam, Department Public and Occupational Health, De Boelelaan 1117, Amsterdam, The Netherlands

[2]Amsterdam Public Health Research Institute, Societal Participation and Health, Amsterdam, The Netherlands

[3] Amsterdam UMC location University of Amsterdam, Department Public and Occupational Health, Meibergdreef 9, Amsterdam, The Netherlands,

[4]Amsterdam University Medical Centre location VUmc, Department of Psychiatry, De Boelelaan 1117, Amsterdam, The Netherlands

For peer review only

Abstract

Introduction: Mental health problems, including stress-related complaints, are an increasing concern in today's workforce. Therefore, long term sickness absence becomes a risk, yet effective return to work (RTW) interventions for employees suffering from mental health problems are scarce and they lack tailoring. The aim of this study is to describe the design of a randomised controlled trial (RCT) evaluating the effectiveness of the tailored Vital@Work reintegration program for sick-listed workers with stress-related complaints.

Methods and analysis: An RCT involving sick-listed workers with stress-related complaints will be conducted with measurements taking place at baseline, and after 3, 6 and 12 months. Stress-related complaints will be assessed with a distress screener. All participants will receive care as usual (CAU). The intervention group will receive a tailored eHealth program, followed by the participatory approach (PA) if full RTW has not been achieved after 6 weeks in the study. Participants assigned to the control group will be provided with general information via the eHealth program. The primary outcome is sustainable RTW, defined as lasting at least 28 consecutive days without any relapse. Secondary outcomes are stress-related complaints, self-reported health, psychosocial job characteristics, and self-efficacy. A mixed-methods approach will be used for the process evaluation.

Ethics and dissemination: This study has been approved by the Medical Ethics Committee of Amsterdam UMC (approval number: 2024.0021). Results are expected to be published in peer-reviewed

journals. In addition, interim and final results will be shared and discussed with the participating organisations involved in this study.

Trial registration number: NCT06543732.

Strengths and limitations of this study

- This randomised controlled trial addresses the critical issue of return to work in individuals with stress-related complaints as one of the main causes of today's absenteeism.
- By examining a tailored intervention within different contexts, this trial offers insights in effective return to work interventions for a diverse group of workers with stress-related complaints.
- The mixed-methods approach provides evidence and insights in strengths and limitations for sustainable implementation of the intervention within different workplace contexts.
- The long-term follow-up of one-year offers the opportunity to examine sustainable return to work.
- Only single blinding of participants is possible due to the nature of the intervention, however research members and process facilitators cannot be blinded because of allocating and providing the tailored approach.

Keywords: Mental health, Return to work, Workplace intervention, eHealth, Participatory Approach, Randomised controlled trial

Protocol version: 22 December 2025, version 1.0

Introduction

In 2021, more than one billion people, representing about 14% of the global population, experienced poor mental health [1]. This number is likely to increase in the coming years due to growing economic pressure, insufficient access to mental health services, and poor psychosocial working conditions (e.g. high workload and lack of support) [1-3]. Mental health problems have a profound impact on the working society. On an individual scale, they are associated with low productivity and long-term sickness absence [4, 5]. Mental health problems were even among the leading causes of long-term sickness absence in the Netherlands in 2023, constituting 40% of all absenteeism cases, of which 29% were attributable to stress-related complaints [6]. From a societal perspective, the most prominent consequence of mental health complaints is the financial burden. [5, 7]. For instance, anxiety and depressive complaints result in an estimated annual loss of 12 billion productive workdays across 36 countries, which together represent 80% of the global population [1, 8]. In the Netherlands, employers spent approximately €3.3 billion in 2021 on sickness absence costs caused by work stress alone [5, 7,

For peer review only - http://bmjopen.bmj.com/site/about/guidelines.xhtml

9]. In the United States the estimated costs amounted to US$187 billion [5, 9, 10]. Moreover, prolonged absence from work is associated with a lower likelihood of eventual return to work (RTW), and a higher risk of depending on social benefits or choosing early retirement [11, 12]. This highlights the need for effective support throughout sickness absence.

Several interventions aimed at facilitating RTW of sick-listed workers have been developed and evaluated [13-16]. The systematic review by Mikkelsen & Rosholm [13] showed that interventions at the workplace are strong predictors for faster RTW of sick-listed workers with mental health complaints. Also integrating multiple components, such as workplace contact combined with therapy, resulted in a faster RTW [13]. Workplace involvement can thus lay the foundation for successful RTW at a later stage, as early contact initiatives are perceived as supportive by sick-listed workers [17, 18]. Additionally, the length of sickness absence is an important factor in determining intervention's effectiveness. In a review examining the effectiveness of interventions for individuals at risk or on long-term sickness absence, Tingulstad et al. [14] reported a considerable variation in sickness absence length across included studies, ranging from 1 to 24 months. The authors concluded that RTW rates were high within the first 8 weeks of sickness absence but gradually declined after 12 weeks, suggesting that early RTW efforts may be critical for successful reintegration [14]. Notably, participants in this review could have been absent for any reason and only two of the included studies specifically addressed workers with stress-related complaints. The interventions in these studies, workplace-based cognitive behavioural therapy (CBT) and stress management, showed a potentially positive effect on RTW, although the long-term impact remained relatively small [16, 19]. Furthermore, RTW interventions have shown effectiveness for workers with musculoskeletal disorders, but no evidence has yet been found for workers reporting (stress-related) mental health complaints [20, 21].

The present study will therefore combine effective elements of previous studies to support the RTW of sick-listed workers with stress-related complaints. It is grounded in two evidence-based interventions, which are subsequently explained: an eHealth program and the Participatory Approach (PA). The eHealth program is based on the Return@Work intervention by Volker et al. [22] and embodies the principles of CBT and problem solving therapy (PST) [22]. In this study, sick-listed participants in the eHealth program group showed a faster time to first RTW than participants in the control group receiving only care as usual (CAU). No significant effects were found for time to full RTW [22, 23]. The PA on the other hand, intervenes at the early onset of sickness absence, and involves a structured process between the sick-listed worker and supervisor guided by an independent process leader to establish a consensus-based plan to facilitate RTW [24]. A previous study evaluating the effectiveness of the PA for sick-listed workers with distress found no overall effect on RTW [24]. However, the PA was effective in facilitating RTW among a subgroup of workers who, at baseline, reported a positive intention to resumption of work, despite the presence of stress-related complaints [17, 24].

For peer review only - http://bmjopen.bmj.com/site/about/guidelines.xhtml

Therefore, this paper describes the design of the Vital@Work reintegration program. This program combines the effective elements of the eHealth program and the PA to improve sustainable RTW among sick-listed workers with stress-related complaints. Based on a triage assessment, the reintegration program is tailored for each participant in the intervention group. It is hypothesised that participants receiving the tailored reintegration program show a faster and more sustainable return to work as compared to the participants receiving the control condition. Next to the effectiveness evaluation, a process evaluation will be conducted to describe intervention adoption and understand barriers and facilitators to implementation across contexts.

Methods and analysis

Study design

This study is a two-arm randomised controlled trial (RCT) to evaluate the effectiveness of the Vital@Work reintegration program for sick-listed workers with stress-related complaints. The study compares participants in the intervention group, who receive the reintegration program, with a group receiving a control condition. All participants will receive CAU. Participants in both groups will be monitored for one year with measurements taking place at baseline, and after 3, 6 and 12 months. In the last phase of the study period, a process evaluation will be conducted following the Reach, Effectiveness, Adoption, Implementation and Maintenance (RE-AIM), and Practical, Robust Implementation and Sustainability Model (PRISM) frameworks [25-27]. In the process evaluation, the process and feasibility of the Vital@Work reintegration program will be assessed to gain insight in the adoption, and barriers and facilitators for implementation of the intervention. The Standard Protocol Items: Recommendations for Interventional Trials (SPIRIT) guidelines were followed while writing this protocol (supplemental material 1) [28].

Study population and setting

The study population consists of sick-listed workers across four participating organisations in the Netherlands. The organisations differ in size, sector (aviation, cleaning, hospital care and psychiatric health care), type (private or public), employee characteristics and target groups (e.g. unskilled, practically trained or theoretically trained). These organisations represent a diverse group of sick-listed workers, allowing for research into which aspects of the intervention work for whom and under which conditions. Inclusion criteria for participants at enrolment of the study are: 1) on sickness absence between 2-12 weeks, 2) aged between 18-68 years, 3) having an employment contract for ≥12 hours per week, 4) suffering from stress-related complaints assessed by a distress screener. Exclusion criteria are: 1) suffering from severe psychiatric disorders (e.g. suicidality, schizophrenia) or other chronic conditions (e.g. terminal illness) that prevent RTW, 2) insufficient Dutch language skills to complete eHealth modules and/or questionnaires, 3) in legal conflict with the employer, 4) pregnancy.

For peer review only - http://bmjopen.bmj.com/site/about/guidelines.xhtml

Recruitment procedure

Workers who have been on sickness absence for 2-12 weeks will automatically receive an invitation email from their organisation's Occupational Health Service (OHS). Every 1-2 weeks, such an email will be sent to newly sick-listed workers. The email contains brief information about the study and the sick-listed workers can leave their contact details via an online link when interested to participate. Potential participants will then receive a screening questionnaire sent to their email address, including a distress screener, in which the inclusion and exclusion criteria are assessed. The distress screener comprises three questions derived from the Four-Dimensional Symptom Questionnaire (4DSQ), a validated instrument for identifying distress in sick-listed workers [29]. The questions, which focus on stress-related complaints experienced during the past week, are rated on a scale from (0) 'never' to (4) 'constantly' and a worker meets the inclusion requirement if ≥3 points are scored. If the potential participant also fits the other inclusion criteria, a researcher will contact the potential participant by phone to provide further details about study participation. If the potential participant verbally agrees, an information letter and informed consent form will be sent by email. Participation is official once the informed consent is digitally signed, after which the participant gains access to the eHealth program and the baseline measurement questionnaires via a personal account.

Intervention

Each participant, regardless of intervention or control group, will receive CAU from their organisation's OHS. Participants assigned to the intervention group will receive the Vital@Work reintegration program, consisting of a tailored eHealth program (see below under *eHealth program)* and followed by the PA (see below under *Participatory approach)* if full RTW has not been achieved after 6 weeks. To ensure blinding, participants in the control group are provided with general information via the eHealth program, which includes links to existing websites about health and RTW. All participants receive a personal Werk@Herstel account for the eHealth program and questionnaires.

Process facilitators

The intervention group will be guided by a designated and trained process facilitator, who acts as a primary contact person and facilitator during the intervention period. The intervention period starts at enrolment of the study and will last until the PA is finished, typically after 3-4 months. The process facilitators are occupational health practitioners (e.g. case manager, occupational nurse, company social worker) employed at the participating organisations. Prior to the start of the intervention, the process facilitator attends a day-long training organised by the researchers. The training includes a detailed explanation of the content of the eHealth program, the PA components, and role-play scenario's to practice the facilitators role. Furthermore, the process facilitator adheres to a predefined protocol, and

For peer review only - http://bmjopen.bmj.com/site/about/guidelines.xhtml

remains neutral towards both the participant and supervisor. Process facilitators supporting participants in this study will not deliver CAU to these participant while administering the intervention.

eHealth program

The eHealth program is based on the original Return@Work intervention, and adapted for the current study by incorporating the Dutch guidelines for employees with common mental disorders [22, 30]. Participants will follow either a psychological or a physical pathway, with or without extra psychoeducational, cognitive behavioural and/or problem solving modules, depending on their scores on navigating questions at baseline. Figure 1 displays a simplified representation of the navigation of eHealth pathways. The eHealth pathways are determined on participants' responses to two navigating questions and the 4DSQ in the baseline measurement. The 4DSQ is a self-report questionnaire designed to assess stress-related complaints across four dimensions: distress, depression, anxiety, and somatisation [29]. For each dimension, previously used cut-off scores are used to determine the tailored pathways [29, 31]. Depending on their responses, the participants may also receive psychoeducational, cognitive behavioural and/or problem solving modules 'helping thoughts' and/or 'RTW with physical complaints' from the other pathway. Pathways are individually tailored, as it is expected this will lead to a higher adherence within the intervention.

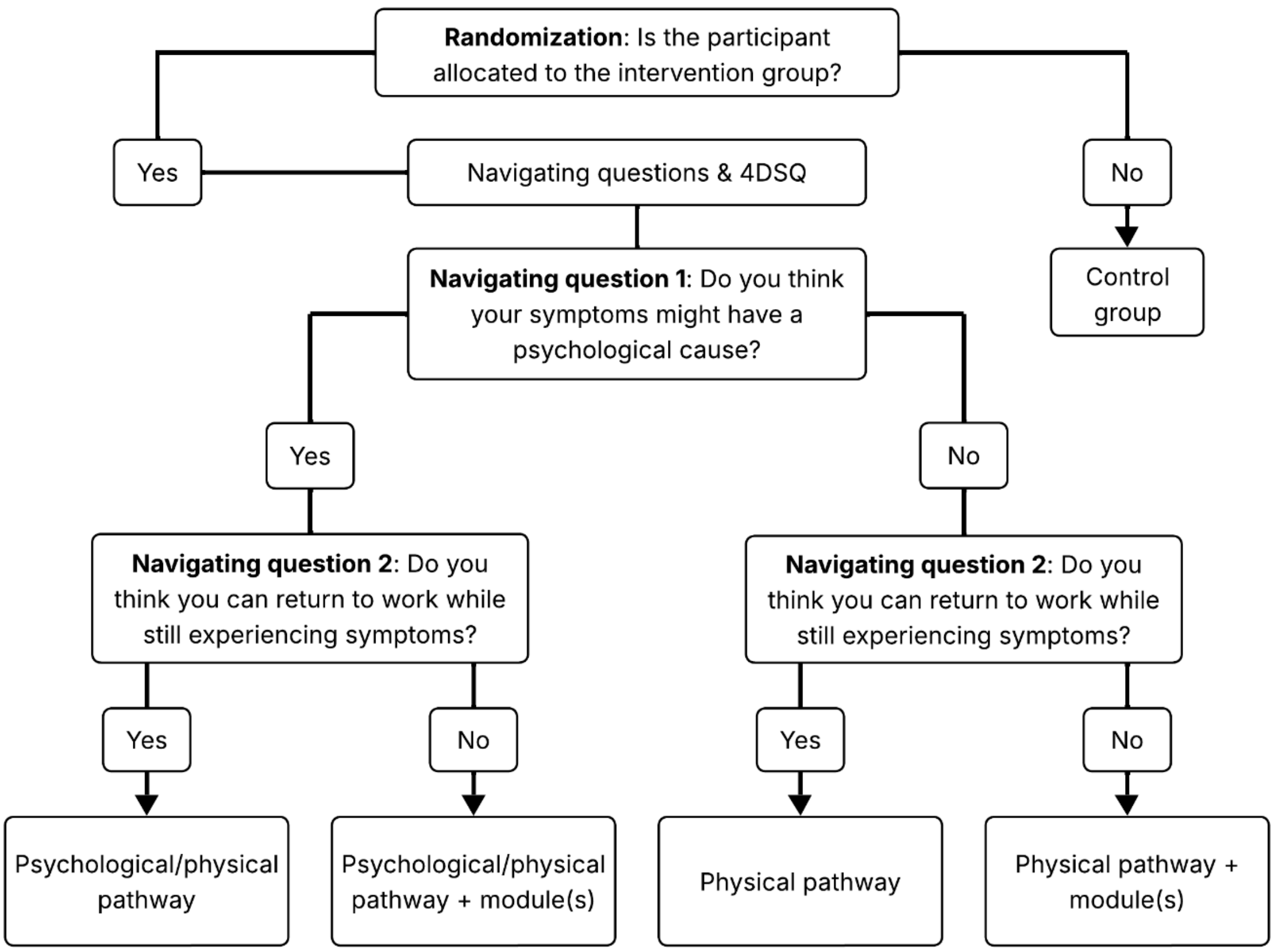

Fig. 1 Decision aid of tailored eHealth pathways intervention group

The eHealth modules, based on the tailored pathways, potentially encompass the following courses:

- Psychoeducation on support for (work-related) psychological complaints,
- Problem solving exercises for a bottleneck analysis by mapping work activities and related hindering factors,
- Problem solving exercises using a stepwise approach to identify and cope with RTW barriers and other hindering factors at work,
- Information and exercises for early detection and/or maintaining good mental health,
- Cognitive behavioural exercises to identify (un)helpful thinking and reframing unhelpful thoughts, based on the CBT principles *(extra module)*,
- Information about RTW with physical complaints and the impact of poor mental health on physical health *(extra module)*.

Regardless of tailored pathway, all participants receive eHealth modules focusing on psychoeducation, perceptions of RTW including problem-solving exercises, relapse prevention and continuous monitoring. The modules serve as preparation for formulating a RTW plan, which is addressed in the PA that is offered if one is not fully returned to work after 6 weeks. Hence, participants can use their responses in the modules during the PA. For those fully resumed to work within 6 weeks, the eHealth program and RTW plan can serve as reflection and useful reference in case of relapse. The participants progress through the eHealth program independently and each module must be completed before progressing to the next one. The assigned process facilitator will reach out 2 weeks after start to check in on their progress and encourage module completion. Ideally, the eHealth program is completed within 6 weeks after start. After 6 weeks, the participants are asked if they have returned to work or not. If participants have not yet fully returned to work, the PA is started regardless of completion of the eHealth modules.

Participatory approach

After 6 weeks in the eHealth program, all participants not returned to work receive the PA. The PA is a stepwise process between the sick-listed worker (participant) and their supervisor aimed at identifying obstacles for RTW, and subsequently developing a consensus-based RTW plan. The process facilitator will guide this process to enhance equal participation. Also in the PA tailoring is applied, as two variants of the PA are offered. That is: *individual work modifications* or *job coaching*, based on the participants' perception of whether their complaints are work-related or not. This perception is questioned in the baseline screening questionnaire. If the participant perceives their stress-related complaints as work-related, the PA variant: *individual work modifications* will be offered. Conversely, if the issues are

For peer review only - http://bmjopen.bmj.com/site/about/guidelines.xhtml

perceived to be unrelated to work, the participant will receive the PA variant: *job coaching.* Below, the six steps of PA explained in detail:

- *Step 1 – Preparation:* The process facilitator will contact the participant and their supervisor to inform about the PA and the upcoming steps. Also, meetings for execution of the PA are planned. When the participant has already fully returned to work within 6 weeks, the PA is not started. The process facilitator will then inform the occupational physician (when permission is granted) and ends their role as facilitator.
- *Step 2 – Barriers:* In this step, the most important barriers for returning to work are identified. First, individually between the process facilitator and the participant, and secondly between the process facilitator and the supervisor. This is done in two separate meetings in order to guide the participant and their supervisor in determining the most important and pressing task-related barriers to work. Further, the main responsibilities and activities of the participant are mapped out, indicating how often each task occurs and to what extend it impedes RTW.
- *Step 3 – Solutions:* In a three-way meeting (participant, supervisor and process facilitator) the participant and supervisor discuss the most important barriers for RTW. The aim is to reach consensus on which barriers should be addressed first for the RTW plan, guided by the process facilitator. Subsequently, both the participant and the supervisor prioritise solutions for the selected barriers.
- *Step 4 – RTW plan:* The selected barriers and solutions, as identified in steps 1-3, are documented in the RTW plan, including deadlines and responsibilities.
- *Step 5 – Implementation:* In this phase the RTW plan is implemented within a time frame of 3 months.
- *Step 6 – Evaluation:* During the evaluation session, the RTW plan is evaluated. If necessary, the RTW plan will be adjusted.

The difference of the two PA variants lies in the presence of the process facilitator during the meetings. In the individual work modifications variant, the process facilitator will be physically present during all meetings with the participant and supervisor and guides the process. While for the job coaching variant, the process facilitator will remotely coach the process of the PA, and can be consulted by the participant and/or supervisor when needed.

Sample size

The sample size calculation is based on the primary outcome: time to sustainable RTW. Based on previous, similar research, time to sustainable RTW needs at least a hazard ratio (HR) of 2.0 to detect a clinical difference and to be relevant from a societal point of view [24]. An HR of 2.0 indicates that

For peer review only - http://bmjopen.bmj.com/site/about/guidelines.xhtml

participants in the intervention condition RTW twice as quickly as participants in the control condition. In the power analysis, an alpha of 0.05, power of 80%, and a potential dropout rate of 40% was considered. Hence, based on both the power analysis and comparable research, a sample size of 208 participants is needed for this study [22-24]. A total of 208 participants is regarded as highly feasible, as the four participating organisations have a pool of more than 20,000 employees and a sickness absence rate of 2-5% each.

Randomisation

Participants will be allocated to either the intervention or control group with a randomisation ratio of 1:1. Randomisation will be carried out via Castor EDC, an electronic system that manages data collection and security [32]. The randomisation will be performed after the participants signed the informed consent. Table 1 shows the timeline for enrolment, interventions and assessment procedure of the RCT. This is a single-blinded study, in which the participants are unaware of their allocation to the intervention or control group. Due to the need to assign participants to the PA intervention, it is not possible to blind research team members and the process facilitators. All parties involved will be instructed not to disclose the detailed research design to the participants.

Table 1 Participant timeline: Schedule of enrolment, interventions, and assessments

	STUDY PERIOD					
	**Enrolment**	**Post-allocation**				**Close-out**
**TIMEPOINT**		***t_0* *baseline***	***6 weeks***	***t_1* *3 months***	***t_2* *6 months***	***t_3* *12 months***
**ENROLMENT:**						
**Eligibility screening**	x					
**Informed consent**	x					
**Randomisation**	x					
**INTERVENTIONS:**						
**eHealth modules**		◆———	———◆			
**Participatory approach**			◆———	———◆		
**Care as usual (all participants)**		◆———	————	————	————	———◆
**ASSESSMENTS:**						
**Participant characteristics**		x				

For peer review only - http://bmjopen.bmj.com/site/about/guidelines.xhtml

RTW	x	x	x	x	x	x
Stress-related complaints	x	x		x	x	x
Self-reported health		x		x	x	x
Psychosocial job characteristics		x		x	x	x
Self-efficacy for RTW		x		x	x	x
Behavioural change for RTW		x				

Primary outcome

Return to work

The primary outcome is time sustainable RTW, defined as a return to the employee's previous or a similar position for at least 28 consecutive days without a partial or full relapse. RTW will be assessed through monthly self-reports via the online personal accounts in the eHealth program Werk@Herstel. Participants will receive 12 monthly self-reports during the trial period. The report starts with the question, *'Are you currently completely absent from work?'*, and the participant indicates absence status with a 'yes' or 'no' and notes any work adjustments. Those not fully absent specify the percentage (%) of work resumed. Permission will be requested to obtain this data through sick leave register systems from the OHS of the employed organisation after 12 months (end of trial). Additionally, other work-related outcomes will be similarly measured after 12 months, such as RTW in any type of work and number of sick days.

Secondary outcomes

Stress-related complaints

Stress-related complaints will be measured using the 4DSQ self-report questionnaire across the four dimensions distress, depression, anxiety, and somatisation [29]. The questionnaire consists of 50 items, measuring the severity of stress-related symptoms experienced in the past seven days. The 4DSQ is scored on a 5-point Likert scale ranging from 'no complaints' to 'very often or continuously'. The scores are converted to a 3-point scale with (0) 'no complaints' to (2) 'regularly' and upwards. The 4DSQ has demonstrated both validity and reliability in general practice, mental health care, and research settings (Cronbach's alpha of respective domains: 0.96; 0.96; 0.96; and 0.92) [33, 34] . The 4DSQ will be disseminated at baseline, and after 3, 6 and 12 months.

Self-reported health

For peer review only - http://bmjopen.bmj.com/site/about/guidelines.xhtml

For peer review only

Self-reported health will be measured using the Patient Reported Outcomes Measurement Information System Questionnaire (PROMIS) [35]. The PROMIS in this study consists of a short version with 10 questions measuring physical, mental and social well-being across various domains. The questions about experienced well-being in the past seven days are scored on a 5-point Likert scale ranging from (1) 'poor well-being' to (5) 'excellent well-being'. In addition, a slider ranging from (0) 'no pain' to (10) 'worst pain imaginable' can be used to indicate pain experienced in the past week. In a previous study, the PROMIS short form showed excellent internal consistency (Cronbach's alpha: >0.906) [36]. The PROMIS will be disseminated at baseline, and after 3, 6 and 12 months.

Psychosocial job characteristics

Psychosocial job characteristics will be measured by the Job Content Questionnaire (JCQ), a validated instrument for measuring psychosocial job characteristics [37]. Psychosocial job characteristics refer to the psychological and social aspects of work and the work environment. These include the mental workload, decision latitude, social support, physical demands, and job insecurity. A 4-point Likert scale, ranging from (1) 'strongly disagree' to (4) 'strongly agree', will be used to assess levels of agreements to certain statements. An example of a statement is: *'I have many opportunities to participate in decisions about what happens in my work'*. The degree of job insecurity in the past year will be asked in direct questions. The scale reliability of the JCQ is dependent of occupation, but overall acceptable (Cronbach's alpha: 0.61-0.87) [38]. The JCQ will be administered at baseline, and after 3, 6 and 12 months.

Self-efficacy

Behaviour change will be assessed by means of the ASE (Attitude, Social influence and Self-Efficacy) and Return-to-Work Self-Efficacy (RTW-SE) [22, 39]. The ASE is a questionnaire that identifies the three factors influencing behaviour, specifically in the context of returning to work. For this questionnaire a 5-point Likert scale is used, ranging from (1) 'strongly agree' to (5) 'strongly disagree', for statements like *'Despite my complaints, I am going back to work, because that contributes to my recovery'*. The RTW-SE is a similar questionnaire, but focuses on an individual's self-efficacy regarding their confidence in successfully returning to work. The RTW-SE is scored on a 6-point Likert scale ranging from (1) 'strongly disagree' to (6) 'strongly agree'. The RTW-SE has proven to be of excellent internal consistency in a similar setting (Cronbach's alpha: 0.91) [40]. The ASE will be administered only at baseline, whilst the RTW-SE will be administered at baseline, as well after 3, 6 and 12 months.

Covariates

The following covariates will be collected: 1) demographic data, 2) job characteristics, 3) psychosocial risk-factors. Demographic data includes age, gender, nationality, educational level, and family situation.

For peer review only - http://bmjopen.bmj.com/site/about/guidelines.xhtml

Job characteristics include type of work, type of employment contract (permanent or temporary), and working hours. Both demographic data and job characteristics will be administered at baseline. Psychological risk-factors will be measured by the JCQ, which will be administered at baseline and after 3, 6, and 12 months.

Process evaluation

The process and feasibility of the intervention will be assessed by means of a process evaluation to gain insight in the adoption as well as the barriers and facilitators to implementation of the intervention among the four organisations, based on the RE-AIM and PRISM frameworks [25-27]. The aim of this process evaluation is to study the adoption and implementation of the intervention to gain insight into factors that improve or restrain sustainable implementation among sick-listed workers, supervisors, and process facilitators. Therefore, the following process indicators will be studied: satisfaction, acceptability, reach, adoption, dose delivered and dose received, and penetration and sustainability among different end-users (sick-listed worker, supervisor, and process facilitator). Data will be gathered and analysed both quantitatively and qualitatively. Via logs the process measures reach, adoption, and dose delivered and received will be gathered. To illustrate, the usage of the eHealth program and attendance logs will be tracked. At approximately 9 months after commencement of the study, qualitatively the satisfaction, acceptability and barriers and facilitators to penetration and sustainability of the implementation of the intervention in individual semi-structured interviews with employees (n~10), a focus group interview or individual interview with supervisors (n~6) (depending on participant preference), and a focus group interview among process facilitators (n~6) as the implementers of the intervention will be conducted. The interviews will be audio-recorded and transcribed verbatim. Analyses will be performed in SPSS/R for the quantitative measures and MAXQDA software for the qualitative measures.

Statistical analyses

Analyses will be conducted according to the intention-to-treat principle (ITT), meaning that participants will be analysed in the groups to which they were originally assigned. A descriptive analysis will be conducted for baseline characteristics and other variables, such as gender, age, educational level, occupation, and sick leave duration. Longitudinal survival analyses will be conducted to compare the effect of the intervention group with the control group over time, including multiple subgroup analyses. The Cox proportional hazard model will be employed to compute hazard ratios and standard errors will be appropriately adjusted for clustering if needed. Furthermore, confounding and/or effect modification of several factors (e.g., gender, age, education) will be checked. The quantitative data will be analysed using SPSS/R, regarding the qualitative MAXQDA will be used.

For peer review only - http://bmjopen.bmj.com/site/about/guidelines.xhtml

Patient and public involvement

Patients and the public were involved at several stages of this study. Prior to the start of the trial, focus groups with employees, supervisors, and management of the participating organisations were conducted. During these focus groups, barriers and insights for implementation and intervention readiness were discussed to shape the current study design [23]. Also, because the process evaluation of Volker et al. [23] found that the occupational physicians, who acted as process facilitators, provided limited support and applied the intervention only to a limited extent [22]. Therefore, in the current study, other occupational health professionals working at the participating organisation have been trained and deployed as dedicated process facilitator.

Ethics and dissemination

The trial has been approved by the Medical Ethics Committee of Amsterdam UMC, which ruled that the Medical Research Involving Human Subjects Act (WMO) does not apply to this study (approval number: 2024.0021). All participants will be informed that participation is voluntary and that they can withdraw from the study at any time. Data collected prior to withdrawal will still be used. Participants will receive both written and verbal information and will have the opportunity to ask questions prior, during and after the study. Access to participant data is restricted to the researchers directly involved in the study. All participants receive an individual login code for the eHealth program. Only the researcher team members and the web administrator can access all individual accounts. At the start of the study, each participant will be assigned a unique study code under which all collected data will be stored. Identification keys will be stored in a separate location and secured with a password. The trial is registered at ClinicalTrials.gov on August 8, 2024 (trial registration number: NCT06543732).

Results are expected to be published in peer-reviewed journals, and will be presented at national and international conferences. In addition, interim and final results will be shared with the participating organisations involved in this study. During committee meetings with these organisations, also the potential continuation of the intervention within their organisation will be discussed. Participants may receive updates or results of the study upon request.

Discussion

In this paper, the design of an RCT evaluating the effectiveness of the Vital@Work reintegration program for sick-listed workers with stress-related complaints, is described. Participants in the intervention group, who receive a tailored reintegration program, will be compared with a control group. This intervention combines the effective components of an eHealth program and the PA from previous research [22, 24]. By combining and using a tailored approach, we strive to fill the research gaps of effective RTW programs for workers suffering from stress-related complaints. A key strength of this

For peer review only - http://bmjopen.bmj.com/site/about/guidelines.xhtml

study lies in its tailored study design, which allows to study different subgroups and contextual factors in participants with stress-related complaints. Another strength concerns the follow-up period in our study. Assessments take place at baseline after 3, 6 and 12 months, providing the opportunity to study participants' RTW and stress-related complaints over the long-term. This is relevant as relapse within one year after RTW frequently occurs [41, 42], and thus evaluating the intervention's effectiveness in the long-term is significant. A few limitations should also be considered. One of the limitations is that the researchers and the process facilitators cannot be blinded due to intervention allocation. However, all involved parties will be instructed not to disclose the research design to the participants, ensuring that the participants remain blinded as the control group is provided with general information via the eHealth program. Another limitation is that the outcome measures are based on self-reported questionnaires (4DSQ, PROMIS, JCQ, ASE, and RTW-SE). Participants may experience recall problems with the monthly reports and a response bias may occur. Hence, to limit potential recall bias for the primary outcome (time to sustainable return to work), sick leave data from the register systems of the OHS of the employed organisation will be obtained (when permission is granted). By using both self-reported and register data, validity could be increased [43].

Authors' contributions

All authors will participate in the collection and execution of the trial; EEMvD and TTJ are primary executers. Authors AWdS, RMCS, ATFB, JRA and TTJ were responsible for the development of the trial intervention, with AWdS writing the non-WMO declaration. Authors RMCS, ATFB, and JRA obtained funding for this study. Author EEMvD wrote the first version of the paper; supervised by TTJ. Author TTJ provided content on the process evaluation. All authors have reviewed and approved the final manuscript.

Funding

This work was supported by Health~Holland, reference number LSHM22035-H025. The Vital@Work project is part of a PPP Allowance, made available by Health~Holland, Top Sector Life Sciences & Health, to stimulate public-private partnerships (https://www.health-holland.com). All involved organisations in the PPP (Amsterdam UMC, GGZ inGeest, Transavia, Rijksschoonmaakorganisatie) contributed in kind. Rijksschoonmaakorganisatie also contributed in cash.

Competing interests

JRA is consultant and certificate-holder of a spin-out company for implementation of the mobile application concerning the IkHerstel intervention in the Netherlands. JRA holds a chair in insurance medicine paid by the Dutch Social Security Agency; and has received grants from ZonMw, Nederlandse Organisatie voor Wetenschappelijk Onderzoek, Instituut Gak, Uitvoeringsinstituut Werknemersverzekeringen, Sociale Zaken en Werkgelegenheid, VWS (Volksgezondheid, Welzijn en

For peer review only - http://bmjopen.bmj.com/site/about/guidelines.xhtml

Sport), Pfizer, Achmea, CVZ (College voor Zorgverzekeringen), and Zorginstituut; all outside the submitted work. The other authors have no competing interests.

References

1. WHO. World mental health today: latest data. World Health Organization; 2025. Report No.: 9240113819.

2. WHO. World mental health report: Transforming mental health for all. World Health Organization; 2022. Report No.: 9240049339.

3. Niedhammer I, Bertrais S, Witt K. Psychosocial work exposures and health outcomes: a meta-review of 72 literature reviews with meta-analysis. Scandinavian journal of work, environment & health. 2021;47(7):489.

4. de Oliveira C, Saka M, Bone L, Jacobs R. The role of mental health on workplace productivity: a critical review of the literature. Applied health economics and health policy. 2023;21(2):167–93.

5. Wolvetang S, van Dongen JM, Speklé E, Coenen P, Schaafsma F. Sick leave due to stress, what are the costs for Dutch employers? Journal of Occupational Rehabilitation. 2022;32(4):764–72.

6. VZinfo. Ziekteverzuim | Naar oorzaak 2025 [Available from: https://www.vzinfo.nl/ziekteverzuim/naar-oorzaak.

7. Hassard J, Teoh KRH, Visockaite G, Dewe P, Cox T. The cost of work-related stress to society: A systematic review. Journal of occupational health psychology. 2018;23(1):1.

8. Chisholm D, Sweeny K, Sheehan P, Rasmussen B, Smit F, Cuijpers P, et al. Scaling-up treatment of depression and anxiety: a global return on investment analysis. The Lancet Psychiatry. 2016;3(5):415–24.

9. Van den Heuvel SG, Fernandez Beiro L, Van Dam LMC. Nationale enquête arbeidsomstandigheden 2021: Resultaten in vogelvlucht. TNO & CBS; 2022.

10. Goh J, Pfeffer J, Zenios SA. The relationship between workplace stressors and mortality and health costs in the United States. Management Science. 2016;62(2):608–28.

11. Vogel N, Schandelmaier S, Zumbrunn T, Ebrahim S, de Boer WEL, Busse JW, et al. Return-to-work coordination programmes for improving return to work in workers on sick leave. Cochrane Database of Systematic Reviews. 2017(3).

12. Prinz C. Sickness, disability and work: lessons from reforms and lack of change across the OECD countries. 2010.

13. Mikkelsen MB, Rosholm M. Systematic review and meta-analysis of interventions aimed at enhancing return to work for sick-listed workers with common mental disorders, stress-related disorders, somatoform disorders and personality disorders. Occupational and environmental medicine. 2018;75(9):675–86.

14. Tingulstad A, Meneses-Echavez J, Evensen LH, Bjerk M, Berg RC. Effectiveness of work-related interventions for return to work in people on sick leave: a systematic review and meta-analysis of randomized controlled trials. Systematic Reviews. 2022;11(1):192.

For peer review only

15. Blonk RWB, Brenninkmeijer V, Lagerveld SE, Houtman ILD. Rehabilitation in case of work-related psychological complaints: A field experiment among the self-employed. Work and Stress. 2006;20(2):129–44.

16. Dalgaard VL, Aschbacher K, Andersen JH, Glasscock DJ, Willert MV, Carstensen O, et al. Return to work after work-related stress: a randomized controlled trial of a work-focused cognitive behavioral intervention. Scandinavian Journal of Work, Environment & Health. 2017:436–46.

17. Hedlund Å, Kristofferzon M-L, Boman E, Nilsson A. Are return to work beliefs, psychological well-being and perceived health related to return-to-work intentions among women on long-term sick leave for common mental disorders? A cross-sectional study based on the theory of planned behaviour. BMC public health. 2021;21(1):535.

18. Buys NJ, Selander J, Sun J. Employee experience of workplace supervisor contact and support during long-term sickness absence. Disability and Rehabilitation. 2019;41(7):808–14.

19. Netterstrøm B, Friebel L, Ladegaard Y. Effects of a multidisciplinary stress treatment programme on patient return to work rate and symptom reduction: results from a randomised, wait-list controlled trial. Psychotherapy and psychosomatics. 2013;82(3):177–86.

20. Martin MHT, Nielsen MBD, Madsen IEH, Petersen SMA, Lange T, Rugulies R. Effectiveness of a coordinated and tailored return-to-work intervention for sickness absence beneficiaries with mental health problems. Journal of occupational rehabilitation. 2013;23(4):621–30.

21. Bültmann U, Sherson D, Olsen J, Hansen CL, Lund T, Kilsgaard J. Coordinated and tailored work rehabilitation: a randomized controlled trial with economic evaluation undertaken with workers on sick leave due to musculoskeletal disorders. Journal of occupational rehabilitation. 2009;19(1):81–93.

22. Volker D, Zijlstra-Vlasveld MC, Anema JR, Beekman ATF, Brouwers EPM, Emons WHM, et al. Effectiveness of a blended web-based intervention on return to work for sick-listed employees with common mental disorders: results of a cluster randomized controlled trial. Journal of medical Internet research. 2015;17(5):e116.

23. Volker D, Zijlstra-Vlasveld MC, Brouwers EPM, Van der Feltz-Cornelis CM. Process evaluation of a blended web-based intervention on return to work for sick-listed employees with common mental health problems in the occupational health setting. Journal of Occupational Rehabilitation. 2017;27(2):186–94.

24. van Oostrom SH, van Mechelen W, Terluin B, de Vet HCW, Knol DL, Anema JR. A workplace intervention for sick-listed employees with distress: results of a randomised controlled trial. Occupational and environmental medicine. 2010;67(9):596–602.

25. Gaglio B, Shoup JA, Glasgow RE. The RE-AIM framework: a systematic review of use over time. American journal of public health. 2013;103(6):e38–e46.

26. Feldstein AC, Glasgow RE. A practical, robust implementation and sustainability model (PRISM) for integrating research findings into practice. The joint commission journal on quality and patient safety. 2008;34(4):228–43.

27. Glasgow RE, Vogt TM, Boles SM. Evaluating the public health impact of health promotion interventions: the RE-AIM framework. American journal of public health. 1999;89(9):1322–7.

28. Chan A-W, Boutron I, Hopewell S, Moher D, Schulz KF, Collins GS, et al. SPIRIT 2025 statement: updated guideline for protocols of randomised trials. The Lancet. 2025;405(10491):e19–e27.

For peer review only - http://bmjopen.bmj.com/site/about/guidelines.xhtml

29. Terluin B, van Marwijk HWJ, Adèr HJ, de Vet HCW, Penninx BWJH, Hermens MLM, et al. The Four-Dimensional Symptom Questionnaire (4DSQ): a validation study of a multidimensional self-report questionnaire to assess distress, depression, anxiety and somatization. BMC psychiatry. 2006;6(1):34.

30. NVAB. Richtlijn Psychische problemen. 2019.

31. Terluin B, Oosterbaan DB, Brouwers EP, Van Straten A, Van de Ven PM, Langerak W, et al. To what extent does the anxiety scale of the Four-Dimensional Symptom Questionnaire (4DSQ) detect specific types of anxiety disorder in primary care? A psychometric study. BMC psychiatry. 2014;14(1):121.

32. CastorEDC. 2024 [Available from: https://www.castoredc.com/].

33. Kleinstäuber M, Exner A, Lambert MJ, Terluin B. Validation of the Four-Dimensional Symptom Questionnaire (4DSQ) in a mental health setting. Psychology, health & medicine. 2021;26(sup1):1–19.

34. Terluin B, Verbraak M. Validiteit van de Vierdimensionale Klachtenlijst (4DKL) in de ambulante geestelijke gezondheidszorg. Tijdschrift voor klinische psychologie. 2014;44(3):198–211.

35. Terwee CB, Roorda LD, De Vet HCW, Dekker J, Westhovens R, Van Leeuwen J, et al. Dutch–Flemish translation of 17 item banks from the patient-reported outcomes measurement information system (PROMIS). Quality of Life Research. 2014;23(6):1733–41.

36. Bartlett SJ, Orbai A-M, Duncan T, DeLeon E, Ruffing V, Clegg-Smith K, et al. Reliability and validity of selected PROMIS measures in people with rheumatoid arthritis. PloS one. 2015;10(9):e0138543.

37. Karasek R, Brisson C, Kawakami N, Houtman I, Bongers P, Amick B. The Job Content Questionnaire (JCQ): an instrument for internationally comparative assessments of psychosocial job characteristics. Journal of occupational health psychology. 1998;3(4):322.

38. Choi B, Kurowski A, Bond M, Baker D, Clays E, De Bacquer D, et al. Occupation-differential construct validity of the Job Content Questionnaire (JCQ) psychological job demands scale with physical job demands items: a mixed methods research. Ergonomics. 2012;55(4):425–39.

39. de Rijk A, Janssen N, van Lierop B, Alexanderson K, Nijhuis F. A behavioral approach to RTW after sickness absence: the development of instruments for the assessment of motivational determinants, motivation and key actors' attitudes. Work. 2009;33(3):273–85.

40. Gjengedal RGH, Lagerveld SE, Reme SE, Osnes K, Sandin K, Hjemdal O. The return-to-work self-efficacy questionnaire (RTW-SE): a validation study of predictive abilities and cut-off values for patients on sick leave due to anxiety or depression. Journal of occupational rehabilitation. 2021;31(3):664–73.

41. Koopmans PC, Bültmann U, Roelen CAM, Hoedeman R, van der Klink JJL, Groothoff JW. Recurrence of sickness absence due to common mental disorders. International archives of occupational and environmental health. 2011;84(2):193–201.

42. Spronken M, Brouwers EPM, Vermunt JK, Arends I, Oerlemans WGM, Van Der Klink JJL, et al. Identifying return to work trajectories among employees on sick leave due to mental health problems using latent class transition analysis. BMJ open. 2020;10(2):e032016.

43. Lavreysen O, Bakusic J, Abatzi T-A, Geerts A, Mateusen M, Bashkin O, et al. An overview of work-related stress assessment. Journal of affective disorders. 2025.

For peer review only - http://bmjopen.bmj.com/site/about/guidelines.xhtml

For peer review only

For peer review only - http://bmjopen.bmj.com/site/about/guidelines.xhtml

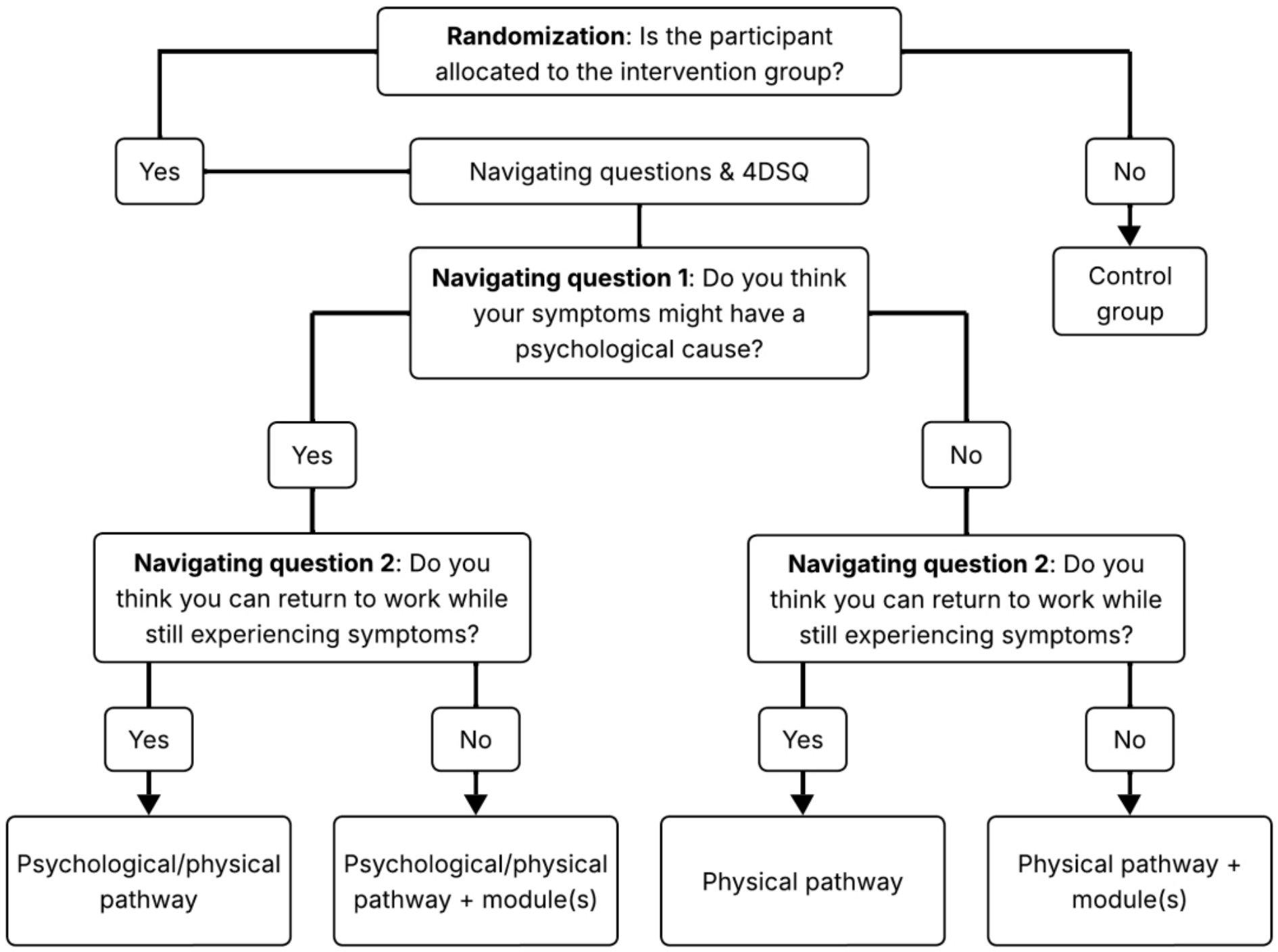

Fig. 1 Decision aid of tailored eHealth pathways intervention group

500x379mm (118 x 118 DPI)

SPIRIT 2025 checklist of items to address in a randomized trial protocol*

Section / Topic	No	SPIRIT 2025 checklist item description	Reported on page no.
**Administrative information**			
Title and structured summary	1a	Title stating the trial design, population, and interventions, with identification as a protocol	1
	1b	Structured summary of trial design and methods, including items from the World Health Organization Trial Registration Data Set	1-2
Protocol version	2	Version date and identifier	2
Roles and responsibilities	3a	Names, affiliations, and roles of protocol contributors	1 and 14
	3b	Name and contact information for the trial sponsor	14
	3c	Role of trial sponsor and funders in design, conduct, analysis, and reporting of trial; including any authority over these activities	14
	3d	Composition, roles, and responsibilities of the coordinating site, steering committee, endpoint adjudication committee, data management team, and other individuals or groups overseeing the trial, if applicable	14
**Open science**			
Trial registration	4	Name of trial registry, identifying number (with URL), and date of registration. If not yet registered, name of intended registry	2 and 13
Protocol and statistical analysis plan	5	Where the trial protocol and statistical analysis plan can be accessed	13
Data sharing	6	Where and how the individual de-identified participant data (including data dictionary), statistical code, and any other materials will be accessible	13
Funding and conflicts of interest	7a	Sources of funding and other support (e.g., supply of drugs)	14
	7b	Financial and other conflicts of interest for principal investigators and steering committee members	14
Dissemination policy	8	Plans to communicate trial results to participants, healthcare professionals, the public, and other relevant groups (e.g., reporting in trial registry, plain language summary, publication)	12-13
**Introduction**			
Background and rationale	9a	Scientific background and rationale, including summary of relevant studies (published and unpublished) examining benefits and harms for each intervention	2-4
	9b	Explanation for choice of comparator	2-4
Objectives	10	Specific objectives related to benefits and harms	12-13
**Methods: Patient and public involvement, trial design**			
Patient and public involvement	11	Details of, or plans for, patient or public involvement in the design, conduct, and reporting of the trial	12-13
Trial design	12	Description of trial design including type of trial (e.g., parallel group, crossover), allocation ratio, and framework (e.g., superiority, equivalence, non-inferiority, exploratory)	4

For peer review only - http://bmjopen.bmj.com/site/about/guidelines.xhtml

Methods: Participants, interventions, and outcomes			
Trial setting	13	Settings (e.g., community, hospital) and locations (e.g., countries, sites) where the trial will be conducted	4
Eligibility criteria	14a	Eligibility criteria for participants	4
	14b	If applicable, eligibility criteria for sites and for individuals who will deliver the interventions (e.g., surgeons, physiotherapists)	5-6
Intervention and comparator	15a	Intervention and comparator with sufficient details to allow replication including how, when, and by whom they will be administered. If relevant, where additional materials describing the intervention and comparator (e.g., intervention manual) can be accessed	5-8
	15b	Criteria for discontinuing or modifying allocated intervention/comparator for a trial participant (e.g., drug dose change in response to harms, participant request, or improving/worsening disease)	n/a
	15c	Strategies to improve adherence to intervention/comparator protocols, if applicable, and any procedures for monitoring adherence (e.g., drug tablet return, sessions attended)	5-6
	15d	Concomitant care that is permitted or prohibited during the trial	n/a
Outcomes	16	Primary and secondary outcomes, including the specific measurement variable (e.g., systolic blood pressure), analysis metric (e.g., change from baseline, final value, time to event), method of aggregation (e.g., median, proportion), and time point for each outcome	9-11
Harms	17	How harms are defined and will be assessed (e.g., systematically, non-systematically)	n/a
Participant timeline	18	Time schedule of enrollment, interventions (including any run-ins and washouts), assessments, and visits for participants. A schematic diagram is highly recommended (see Figure)	9-10 (table 1)
Sample size	19	How sample size was determined, including all assumptions supporting the sample size calculation	8-9
Recruitment	20	Strategies for achieving adequate participant enrollment to reach target sample size	5 and 8-9
**Methods: Assignment of interventions**			
Randomization:			
Sequence generation	21a	Who will generate the random allocation sequence and the method used	9
	21b	Type of randomization (simple or restricted) and details of any factors for stratification. To reduce predictability of a random sequence, other details of any planned restriction (e.g., blocking) should be provided in a separate document that is unavailable to those who enroll participants or assign interventions	9
Allocation concealment mechanism	22	Mechanism used to implement the random allocation sequence (e.g., central computer/telephone; sequentially numbered, opaque, sealed containers), describing any steps to conceal the sequence until interventions are assigned	9
Implementation	23	Whether the personnel who will enroll and those who will assign participants to the interventions will have access to the random allocation sequence	9
Blinding	24a	Who will be blinded after assignment to interventions (e.g., participants, care providers, outcome assessors, data analysts)	5 and 9

For peer review only - http://bmjopen.bmj.com/site/about/guidelines.xhtml

	24b	If blinded, how blinding will be achieved and description of the similarity of interventions	5 and 9
	24c	If blinded, circumstances under which unblinding is permissible, and procedure for revealing a participant's allocated intervention during the trial	n/a
**Methods: Data collection, management, and analysis**			
Data collection methods	25a	Plans for assessment and collection of trial data, including any related processes to promote data quality (e.g., duplicate measurements, training of assessors) and a description of trial instruments (e.g., questionnaires, laboratory tests) along with their reliability and validity, if known. Reference to where data collection forms can be accessed, if not in the protocol	10-11
	25b	Plans to promote participant retention and complete follow-up, including list of any outcome data to be collected for participants who discontinue or deviate from intervention protocols	n/a
Data management	26	Plans for data entry, coding, security, and storage, including any related processes to promote data quality (e.g., double data entry; range checks for data values). Reference to where details of data management procedures can be accessed, if not in the protocol	13
Statistical methods	27a	Statistical methods used to compare groups for primary and secondary outcomes, including harms	12
	27b	Definition of who will be included in each analysis (e.g., all randomized participants), and in which group	12
	27c	How missing data will be handled in the analysis	12
	27d	Methods for any additional analyses (e.g., subgroup and sensitivity analyses)	12
**Methods: Monitoring**			
Data monitoring committee	28a	Composition of data monitoring committee (DMC); summary of its role and reporting structure; statement of whether it is independent from the sponsor and funder; conflicts of interest and reference to where further details about its charter can be found, if not in the protocol. Alternatively, an explanation of why a DMC is not needed	14
	28b	Explanation of any interim analyses and stopping guidelines, including who will have access to these interim results and make the final decision to terminate the trial	13
Trial monitoring	29	Frequency and procedures for monitoring trial conduct. If there is no monitoring, give explanation	7
**Ethics**			
Research ethics approval	30	Plans for seeking research ethics committee/institutional review board approval	13
Protocol amendments	31	Plans for communicating important protocol modifications to relevant parties	n/a
Consent or assent	32a	Who will obtain informed consent or assent from potential trial participants or authorized proxies, and how	5
	32b	Additional consent provisions for collection and use of participant data and biological specimens in ancillary studies, if applicable	n/a
Confidentiality	33	How personal information about potential and enrolled participants will be collected, shared, and maintained in order to protect confidentiality before, during, and after the trial	13
Ancillary and post-trial care	34	Provisions, if any, for ancillary and post-trial care, and for compensation to those who suffer harm from trial participation	n/a

For peer review only - http://bmjopen.bmj.com/site/about/guidelines.xhtml

*We strongly recommend reading this checklist in conjunction with the SPIRIT 2025 Explanation and Elaboration and the SPIRIT 2025 Expanded Checklist for important clarifications on all the items. We also recommend reading relevant SPIRIT extensions. See www.consort-spirit.org

Citation: Chan A-W, Boutron I, Hopewell S, Moher D, Schulz KF, et al. SPIRIT 2025 statement: updated guideline for protocols of randomised trials. BMJ 2025;389:e081477. https://dx.doi.org/10.1136/bmj-2024-081477

© 2025 Chan A-W et al. This is an Open Access article distributed under the terms of the Creative Commons Attribution License (https://creativecommons.org/licenses/by/4.0/), which permits unrestricted use, distribution, and reproduction in any medium, provided the original work is properly cited.

For peer review only

For peer review only - http://bmjopen.bmj.com/site/about/guidelines.xhtml

BMJ Open

Vital@Work reintegration program for sick-listed workers with stress-related complaints in Occupational Healthcare in the Netherlands: study protocol of a randomised controlled trial

Journal:	*BMJ Open*
Manuscript ID	bmjopen-2026-116576.R1
Article Type:	Protocol
Date Submitted by the Author:	30-Jul-2026
Complete List of Authors:	van Doeveren, Eva; Amsterdam UMC location Vrije Universiteit Amsterdam, Department Public and Occupational Health; Amsterdam Public Health Research Institute, Societal Participation and Health<br>de Smalen, Allard; Amsterdam UMC location Vrije Universiteit Amsterdam, Department Public and Occupational Health; Amsterdam Public Health Research Institute, Societal Participation and Health<br>Schelvis, Roosmarijn; Amsterdam Public Health Research Institute, Societal Participation and Health, Amsterdam; Amsterdam UMC location University of Amsterdam, Department Public and Occupational Health<br>Beekman, Aartjan ; Amsterdam Public Health Research Institute, Societal Participation and Health, Amsterdam; Amsterdam University Medical Centre location VUmc, Department of Psychiatry<br>Anema, Johannes R.; Amsterdam UMC location Vrije Universiteit Amsterdam, Department Public and Occupational Health; Amsterdam Public Health Research Institute, Societal Participation and Health<br>Juurlink, Trees T; Amsterdam UMC location Vrije Universiteit Amsterdam, Department Public and Occupational Health ; Amsterdam Public Health Research Institute, Societal Participation and Health
**Primary Subject Heading**:	Occupational and environmental medicine
Secondary Subject Heading:	Mental health, Public health
Keywords:	MENTAL HEALTH, eHealth, Health Workforce, Occupational Health Services, Occupational Stress, Workplace

SCHOLARONE MANUSCRIPTS

STUDY PROTOCOL

Vital@Work reintegration program for sick-listed workers with stress-related complaints in Occupational Healthcare in the Netherlands: study protocol of a randomised controlled trial

Eva E. M. van Doeveren[1,2], Allard W. de Smalen[1,2], Roosmarijn M. C. Schelvis[2,3], Aartjan T. F. Beekman[2,4], Johannes R. Anema[1,2] and Trees T. Juurlink[1,2]

Author affiliations:

[1]Amsterdam UMC location Vrije Universiteit Amsterdam, Department Public and Occupational Health, De Boelelaan 1117, Amsterdam, The Netherlands

[2]Amsterdam Public Health Research Institute, Societal Participation and Health, Amsterdam, The Netherlands

[3] Amsterdam UMC location University of Amsterdam, Department Public and Occupational Health, Meibergdreef 9, Amsterdam, The Netherlands,

[4]Amsterdam University Medical Centre location VUmc, Department of Psychiatry, De Boelelaan 1117, Amsterdam, The Netherlands

For peer review only

Abstract

Introduction: Mental health problems, including stress-related complaints, are an increasing concern in today's workforce. Therefore, long term sickness absence becomes a risk, yet effective return to work (RTW) interventions for employees suffering from mental health problems are scarce and they lack tailoring. The aim of this study is to describe the design of a randomised controlled trial (RCT) evaluating the effectiveness of the tailored Vital@Work reintegration program for sick-listed workers with stress-related complaints.

Methods and analysis: An RCT involving 208 sick-listed workers with stress-related complaints will be conducted with measurements taking place at baseline, and after 3, 6 and 12 months. Stress-related complaints will be assessed with a distress screener. All participants will receive care as usual (CAU). The intervention group will receive a tailored eHealth program, followed by the participatory approach (PA) if full RTW has not been achieved after 6 weeks in the study. Participants assigned to the control group will be provided with general information via the eHealth program. The primary outcome is sustainable RTW, defined as lasting at least 28 consecutive days without any relapse. Secondary outcomes are stress-related complaints, self-reported health, psychosocial job characteristics, and self-efficacy. A mixed-methods approach will be used for the process evaluation.

Ethics and dissemination: This study has been approved by the Medical Ethics Committee of Amsterdam UMC (approval number: 2024.0021). Results are expected to be published in peer-reviewed journals. In addition, interim and final results will be shared and discussed with the participating organisations involved in this study.

Trial registration number: NCT06543732.

Strengths and limitations of this study

- This randomised controlled trial evaluates a return to work intervention for a diverse group of workers with stress-related complaints, using standardised outcome measures.
- The mixed-methods approach provides insights in strengths and limitations for sustainable implementation of the intervention within different workplace contexts.
- The long-term follow-up of one-year offers the opportunity to examine sustainable return to work.
- Only single blinding of participants is possible due to the nature of the intervention.

Keywords: Mental health, Return to work, Workplace intervention, eHealth, Participatory Approach, Randomised controlled trial

Protocol version: 29 July 2026, version 1.1

Introduction

In 2021, more than one billion people, representing about 14% of the global population, experienced poor mental health [1]. This number is likely to increase in the coming years due to growing economic pressure, insufficient access to mental health services, and poor psychosocial working conditions (e.g. high workload and lack of support) [1-3]. Mental health problems have a profound impact on the working society. On an individual scale, they are associated with low productivity and long-term sickness absence [4, 5]. Mental health problems were even among the leading causes of long-term sickness absence in the Netherlands in 2023, constituting 40% of all absenteeism cases, of which 29% were attributable to stress-related complaints [6]. From a societal perspective, the most prominent consequence of mental health complaints is the financial burden. [5, 7]. For instance, anxiety and depressive complaints result in an estimated annual loss of 12 billion productive workdays across 36 countries, which together represent 80% of the global population [1, 8]. In the Netherlands, employers spent approximately €3.3 billion in 2021 on sickness absence costs caused by work stress alone [5, 7, 9]. In the United States the estimated costs amounted to US$187 billion [5, 9, 10]. Moreover, prolonged absence from work is associated with a lower likelihood of eventual return to work (RTW), and a higher

For peer review only - http://bmjopen.bmj.com/site/about/guidelines.xhtml

risk of depending on social benefits or choosing early retirement [11, 12]. This highlights the need for effective support throughout sickness absence.

Several interventions aimed at facilitating RTW of sick-listed workers have been developed and evaluated [13-16]. The systematic review by Mikkelsen & Rosholm [13] showed that interventions at the workplace are strong predictors for faster RTW of sick-listed workers with mental health complaints. Also integrating multiple components, such as workplace contact combined with therapy, resulted in a faster RTW [13]. Workplace involvement can thus lay the foundation for successful RTW at a later stage, as early contact initiatives are perceived as supportive by sick-listed workers [17, 18]. Additionally, the length of sickness absence is an important factor in determining intervention's effectiveness. In a review examining the effectiveness of interventions for individuals at risk or on long-term sickness absence, Tingulstad et al. [14] reported a considerable variation in sickness absence length across included studies, ranging from 1 to 24 months. The authors concluded that RTW rates were high within the first 8 weeks of sickness absence but gradually declined after 12 weeks, suggesting that early RTW efforts may be critical for successful reintegration [14]. Notably, participants in this review could have been absent for any reason and only two of the included studies specifically addressed workers with stress-related complaints. The interventions in these studies, workplace-based cognitive behavioural therapy (CBT) and stress management, showed a potentially positive effect on RTW, although the long-term impact remained relatively small [16, 19]. Furthermore, RTW interventions have shown effectiveness for workers with musculoskeletal disorders, but no evidence has yet been found for workers reporting (stress-related) mental health complaints [20, 21].

The present study will therefore combine effective elements of previous studies to support the RTW of sick-listed workers with stress-related complaints. It is grounded in two evidence-based interventions, which are subsequently explained: an eHealth program and the Participatory Approach (PA). The eHealth program is based on the Return@Work intervention by Volker et al. [22] and embodies the principles of CBT and problem solving therapy (PST) [22]. In this study, sick-listed participants in the eHealth program group showed a faster time to first RTW than participants in the control group receiving only care as usual (CAU). No significant effects were found for time to full RTW [22, 23]. The PA on the other hand, intervenes at the early onset of sickness absence, and involves a structured process between the sick-listed worker and supervisor guided by an independent process leader to establish a consensus-based plan to facilitate RTW [24]. A previous study evaluating the effectiveness of the PA for sick-listed workers with distress found no overall effect on RTW [24]. However, the PA was effective in facilitating RTW among a subgroup of workers who, at baseline, reported a positive intention to resumption of work, despite the presence of stress-related complaints [17, 24].

Therefore, this paper describes the design of the Vital@Work reintegration program. This program combines the effective elements of the eHealth program and the PA to improve sustainable RTW among sick-listed workers with stress-related complaints. Based on a triage assessment, the

For peer review only - http://bmjopen.bmj.com/site/about/guidelines.xhtml

reintegration program is tailored for each participant in the intervention group. It is hypothesised that participants receiving the tailored reintegration program show a faster and more sustainable return to work as compared to the participants receiving the control condition. Next to the effectiveness evaluation, a process evaluation will be conducted to describe intervention adoption and understand barriers and facilitators to implementation across contexts.

Methods and analysis

Study design

This study is a two-arm randomised controlled trial (RCT) to evaluate the effectiveness of the Vital@Work reintegration program for sick-listed workers with stress-related complaints. The study compares participants in the intervention group, who receive the reintegration program, with a group receiving a control condition. All participants will receive CAU. Participants in both groups will be monitored for one year with measurements taking place at baseline, and after 3, 6 and 12 months. In the last phase of the study period, a process evaluation will be conducted following the Reach, Effectiveness, Adoption, Implementation and Maintenance (RE-AIM), and Practical, Robust Implementation and Sustainability Model (PRISM) frameworks [25-27]. In the process evaluation, the process and feasibility of the Vital@Work reintegration program will be assessed to gain insight in the adoption, and barriers and facilitators for implementation of the intervention. The Standard Protocol Items: Recommendations for Interventional Trials (SPIRIT) guidelines were followed while writing this protocol (supplemental material 1) [28].

Study population and setting

The study population consists of sick-listed workers across four participating organisations in the Netherlands. The organisations differ in size, sector (aviation, cleaning, hospital care and psychiatric healthcare), type (private or public), employee characteristics and target groups (e.g. unskilled, practically trained or theoretically trained). These organisations represent a diverse group of sick-listed workers, allowing for research into which aspects of the intervention work for whom and under which conditions. Inclusion criteria for participants at enrolment of the study are: 1) on sickness absence between 2-12 weeks, 2) aged between 18-68 years, 3) having an employment contract for ≥12 hours per week, 4) suffering from stress-related complaints assessed by a distress screener. Exclusion criteria are: 1) suffering from severe psychiatric disorders (e.g. suicidality, schizophrenia) or other chronic conditions (e.g. terminal illness) that substantially intervenes with RTW, 2) insufficient Dutch language skills to complete eHealth modules and/or questionnaires, 3) in legal conflict with the employer, 4) pregnancy.

For peer review only - http://bmjopen.bmj.com/site/about/guidelines.xhtml

Recruitment procedure

Workers who have been on sickness absence for 2-12 weeks will automatically receive an invitation email from their organisation's Occupational Health Service (OHS). Every 1-2 weeks, such an email will be sent to newly sick-listed workers. The email contains brief information about the study and the sick-listed workers can leave their contact details via an online link when interested to participate. Potential participants will then receive a screening questionnaire sent to their email address, including a distress screener, in which the inclusion and exclusion criteria are assessed. The distress screener comprises three questions derived from the Four-Dimensional Symptom Questionnaire (4DSQ), a validated instrument for identifying distress in sick-listed workers [29]. The questions, which focus on stress-related complaints experienced during the past week, are rated on a scale from (0) 'never' to (4) 'constantly' and a worker meets the inclusion requirement if ≥3 points are scored. If the potential participant also fits the other inclusion criteria, a researcher will contact the potential participant by phone to provide further details about study participation. If the potential participant verbally agrees, an information letter and informed consent form will be sent by email (supplemental material 2). Participation is official once the informed consent is digitally signed, after which the participant gains access to the eHealth program and the baseline measurement questionnaires via a personal account.

Intervention

Each participant, regardless of intervention or control group, will receive CAU from their organisation's OHS. In the Dutch context, under the Dutch Gatekeeper Improvement Act, CAU consists of employees' entitlement to occupational healthcare, whereby an occupational physician conducts a problem analysis and provides guidance based on the employee's medical condition, to facilitate (eventual) return to work. Participants assigned to the intervention group will, in addition to CAU by the occupational healthcare provider and eventual other care or treatment they receive for their condition, receive the Vital@Work reintegration program, consisting of a tailored eHealth program (see below under *eHealth program)* and followed by the PA (see below under *Participatory approach)* if full RTW has not been achieved after 6 weeks. To ensure blinding, participants in the control group are provided with general information via the eHealth program, which includes links to existing websites about health and RTW. All participants receive a personal Werk@Herstel account for the eHealth program and questionnaires.

Process facilitators

The intervention group will be guided by a designated and trained process facilitator, who acts as a primary contact person and facilitator during the intervention period. The intervention period starts at enrolment of the study and will last until the PA is finished, typically after 3-4 months. The process facilitators are occupational health practitioners (e.g. case manager, occupational nurse, company social worker) employed at the participating organisations. Prior to the start of the intervention, the process

For peer review only - http://bmjopen.bmj.com/site/about/guidelines.xhtml

facilitator attends a day-long training organised by the researchers. The training includes a detailed explanation of the content of the eHealth program, the PA components, and role-play scenario's to practice the facilitators role. Furthermore, the process facilitator adheres to a predefined protocol, and remains neutral towards both the participant and supervisor. Process facilitators supporting participants in this study will not deliver CAU to these participant while administering the intervention.

eHealth program

The eHealth program is based on the original Return@Work intervention, and adapted for the current study by incorporating the Dutch guidelines for employees with common mental disorders [22, 30]. Participants will follow either a psychological or a physical pathway, with or without extra psychoeducational, cognitive behavioural and/or problem solving modules, depending on their scores on navigating questions at baseline. Figure 1 displays a simplified representation of the navigation of eHealth pathways. The eHealth pathways are determined on participants' responses to two navigating questions and the 4DSQ in the baseline measurement. The 4DSQ is a self-report questionnaire designed to assess stress-related complaints across four dimensions: distress, depression, anxiety, and somatisation [29]. For each dimension, previously used cut-off scores are used to determine the tailored pathways [29, 31]. Depending on their responses, the participants may also receive psychoeducational, cognitive behavioural and/or problem solving modules 'helping thoughts' and/or 'RTW with physical complaints' from the other pathway. Pathways are individually tailored, as it is expected this will lead to a higher adherence within the intervention.

The eHealth modules, based on the tailored pathways, potentially encompass the following courses:

- Psychoeducation on support for (work-related) psychological complaints,
- Problem solving exercises for a bottleneck analysis by mapping work activities and related hindering factors,
- Problem solving exercises using a stepwise approach to identify and cope with RTW barriers and other hindering factors at work,
- Information and exercises for early detection and/or maintaining good mental health,
- Cognitive behavioural exercises to identify (un)helpful thinking and reframing unhelpful thoughts, based on the CBT principles *(extra module)*,
- Information about RTW with physical complaints and the impact of poor mental health on physical health *(extra module)*.

Regardless of tailored pathway, all participants receive eHealth modules focusing on psychoeducation, perceptions of RTW including problem-solving exercises, relapse prevention and continuous monitoring. The modules serve as preparation for formulating a RTW plan, which is addressed in the

For peer review only - http://bmjopen.bmj.com/site/about/guidelines.xhtml

PA that is offered if one is not fully returned to work after 6 weeks. Hence, participants can use their responses in the modules during the PA. For those fully resumed to work within 6 weeks, the eHealth program and RTW plan can serve as reflection and useful reference in case of relapse. The participants progress through the eHealth program independently and each module must be completed before progressing to the next one. The assigned process facilitator will reach out 2 weeks after start to check in on their progress and encourage module completion. Ideally, the eHealth program is completed within 6 weeks after start. After 6 weeks, the participants are asked if they have returned to work or not. If participants have not yet fully returned to work, the PA is started regardless of completion of the eHealth modules.

Participatory approach

After 6 weeks in the eHealth program, all participants not returned to work receive the PA. The PA is a stepwise process between the sick-listed worker (participant) and their supervisor aimed at identifying obstacles for RTW, and subsequently developing a consensus-based RTW plan. The process facilitator will guide this process to enhance equal participation. Also in the PA tailoring is applied, as two variants of the PA are offered. That is: *individual work modifications* or *job coaching*, based on the participants' perception of whether their complaints are work-related or not. This perception is questioned in the baseline screening questionnaire. If the participant perceives their stress-related complaints as work-related, the PA variant: *individual work modifications* will be offered. Conversely, if the issues are perceived to be unrelated to work, the participant will receive the PA variant: *job coaching.* Below, the six steps of PA explained in detail:

- *Step 1 – Preparation:* The process facilitator will contact the participant and their supervisor to inform about the PA and the upcoming steps. Also, meetings for execution of the PA are planned. When the participant has already fully returned to work within 6 weeks, the PA is not started. The process facilitator will then inform the occupational physician (when permission is granted) and ends their role as facilitator.
- *Step 2 – Barriers:* In this step, the most important barriers for returning to work are identified. First, individually between the process facilitator and the participant, and secondly between the process facilitator and the supervisor. This is done in two separate meetings in order to guide the participant and their supervisor in determining the most important and pressing task-related barriers to work. Further, the main responsibilities and activities of the participant are mapped out, indicating how often each task occurs and to what extend it impedes RTW.
- *Step 3 – Solutions:* In a three-way meeting (participant, supervisor and process facilitator) the participant and supervisor discuss the most important barriers for RTW. The aim is to reach consensus on which barriers should be addressed first for the RTW plan, guided by the process

For peer review only - http://bmjopen.bmj.com/site/about/guidelines.xhtml

facilitator. Subsequently, both the participant and the supervisor prioritise solutions for the selected barriers.

- *Step 4 – RTW plan:* The selected barriers and solutions, as identified in steps 1-3, are documented in the RTW plan, including deadlines and responsibilities.
- *Step 5 – Implementation:* In this phase the RTW plan is implemented within a time frame of 3 months.
- *Step 6 – Evaluation:* During the evaluation session, the RTW plan is evaluated. If necessary, the RTW plan will be adjusted.

The difference of the two PA variants lies in the presence of the process facilitator during the meetings. In the individual work modifications variant, the process facilitator will be physically present during all meetings with the participant and supervisor and guides the process. While for the job coaching variant, the process facilitator will remotely coach the process of the PA, and can be consulted by the participant and/or supervisor when needed.

Sample size

The sample size calculation is based on the primary outcome: time to sustainable RTW. Based on previous, similar research, time to sustainable RTW needs at least a hazard ratio (HR) of 2.0 to detect a clinical difference and to be relevant from a societal point of view [24]. An HR of 2.0 indicates that participants in the intervention condition RTW twice as quickly as participants in the control condition. In the power analysis, an alpha of 0.05, power of 80%, and a potential dropout rate of 40% was considered. Hence, based on both the power analysis and comparable research, a sample size of 208 participants is needed for this study [22-24]. A total of 208 participants is regarded as highly feasible, as the four participating organisations have a pool of more than 20,000 employees and a sickness absence rate of 2-5% each.

Randomisation

Participants will be allocated to either the intervention or control group with a randomisation ratio of 1:1. Randomisation will be carried out via Castor EDC, an electronic system that manages data collection and security [32]. The randomisation will be performed after the participants signed the informed consent. Table 1 shows the timeline for enrolment, interventions and assessment procedure of the RCT. This is a single-blinded study, in which the participants are unaware of their allocation to the intervention or control group. Due to the need to assign participants to the PA intervention, it is not possible to blind research team members and the process facilitators. All parties involved will be instructed not to disclose the detailed research design to the participants.

For peer review only - http://bmjopen.bmj.com/site/about/guidelines.xhtml

Table 1 Participant timeline: Schedule of enrolment, interventions, and assessments

	STUDY PERIOD					
	Enrolment	Post-allocation				Close-out
**TIMEPOINT**		*t_0 baseline*	*6 weeks*	*t_1 3 months*	*t_2 6 months*	*t_3 12 months*
**ENROLMENT:**						
**Eligibility screening**	x					
**Informed consent**	x					
**Randomisation**	x					
**INTERVENTIONS:**						
**eHealth modules**		◆———	———◆			
**Participatory approach**			◆———	———◆		
**Care as usual (all participants)**		◆———	———	———	———	———◆
**ASSESSMENTS:**						
**Participant characteristics**		x				
**RTW**	x	x	x	x	x	x
**Stress-related complaints**	x	x		x	x	x
**Self-reported health**		x		x	x	x
**Psychosocial job characteristics**		x		x	x	x
**Self-efficacy for RTW**		x		x	x	x
**Behavioural change for RTW**		x				

For peer review only

Primary outcome

Return to work

The primary outcome is time sustainable RTW, defined as a return to the employee's previous or a similar position for at least 28 consecutive days without a partial or full relapse. RTW will be assessed through monthly self-reports via the online personal accounts in the eHealth program Werk@Herstel. Participants will receive 12 monthly self-reports during the trial period. The report starts with the question, *'Are you currently completely absent from work?'*, and the participant indicates absence status with a 'yes' or 'no' and notes any work adjustments. Those not fully absent specify the percentage (%)

of work resumed. Permission will be requested to obtain this data through sick leave register systems from the OHS of the employed organisation after 12 months (end of trial). Additionally, other work-related outcomes will be similarly measured after 12 months, such as RTW in any type of work and number of sick days.

Secondary outcomes

Stress-related complaints

Stress-related complaints will be measured using the 4DSQ self-report questionnaire across the four dimensions distress, depression, anxiety, and somatisation [29]. The questionnaire consists of 50 items, measuring the severity of stress-related symptoms experienced in the past seven days. The 4DSQ is scored on a 5-point Likert scale ranging from 'no complaints' to 'very often or continuously'. The scores are converted to a 3-point scale with (0) 'no complaints' to (2) 'regularly' and upwards. The 4DSQ has demonstrated both validity and reliability in general practice, mental healthcare, and research settings (Cronbach's alpha of respective domains: 0.96; 0.96; 0.96; and 0.92) [33, 34] . The 4DSQ will be disseminated at baseline, and after 3, 6 and 12 months.

Self-reported health

Self-reported health will be measured using the Patient Reported Outcomes Measurement Information System Questionnaire (PROMIS) [35]. The PROMIS in this study consists of a short version with 10 questions measuring physical, mental and social well-being across various domains. The questions about experienced well-being in the past seven days are scored on a 5-point Likert scale ranging from (1) 'poor well-being' to (5) 'excellent well-being'. In addition, a slider ranging from (0) 'no pain' to (10) 'worst pain imaginable' can be used to indicate pain experienced in the past week. In a previous study, the PROMIS short form showed excellent internal consistency (Cronbach's alpha: >0.906) [36]. The PROMIS will be disseminated at baseline, and after 3, 6 and 12 months.

Psychosocial job characteristics

Psychosocial job characteristics will be measured by the Job Content Questionnaire (JCQ), a validated instrument for measuring psychosocial job characteristics [37]. Psychosocial job characteristics refer to the psychological and social aspects of work and the work environment. These include the mental workload, decision latitude, social support, physical demands, and job insecurity. A 4-point Likert scale, ranging from (1) 'strongly disagree' to (4) 'strongly agree', will be used to assess levels of agreements to certain statements. An example of a statement is: *'I have many opportunities to participate in decisions about what happens in my work'*. The degree of job insecurity in the past year will be asked in direct questions. The scale reliability of the JCQ is dependent of occupation, but overall acceptable

For peer review only - http://bmjopen.bmj.com/site/about/guidelines.xhtml

(Cronbach's alpha: 0.61-0.87) [38]. The JCQ will be administered at baseline, and after 3, 6 and 12 months.

Self-efficacy

Behaviour change will be assessed by means of the ASE (Attitude, Social influence and Self-Efficacy) and Return-to-Work Self-Efficacy (RTW-SE) [22, 39]. The ASE is a questionnaire that identifies the three factors influencing behaviour, specifically in the context of returning to work. For this questionnaire a 5-point Likert scale is used, ranging from (1) 'strongly agree' to (5) 'strongly disagree', for statements like *'Despite my complaints, I am going back to work, because that contributes to my recovery'*. The RTW-SE is a similar questionnaire, but focuses on an individual's self-efficacy regarding their confidence in successfully returning to work. The RTW-SE is scored on a 6-point Likert scale ranging from (1) 'strongly disagree' to (6) 'strongly agree'. The RTW-SE has proven to be of excellent internal consistency in a similar setting (Cronbach's alpha: 0.91) [40]. The ASE will be administered only at baseline, whilst the RTW-SE will be administered at baseline, as well after 3, 6 and 12 months.

Covariates

The following covariates will be collected: 1) demographic data, 2) job characteristics, 3) psychosocial risk-factors. Demographic data includes age, gender, nationality, educational level, and family situation. Job characteristics include type of work, type of employment contract (permanent or temporary), and working hours. Both demographic data and job characteristics will be administered at baseline. Psychological risk-factors will be measured by the JCQ, which will be administered at baseline and after 3, 6, and 12 months.

Process evaluation

The process and feasibility of the intervention will be assessed by means of a process evaluation to gain insight in the adoption as well as the barriers and facilitators to implementation of the intervention among the four organisations, based on the RE-AIM and PRISM frameworks [25-27]. The aim of this process evaluation is to study the adoption and implementation of the intervention to gain insight into factors that improve or restrain sustainable implementation among sick-listed workers, supervisors, and process facilitators. Therefore, the following process indicators will be studied: satisfaction, acceptability, reach, adoption, dose delivered and dose received, and penetration and sustainability among different end-users (sick-listed worker, supervisor, and process facilitator). Data will be gathered and analysed both quantitatively and qualitatively. Via logs the process measures reach, adoption, and dose delivered and received will be gathered. To illustrate, the usage of the eHealth program and attendance logs will be tracked. At approximately 9 months after commencement of the study, qualitatively the satisfaction, acceptability and barriers and facilitators to penetration and sustainability

For peer review only - http://bmjopen.bmj.com/site/about/guidelines.xhtml

of the implementation of the intervention in individual semi-structured interviews with employees (n~10), a focus group interview or individual interview with supervisors (n~6) (depending on participant preference), and a focus group interview among process facilitators (n~6) as the implementers of the intervention will be conducted. The interviews will be audio-recorded and transcribed verbatim. Analyses will be performed in SPSS/R for the quantitative measures and MAXQDA software for the qualitative measures.

Statistical analyses

Analyses will be conducted according to the intention-to-treat principle (ITT), meaning that participants will be analysed in the groups to which they were originally assigned. A descriptive analysis will be conducted for baseline characteristics and other variables, such as gender, age, educational level, occupation, and sick leave duration. Longitudinal survival analyses will be conducted to compare the effect of the intervention group with the control group over time, including multiple subgroup analyses. The Cox proportional hazard model will be employed to compute hazard ratios and standard errors will be appropriately adjusted for clustering if needed. Furthermore, confounding and/or effect modification of several factors (e.g., gender, age, education) will be checked. The quantitative data will be analysed using SPSS/R, regarding the qualitative MAXQDA will be used.

Patient and public involvement

Patients and the public were involved at several stages of this study. Prior to the start of the trial, focus groups with employees, supervisors, and management of the participating organisations were conducted. During these focus groups, barriers and insights for implementation and intervention readiness were discussed to shape the current study design [23]. Also, because the process evaluation of Volker et al. [23] found that the occupational physicians, who acted as process facilitators, provided limited support and applied the intervention only to a limited extent [22]. Therefore, in the current study, other occupational health professionals working at the participating organisation have been trained and deployed as dedicated process facilitator.

Ethics and dissemination

The trial has been approved by the Medical Ethics Committee of Amsterdam UMC, which ruled that the Medical Research Involving Human Subjects Act (WMO) does not apply to this study (approval number: 2024.0021). All participants will be informed that participation is voluntary and that they can withdraw from the study at any time. Data collected prior to withdrawal will still be used. Participants will receive both written and verbal information and will have the opportunity to ask questions prior, during and after the study. Access to participant data is restricted to the researchers directly involved in

For peer review only - http://bmjopen.bmj.com/site/about/guidelines.xhtml

the study. All participants receive an individual login code for the eHealth program. Only the researcher team members and the web administrator can access all individual accounts. At the start of the study, each participant will be assigned a unique study code under which all collected data will be stored. Identification keys will be stored in a separate location and secured with a password. The trial is registered at ClinicalTrials.gov on August 8, 2024 (trial registration number: NCT06543732). Recruitment of the study started on 6 January 2025 and the trial is expected to be completed in December 2026.

Results are expected to be published in peer-reviewed journals, and will be presented at national and international conferences. In addition, interim and final results will be shared with the participating organisations involved in this study. During committee meetings with these organisations, also the potential continuation of the intervention within their organisation will be discussed. Participants may receive updates or results of the study upon request.

Discussion

In this paper, the design of an RCT evaluating the effectiveness of the Vital@Work reintegration program for sick-listed workers with stress-related complaints, is described. Participants in the intervention group, who receive a tailored reintegration program, will be compared with a control group. This intervention combines the effective components of an eHealth program and the PA from previous research [22, 24]. By combining and using a tailored approach, we strive to fill the research gaps of effective RTW programs for workers suffering from stress-related complaints. A key strength of this study lies in its tailored study design, which allows to study different subgroups and contextual factors in participants with stress-related complaints. Another strength concerns the follow-up period in our study. Assessments take place at baseline after 3, 6 and 12 months, providing the opportunity to study participants' RTW and stress-related complaints over the long-term. This is relevant as relapse within one year after RTW frequently occurs [41, 42], and thus evaluating the intervention's effectiveness in the long-term is significant. A few limitations should also be considered. One of the limitations is that the researchers and the process facilitators cannot be blinded due to intervention allocation. However, all involved parties will be instructed not to disclose the research design to the participants, ensuring that the participants remain blinded as the control group is provided with general information via the eHealth program. Another limitation is that the outcome measures are based on self-reported questionnaires (4DSQ, PROMIS, JCQ, ASE, and RTW-SE). Participants may experience recall problems with the monthly reports and a response bias may occur. Hence, to limit potential recall bias for the primary outcome (time to sustainable return to work), sick leave data from the register systems of the OHS of the employed organisation will be obtained (when permission is granted). By using both self-reported and register data, validity could be increased [43].

For peer review only - http://bmjopen.bmj.com/site/about/guidelines.xhtml

Authors' contributions

All authors will participate in the collection and execution of the trial; EEMvD and TTJ are primary executers. Authors AWdS, RMCS, ATFB, JRA and TTJ were responsible for the development of the trial intervention, with AWdS writing the non-WMO declaration. Authors RMCS, ATFB, and JRA obtained funding for this study. Author EEMvD wrote the first version of the paper; supervised by TTJ. Author TTJ provided content on the process evaluation. All authors have reviewed and approved the final manuscript.

Funding

This work was supported by Health~Holland, reference number LSHM22035-H025. The Vital@Work project is part of a PPP Allowance, made available by Health~Holland, Top Sector Life Sciences & Health, to stimulate public-private partnerships (https://www.health-holland.com). All involved organisations in the PPP (Amsterdam UMC, GGZ inGeest, Transavia, Rijksschoonmaakorganisatie) contributed in kind. Rijksschoonmaakorganisatie also contributed in cash.

Competing interests

JRA is consultant and certificate-holder of a spin-out company for implementation of the mobile application concerning the IkHerstel intervention in the Netherlands. JRA holds a chair in insurance medicine paid by the Dutch Social Security Agency; and has received grants from ZonMw, Nederlandse Organisatie voor Wetenschappelijk Onderzoek, Instituut Gak, Uitvoeringsinstituut Werknemersverzekeringen, Sociale Zaken en Werkgelegenheid, VWS (Volksgezondheid, Welzijn en Sport), Pfizer, Achmea, CVZ (College voor Zorgverzekeringen), and Zorginstituut; all outside the submitted work. The other authors have no competing interests.

References

1. WHO. World mental health today: latest data. World Health Organization; 2025. Report No.: 9240113819.

2. WHO. World mental health report: Transforming mental health for all. World Health Organization; 2022. Report No.: 9240049339.

3. Niedhammer I, Bertrais S, Witt K. Psychosocial work exposures and health outcomes: a meta-review of 72 literature reviews with meta-analysis. Scandinavian journal of work, environment & health. 2021;47(7):489.

4. de Oliveira C, Saka M, Bone L, Jacobs R. The role of mental health on workplace productivity: a critical review of the literature. Applied health economics and health policy. 2023;21(2):167–93.

5. Wolvetang S, van Dongen JM, Speklé E, Coenen P, Schaafsma F. Sick leave due to stress, what are the costs for Dutch employers? Journal of Occupational Rehabilitation. 2022;32(4):764–72.

For peer review only - http://bmjopen.bmj.com/site/about/guidelines.xhtml

6. VZinfo. Ziekteverzuim | Naar oorzaak 2025 [Available from: https://www.vzinfo.nl/ziekteverzuim/naar-oorzaak.

7. Hassard J, Teoh KRH, Visockaite G, Dewe P, Cox T. The cost of work-related stress to society: A systematic review. Journal of occupational health psychology. 2018;23(1):1.

8. Chisholm D, Sweeny K, Sheehan P, Rasmussen B, Smit F, Cuijpers P, et al. Scaling-up treatment of depression and anxiety: a global return on investment analysis. The Lancet Psychiatry. 2016;3(5):415–24.

9. Van den Heuvel SG, Fernandez Beiro L, Van Dam LMC. Nationale enquête arbeidsomstandigheden 2021: Resultaten in vogelvlucht. TNO & CBS; 2022.

10. Goh J, Pfeffer J, Zenios SA. The relationship between workplace stressors and mortality and health costs in the United States. Management Science. 2016;62(2):608–28.

11. Vogel N, Schandelmaier S, Zumbrunn T, Ebrahim S, de Boer WEL, Busse JW, et al. Return-to-work coordination programmes for improving return to work in workers on sick leave. Cochrane Database of Systematic Reviews. 2017(3).

12. Prinz C. Sickness, disability and work: lessons from reforms and lack of change across the OECD countries. 2010.

13. Mikkelsen MB, Rosholm M. Systematic review and meta-analysis of interventions aimed at enhancing return to work for sick-listed workers with common mental disorders, stress-related disorders, somatoform disorders and personality disorders. Occupational and environmental medicine. 2018;75(9):675–86.

14. Tingulstad A, Meneses-Echavez J, Evensen LH, Bjerk M, Berg RC. Effectiveness of work-related interventions for return to work in people on sick leave: a systematic review and meta-analysis of randomized controlled trials. Systematic Reviews. 2022;11(1):192.

15. Blonk RWB, Brenninkmeijer V, Lagerveld SE, Houtman ILD. Rehabilitation in case of work-related psychological complaints: A field experiment among the self-employed. Work and Stress. 2006;20(2):129–44.

16. Dalgaard VL, Aschbacher K, Andersen JH, Glasscock DJ, Willert MV, Carstensen O, et al. Return to work after work-related stress: a randomized controlled trial of a work-focused cognitive behavioral intervention. Scandinavian Journal of Work, Environment & Health. 2017:436–46.

17. Hedlund Å, Kristofferzon M-L, Boman E, Nilsson A. Are return to work beliefs, psychological well-being and perceived health related to return-to-work intentions among women on long-term sick leave for common mental disorders? A cross-sectional study based on the theory of planned behaviour. BMC public health. 2021;21(1):535.

18. Buys NJ, Selander J, Sun J. Employee experience of workplace supervisor contact and support during long-term sickness absence. Disability and Rehabilitation. 2019;41(7):808–14.

19. Netterstrøm B, Friebel L, Ladegaard Y. Effects of a multidisciplinary stress treatment programme on patient return to work rate and symptom reduction: results from a randomised, wait-list controlled trial. Psychotherapy and psychosomatics. 2013;82(3):177–86.

20. Martin MHT, Nielsen MBD, Madsen IEH, Petersen SMA, Lange T, Rugulies R. Effectiveness of a coordinated and tailored return-to-work intervention for sickness absence beneficiaries with mental health problems. Journal of occupational rehabilitation. 2013;23(4):621–30.

For peer review only - http://bmjopen.bmj.com/site/about/guidelines.xhtml

21. Bültmann U, Sherson D, Olsen J, Hansen CL, Lund T, Kilsgaard J. Coordinated and tailored work rehabilitation: a randomized controlled trial with economic evaluation undertaken with workers on sick leave due to musculoskeletal disorders. Journal of occupational rehabilitation. 2009;19(1):81–93.

22. Volker D, Zijlstra-Vlasveld MC, Anema JR, Beekman ATF, Brouwers EPM, Emons WHM, et al. Effectiveness of a blended web-based intervention on return to work for sick-listed employees with common mental disorders: results of a cluster randomized controlled trial. Journal of medical Internet research. 2015;17(5):e116.

23. Volker D, Zijlstra-Vlasveld MC, Brouwers EPM, Van der Feltz-Cornelis CM. Process evaluation of a blended web-based intervention on return to work for sick-listed employees with common mental health problems in the occupational health setting. Journal of Occupational Rehabilitation. 2017;27(2):186–94.

24. van Oostrom SH, van Mechelen W, Terluin B, de Vet HCW, Knol DL, Anema JR. A workplace intervention for sick-listed employees with distress: results of a randomised controlled trial. Occupational and environmental medicine. 2010;67(9):596–602.

25. Gaglio B, Shoup JA, Glasgow RE. The RE-AIM framework: a systematic review of use over time. American journal of public health. 2013;103(6):e38–e46.

26. Feldstein AC, Glasgow RE. A practical, robust implementation and sustainability model (PRISM) for integrating research findings into practice. The joint commission journal on quality and patient safety. 2008;34(4):228–43.

27. Glasgow RE, Vogt TM, Boles SM. Evaluating the public health impact of health promotion interventions: the RE-AIM framework. American journal of public health. 1999;89(9):1322–7.

28. Chan A-W, Boutron I, Hopewell S, Moher D, Schulz KF, Collins GS, et al. SPIRIT 2025 statement: updated guideline for protocols of randomised trials. The Lancet. 2025;405(10491):e19–e27.

29. Terluin B, van Marwijk HWJ, Adèr HJ, de Vet HCW, Penninx BWJH, Hermens MLM, et al. The Four-Dimensional Symptom Questionnaire (4DSQ): a validation study of a multidimensional self-report questionnaire to assess distress, depression, anxiety and somatization. BMC psychiatry. 2006;6(1):34.

30. NVAB. Richtlijn Psychische problemen. 2019.

31. Terluin B, Oosterbaan DB, Brouwers EP, Van Straten A, Van de Ven PM, Langerak W, et al. To what extent does the anxiety scale of the Four-Dimensional Symptom Questionnaire (4DSQ) detect specific types of anxiety disorder in primary care? A psychometric study. BMC psychiatry. 2014;14(1):121.

32. CastorEDC. 2024 [Available from: https://www.castoredc.com/].

33. Kleinstäuber M, Exner A, Lambert MJ, Terluin B. Validation of the Four-Dimensional Symptom Questionnaire (4DSQ) in a mental health setting. Psychology, health & medicine. 2021;26(sup1):1–19.

34. Terluin B, Verbraak M. Validiteit van de Vierdimensionale Klachtenlijst (4DKL) in de ambulante geestelijke gezondheidszorg. Tijdschrift voor klinische psychologie. 2014;44(3):198–211.

35. Terwee CB, Roorda LD, De Vet HCW, Dekker J, Westhovens R, Van Leeuwen J, et al. Dutch–Flemish translation of 17 item banks from the patient-reported outcomes measurement information system (PROMIS). Quality of Life Research. 2014;23(6):1733–41.

For peer review only - http://bmjopen.bmj.com/site/about/guidelines.xhtml

36. Bartlett SJ, Orbai A-M, Duncan T, DeLeon E, Ruffing V, Clegg-Smith K, et al. Reliability and validity of selected PROMIS measures in people with rheumatoid arthritis. PloS one. 2015;10(9):e0138543.

37. Karasek R, Brisson C, Kawakami N, Houtman I, Bongers P, Amick B. The Job Content Questionnaire (JCQ): an instrument for internationally comparative assessments of psychosocial job characteristics. Journal of occupational health psychology. 1998;3(4):322.

38. Choi B, Kurowski A, Bond M, Baker D, Clays E, De Bacquer D, et al. Occupation-differential construct validity of the Job Content Questionnaire (JCQ) psychological job demands scale with physical job demands items: a mixed methods research. Ergonomics. 2012;55(4):425–39.

39. de Rijk A, Janssen N, van Lierop B, Alexanderson K, Nijhuis F. A behavioral approach to RTW after sickness absence: the development of instruments for the assessment of motivational determinants, motivation and key actors' attitudes. Work. 2009;33(3):273–85.

40. Gjengedal RGH, Lagerveld SE, Reme SE, Osnes K, Sandin K, Hjemdal O. The return-to-work self-efficacy questionnaire (RTW-SE): a validation study of predictive abilities and cut-off values for patients on sick leave due to anxiety or depression. Journal of occupational rehabilitation. 2021;31(3):664–73.

41. Koopmans PC, Bültmann U, Roelen CAM, Hoedeman R, van der Klink JJL, Groothoff JW. Recurrence of sickness absence due to common mental disorders. International archives of occupational and environmental health. 2011;84(2):193–201.

42. Spronken M, Brouwers EPM, Vermunt JK, Arends I, Oerlemans WGM, Van Der Klink JJL, et al. Identifying return to work trajectories among employees on sick leave due to mental health problems using latent class transition analysis. BMJ open. 2020;10(2):e032016.

43. Lavreysen O, Bakusic J, Abatzi T-A, Geerts A, Mateusen M, Bashkin O, et al. An overview of work-related stress assessment. Journal of affective disorders. 2025.

Figure legends

Fig. 1 Decision aid of tailored eHealth pathways intervention group

Figure 1 displays a simplified representation of eHealth pathways. The pathways are determined on participants' randomisation, followed by the two navigating questions and 4DSQ. Cut-off scores are used to determine the tailored pathways [29, 31]. The figure was created by EEMvD using Lucidchart.

For peer review only - http://bmjopen.bmj.com/site/about/guidelines.xhtml

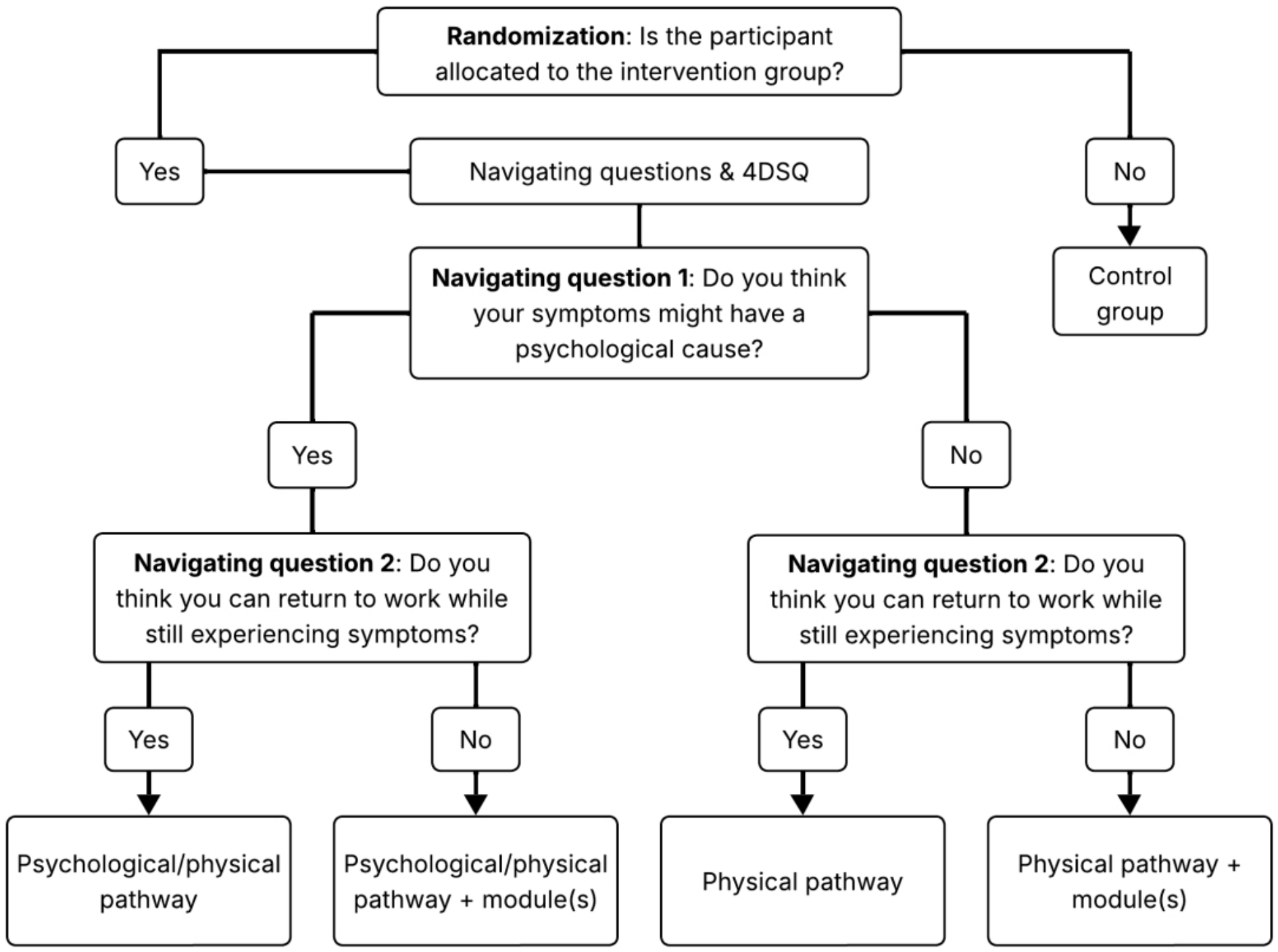

Fig. 1 Decision aid of tailored eHealth pathways intervention group

500x379mm (118 x 118 DPI)

SPIRIT 2025 checklist of items to address in a randomized trial protocol*

Section / Topic	No	SPIRIT 2025 checklist item description	Reported on page no.
**Administrative information**			
Title and structured summary	1a	Title stating the trial design, population, and interventions, with identification as a protocol	1
	1b	Structured summary of trial design and methods, including items from the World Health Organization Trial Registration Data Set	1-2
Protocol version	2	Version date and identifier	2
Roles and responsibilities	3a	Names, affiliations, and roles of protocol contributors	1 and 14
	3b	Name and contact information for the trial sponsor	14
	3c	Role of trial sponsor and funders in design, conduct, analysis, and reporting of trial; including any authority over these activities	14
	3d	Composition, roles, and responsibilities of the coordinating site, steering committee, endpoint adjudication committee, data management team, and other individuals or groups overseeing the trial, if applicable	14
**Open science**			
Trial registration	4	Name of trial registry, identifying number (with URL), and date of registration. If not yet registered, name of intended registry	2 and 13
Protocol and statistical analysis plan	5	Where the trial protocol and statistical analysis plan can be accessed	13
Data sharing	6	Where and how the individual de-identified participant data (including data dictionary), statistical code, and any other materials will be accessible	13
Funding and conflicts of interest	7a	Sources of funding and other support (e.g., supply of drugs)	14
	7b	Financial and other conflicts of interest for principal investigators and steering committee members	14
Dissemination policy	8	Plans to communicate trial results to participants, healthcare professionals, the public, and other relevant groups (e.g., reporting in trial registry, plain language summary, publication)	12-13
**Introduction**			
Background and rationale	9a	Scientific background and rationale, including summary of relevant studies (published and unpublished) examining benefits and harms for each intervention	2-4
	9b	Explanation for choice of comparator	2-4
Objectives	10	Specific objectives related to benefits and harms	12-13
**Methods: Patient and public involvement, trial design**			
Patient and public involvement	11	Details of, or plans for, patient or public involvement in the design, conduct, and reporting of the trial	12-13
Trial design	12	Description of trial design including type of trial (e.g., parallel group, crossover), allocation ratio, and framework (e.g., superiority, equivalence, non-inferiority, exploratory)	4

For peer review only - http://bmjopen.bmj.com/site/about/guidelines.xhtml

**Methods: Participants, interventions, and outcomes**			
Trial setting	13	Settings (e.g., community, hospital) and locations (e.g., countries, sites) where the trial will be conducted	4
Eligibility criteria	14a	Eligibility criteria for participants	4
	14b	If applicable, eligibility criteria for sites and for individuals who will deliver the interventions (e.g., surgeons, physiotherapists)	5-6
Intervention and comparator	15a	Intervention and comparator with sufficient details to allow replication including how, when, and by whom they will be administered. If relevant, where additional materials describing the intervention and comparator (e.g., intervention manual) can be accessed	5-8
	15b	Criteria for discontinuing or modifying allocated intervention/comparator for a trial participant (e.g., drug dose change in response to harms, participant request, or improving/worsening disease)	n/a
	15c	Strategies to improve adherence to intervention/comparator protocols, if applicable, and any procedures for monitoring adherence (e.g., drug tablet return, sessions attended)	5-7
	15d	Concomitant care that is permitted or prohibited during the trial	n/a
Outcomes	16	Primary and secondary outcomes, including the specific measurement variable (e.g., systolic blood pressure), analysis metric (e.g., change from baseline, final value, time to event), method of aggregation (e.g., median, proportion), and time point for each outcome	9-11
Harms	17	How harms are defined and will be assessed (e.g., systematically, non-systematically)	n/a
Participant timeline	18	Time schedule of enrollment, interventions (including any run-ins and washouts), assessments, and visits for participants. A schematic diagram is highly recommended (see Figure)	9 (table 1)
Sample size	19	How sample size was determined, including all assumptions supporting the sample size calculation	8
Recruitment	20	Strategies for achieving adequate participant enrollment to reach target sample size	5-6 and 8
**Methods: Assignment of interventions**			
Randomization:			
Sequence generation	21a	Who will generate the random allocation sequence and the method used	8
	21b	Type of randomization (simple or restricted) and details of any factors for stratification. To reduce predictability of a random sequence, other details of any planned restriction (e.g., blocking) should be provided in a separate document that is unavailable to those who enroll participants or assign interventions	8
Allocation concealment mechanism	22	Mechanism used to implement the random allocation sequence (e.g., central computer/telephone; sequentially numbered, opaque, sealed containers), describing any steps to conceal the sequence until interventions are assigned	8
Implementation	23	Whether the personnel who will enroll and those who will assign participants to the interventions will have access to the random allocation sequence	8
Blinding	24a	Who will be blinded after assignment to interventions (e.g., participants, care providers, outcome assessors, data analysts)	5 and 8

For peer review only - http://bmjopen.bmj.com/site/about/guidelines.xhtml

For peer review only

	24b	If blinded, how blinding will be achieved and description of the similarity of interventions	5 and 8
	24c	If blinded, circumstances under which unblinding is permissible, and procedure for revealing a participant's allocated intervention during the trial	n/a
**Methods: Data collection, management, and analysis**			
Data collection methods	25a	Plans for assessment and collection of trial data, including any related processes to promote data quality (e.g., duplicate measurements, training of assessors) and a description of trial instruments (e.g., questionnaires, laboratory tests) along with their reliability and validity, if known. Reference to where data collection forms can be accessed, if not in the protocol	9-12
	25b	Plans to promote participant retention and complete follow-up, including list of any outcome data to be collected for participants who discontinue or deviate from intervention protocols	n/a
Data management	26	Plans for data entry, coding, security, and storage, including any related processes to promote data quality (e.g., double data entry; range checks for data values). Reference to where details of data management procedures can be accessed, if not in the protocol	12-13
Statistical methods	27a	Statistical methods used to compare groups for primary and secondary outcomes, including harms	12
	27b	Definition of who will be included in each analysis (e.g., all randomized participants), and in which group	12
	27c	How missing data will be handled in the analysis	12
	27d	Methods for any additional analyses (e.g., subgroup and sensitivity analyses)	12
**Methods: Monitoring**			
Data monitoring committee	28a	Composition of data monitoring committee (DMC); summary of its role and reporting structure; statement of whether it is independent from the sponsor and funder; conflicts of interest and reference to where further details about its charter can be found, if not in the protocol. Alternatively, an explanation of why a DMC is not needed	14
	28b	Explanation of any interim analyses and stopping guidelines, including who will have access to these interim results and make the final decision to terminate the trial	12-13
Trial monitoring	29	Frequency and procedures for monitoring trial conduct. If there is no monitoring, give explanation	6-7
**Ethics**			
Research ethics approval	30	Plans for seeking research ethics committee/institutional review board approval	12-13
Protocol amendments	31	Plans for communicating important protocol modifications to relevant parties	n/a
Consent or assent	32a	Who will obtain informed consent or assent from potential trial participants or authorized proxies, and how	5
	32b	Additional consent provisions for collection and use of participant data and biological specimens in ancillary studies, if applicable	5
Confidentiality	33	How personal information about potential and enrolled participants will be collected, shared, and maintained in order to protect confidentiality before, during, and after the trial	12-13
Ancillary and post-trial care	34	Provisions, if any, for ancillary and post-trial care, and for compensation to those who suffer harm from trial participation	n/a

For peer review only - http://bmjopen.bmj.com/site/about/guidelines.xhtml

*We strongly recommend reading this checklist in conjunction with the SPIRIT 2025 Explanation and Elaboration and the SPIRIT 2025 Expanded Checklist for important clarifications on all the items. We also recommend reading relevant SPIRIT extensions. See www.consort-spirit.org

Citation: Chan A-W, Boutron I, Hopewell S, Moher D, Schulz KF, et al. SPIRIT 2025 statement: updated guideline for protocols of randomised trials. BMJ 2025;389:e081477. https://dx.doi.org/10.1136/bmj-2024-081477

© 2025 Chan A-W et al. This is an Open Access article distributed under the terms of the Creative Commons Attribution License (https://creativecommons.org/licenses/by/4.0/), which permits unrestricted use, distribution, and reproduction in any medium, provided the original work is properly cited.

For peer review only

For peer review only - http://bmjopen.bmj.com/site/about/guidelines.xhtml

Informed consent form (participant)

Vital@Work: a re-integration programme for employees with stress-related complaints

- I have read the information letter. I was also able to ask questions. My questions have been sufficiently answered. I had enough time to decide whether I want to participate.
- I understand that participation is voluntary. I also understand that I can withdraw from the study at any time. I do not have to provide a reason for this.
- I give permission for my data to be collected and used in the manner and for the purposes as described in the information letter.
- I give permission for my data to be stored within Amsterdam UMC for another 10 years after this study.
- I would like to participate in this study.

I give permission for the collection of my data:

O yes
O no

Date of permission (dd-mm-yyyy):

____

I give permission for requesting my (partial) recovery notifications from the absence database at my occupational health service:

O yes
O no

I give permission to inform my occupational physician about my participation in the study:

O yes
O no

I give permission to be contacted to take part in an interview:

O yes
O no

I give permission to be contacted again after this study for follow-up research:

O yes
O no

For peer review only - http://bmjopen.bmj.com/site/about/guidelines.xhtml

Vital@Work reintegration program for sick-listed workers with stress-related complaints in Occupational Healthcare in the Netherlands: study protocol of a randomised controlled trial

Journal:	*BMJ Open*
Manuscript ID	bmjopen-2026-116576.R2
Article Type:	Protocol
Date Submitted by the Author:	02-Sep-2026
Complete List of Authors:	van Doeveren, Eva; Amsterdam UMC Locatie AMC, Department Public and Occupational Health; Amsterdam Public Health Research Institute, Societal Participation and Health<br>de Smalen, Allard; Amsterdam UMC location Vrije Universiteit Amsterdam, Department Public and Occupational Health; Amsterdam Public Health Research Institute, Societal Participation and Health<br>Schelvis, Roosmarijn; Amsterdam Public Health Research Institute, Societal Participation and Health, Amsterdam; Amsterdam UMC location University of Amsterdam, Department Public and Occupational Health<br>Beekman, Aartjan ; Amsterdam Public Health Research Institute, Societal Participation and Health, Amsterdam; Amsterdam University Medical Centre location VUmc, Department of Psychiatry<br>Anema, Johannes R.; Amsterdam UMC location Vrije Universiteit Amsterdam, Department Public and Occupational Health; Amsterdam Public Health Research Institute, Societal Participation and Health<br>Juurlink, Trees T; Amsterdam UMC location Vrije Universiteit Amsterdam, Department Public and Occupational Health ; Amsterdam Public Health Research Institute, Societal Participation and Health
**Primary Subject Heading**:	Occupational and environmental medicine
Secondary Subject Heading:	Mental health, Public health
Keywords:	MENTAL HEALTH, eHealth, Health Workforce, Occupational Health Services, Occupational Stress, Workplace

SCHOLARONE MANUSCRIPTS

STUDY PROTOCOL

Vital@Work reintegration program for sick-listed workers with stress-related complaints in Occupational Healthcare in the Netherlands: study protocol of a randomised controlled trial

Eva E. M. van Doeveren[1,2], Allard W. de Smalen[1,2], Roosmarijn M. C. Schelvis[2,3], Aartjan T. F. Beekman[2,4], Johannes R. Anema[1,2] and Trees T. Juurlink[1,2]

Author affiliations:

[1]Amsterdam UMC location Vrije Universiteit Amsterdam, Department Public and Occupational Health, De Boelelaan 1117, Amsterdam, The Netherlands

[2]Amsterdam Public Health Research Institute, Societal Participation and Health, Amsterdam, The Netherlands

[3] Amsterdam UMC location University of Amsterdam, Department Public and Occupational Health, Meibergdreef 9, Amsterdam, The Netherlands,

[4]Amsterdam University Medical Centre location VUmc, Department of Psychiatry, De Boelelaan 1117, Amsterdam, The Netherlands

For peer review only

Abstract

Introduction: Mental health problems, including stress-related complaints, are an increasing concern in today's workforce. Therefore, long term sickness absence becomes a risk, yet effective return to work (RTW) interventions for employees suffering from mental health problems are scarce and they lack tailoring. The aim of this study is to describe the design of a randomised controlled trial (RCT) evaluating the effectiveness of the tailored Vital@Work reintegration program for sick-listed workers with stress-related complaints.

Methods and analysis: An RCT involving 208 sick-listed workers with stress-related complaints will be conducted with measurements taking place at baseline, and after 3, 6 and 12 months. Stress-related complaints will be assessed with a distress screener. All participants will receive care as usual (CAU). The intervention group will receive a tailored eHealth program, followed by the participatory approach (PA) if full RTW has not been achieved after 6 weeks in the study. Participants assigned to the control group will be provided with general information via the eHealth program. The primary outcome is sustainable RTW, defined as lasting at least 28 consecutive days without any relapse. Secondary outcomes are stress-related complaints, psychosocial job characteristics, and behaviour change. A mixed-methods approach will be used for the process evaluation.

Ethics and dissemination: This study has been approved by the Medical Ethics Committee of Amsterdam UMC (approval number: 2024.0021). Results are expected to be published in peer-reviewed journals. In addition, interim and final results will be shared and discussed with the participating organisations involved in this study.

Trial registration number: NCT06543732.

Strengths and limitations of this study

- The use of standardised outcome measures, such as the 4DSQ, PROMIS and JCQ, enhances the validity and reliability of our study findings.
- The mixed-methods approach provides insights in strengths and limitations for sustainable implementation of the intervention within different workplace contexts.
- The long-term follow-up of one-year offers the opportunity to examine sustainable return to work.
- Only single blinding of participants is possible due to the nature of the intervention.

Keywords: Mental health, Return to work, Workplace intervention, eHealth, Participatory Approach, Randomised controlled trial

Protocol version: 2 September 2026, version 1.2

Introduction

In 2021, more than one billion people, representing about 14% of the global population, experienced poor mental health [1]. This number is likely to increase in the coming years due to growing economic pressure, insufficient access to mental health services, and poor psychosocial working conditions (e.g. high workload and lack of support) [1-3]. Mental health problems have a profound impact on the working society. On an individual scale, they are associated with low productivity and long-term sickness absence [4, 5]. Mental health problems were even among the leading causes of long-term sickness absence in the Netherlands in 2023, constituting 40% of all absenteeism cases, of which 29% were attributable to stress-related complaints [6]. From a societal perspective, the most prominent consequence of mental health complaints is the financial burden. [5, 7]. For instance, anxiety and depressive complaints result in an estimated annual loss of 12 billion productive workdays across 36 countries, which together represent 80% of the global population [1, 8]. In the Netherlands, employers spent approximately €3.3 billion in 2021 on sickness absence costs caused by work stress alone [5, 7, 9]. In the United States the estimated costs amounted to US$187 billion [5, 9, 10]. Moreover, prolonged absence from work is associated with a lower likelihood of eventual return to work (RTW), and a higher risk of depending on social benefits or choosing early retirement [11, 12]. This highlights the need for effective support throughout sickness absence.

For peer review only - http://bmjopen.bmj.com/site/about/guidelines.xhtml

Several interventions aimed at facilitating RTW of sick-listed workers have been developed and evaluated [13-16]. The systematic review by Mikkelsen & Rosholm [13] showed that interventions at the workplace are strong predictors for faster RTW of sick-listed workers with mental health complaints. Also integrating multiple components, such as workplace contact combined with therapy, resulted in a faster RTW [13]. Workplace involvement can thus lay the foundation for successful RTW at a later stage, as early contact initiatives are perceived as supportive by sick-listed workers [17, 18]. Additionally, the length of sickness absence is an important factor in determining intervention's effectiveness. In a review examining the effectiveness of interventions for individuals at risk or on long-term sickness absence, Tingulstad et al. [14] reported a considerable variation in sickness absence length across included studies, ranging from 1 to 24 months. The authors concluded that RTW rates were high within the first 8 weeks of sickness absence but gradually declined after 12 weeks, suggesting that early RTW efforts may be critical for successful reintegration [14]. Notably, participants in this review could have been absent for any reason and only two of the included studies specifically addressed workers with stress-related complaints. The interventions in these studies, workplace-based cognitive behavioural therapy (CBT) and stress management, showed a potentially positive effect on RTW, although the long-term impact remained relatively small [16, 19]. Furthermore, RTW interventions have shown effectiveness for workers with musculoskeletal disorders, but no evidence has yet been found for workers reporting (stress-related) mental health complaints [20, 21].

The present study will therefore combine effective elements of previous studies to support the RTW of sick-listed workers with stress-related complaints. It is grounded in two evidence-based interventions, which are subsequently explained: an eHealth program and the Participatory Approach (PA). The eHealth program is based on the Return@Work intervention by Volker et al. [22] and embodies the principles of CBT and problem solving therapy (PST) [22]. In this study, sick-listed participants in the eHealth program group showed a faster time to first RTW than participants in the control group receiving only care as usual (CAU). No significant effects were found for time to full RTW [22, 23]. The PA on the other hand, intervenes at the early onset of sickness absence, and involves a structured process between the sick-listed worker and supervisor guided by an independent process leader to establish a consensus-based plan to facilitate RTW [24]. A previous study evaluating the effectiveness of the PA for sick-listed workers with distress found no overall effect on RTW [24]. However, the PA was effective in facilitating RTW among a subgroup of workers who, at baseline, reported a positive intention to resumption of work, despite the presence of stress-related complaints [17, 24].

Therefore, this paper describes the design of the Vital@Work reintegration program. This program combines the effective elements of the eHealth program and the PA to improve sustainable RTW among sick-listed workers with stress-related complaints. Based on a triage assessment, the reintegration program is tailored for each participant in the intervention group. It is hypothesised that participants receiving the tailored reintegration program show a faster and more sustainable return to

For peer review only - http://bmjopen.bmj.com/site/about/guidelines.xhtml

work as compared to the participants receiving the control condition. Next to the effectiveness evaluation, a process evaluation will be conducted to describe intervention adoption and understand barriers and facilitators to implementation across contexts.

Methods and analysis

Study design

This study is a two-arm randomised controlled trial (RCT) to evaluate the effectiveness of the Vital@Work reintegration program for sick-listed workers with stress-related complaints. The study compares participants in the intervention group, who receive the reintegration program, with a group receiving a control condition. All participants will receive CAU. Participants in both groups will be monitored for one year with measurements taking place at baseline, and after 3, 6 and 12 months. In the last phase of the study period, a process evaluation will be conducted following the Reach, Effectiveness, Adoption, Implementation and Maintenance (RE-AIM), and Practical, Robust Implementation and Sustainability Model (PRISM) frameworks [25-27]. In the process evaluation, the process and feasibility of the Vital@Work reintegration program will be assessed to gain insight in the adoption, and barriers and facilitators for implementation of the intervention. The Standard Protocol Items: Recommendations for Interventional Trials (SPIRIT) guidelines were followed while writing this protocol (supplemental material 1) [28].

Study population and setting

The study population consists of sick-listed workers across four participating organisations in the Netherlands. The organisations differ in size, sector (aviation, cleaning, hospital care and psychiatric healthcare), type (private or public), employee characteristics and target groups (e.g. unskilled, practically trained or theoretically trained). These organisations represent a diverse group of sick-listed workers, allowing for research into which aspects of the intervention work for whom and under which conditions. Inclusion criteria for participants at enrolment of the study are: 1) on sickness absence between 2-12 weeks, 2) aged between 18-68 years, 3) having an employment contract for ≥12 hours per week, 4) suffering from stress-related complaints assessed by a distress screener. Exclusion criteria are: 1) suffering from severe psychiatric disorders (e.g. suicidality, schizophrenia) or other chronic conditions (e.g. terminal illness) that substantially intervenes with RTW, 2) insufficient Dutch language skills to complete eHealth modules and/or questionnaires, 3) in legal conflict with the employer, 4) pregnancy.

For peer review only - http://bmjopen.bmj.com/site/about/guidelines.xhtml

Recruitment procedure

Workers who have been on sickness absence for 2-12 weeks will automatically receive an invitation email from their organisation's Occupational Health Service (OHS). Every 1-2 weeks, such an email will be sent to newly sick-listed workers. The email contains brief information about the study and the sick-listed workers can leave their contact details via an online link when interested to participate. Potential participants will then receive a screening questionnaire sent to their email address, including a distress screener, in which the inclusion and exclusion criteria are assessed. The distress screener comprises three questions derived from the Four-Dimensional Symptom Questionnaire (4DSQ), a validated instrument for identifying distress in sick-listed workers [29]. The questions, which focus on stress-related complaints experienced during the past week, are rated on a scale from (0) 'never' to (4) 'constantly' and a worker meets the inclusion requirement if ≥3 points are scored. If the potential participant also fits the other inclusion criteria, a researcher will contact the potential participant by phone to provide further details about study participation. If the potential participant verbally agrees, an information letter and informed consent form will be sent by email (supplemental material 2). Participation is official once the informed consent is digitally signed, after which the participant gains access to the eHealth program and the baseline measurement questionnaires via a personal account.

Intervention

Each participant, regardless of intervention or control group, will receive CAU from their organisation's OHS. In the Dutch context, under the Dutch Gatekeeper Improvement Act, CAU consists of employees' entitlement to occupational healthcare, whereby an occupational physician conducts a problem analysis and provides guidance based on the employee's medical condition, to facilitate (eventual) return to work. Participants assigned to the intervention group will, in addition to CAU by the occupational healthcare provider and eventual other care or treatment they receive for their condition, receive the Vital@Work reintegration program, consisting of a tailored eHealth program (see below under *eHealth program)* and followed by the PA (see below under *Participatory approach)* if full RTW has not been achieved after 6 weeks. To ensure blinding, participants in the control group are provided with general information via the eHealth program, which includes links to existing websites about health and RTW. All participants receive a personal Werk@Herstel account for the eHealth program and questionnaires.

Process facilitators

The intervention group will be guided by a designated and trained process facilitator, who acts as a primary contact person and facilitator during the intervention period. The intervention period starts at enrolment of the study and will last until the PA is finished, typically after 3-4 months. The process facilitators are occupational health practitioners (e.g. case manager, occupational nurse, company social worker) employed at the participating organisations. Prior to the start of the intervention, the process

For peer review only - http://bmjopen.bmj.com/site/about/guidelines.xhtml

facilitator attends a day-long training organised by the researchers. The training includes a detailed explanation of the content of the eHealth program, the PA components, and role-play scenario's to practice the facilitators role. Furthermore, the process facilitator adheres to a predefined protocol, and remains neutral towards both the participant and supervisor. Process facilitators supporting participants in this study will not deliver CAU to these participant while administering the intervention.

eHealth program

The eHealth program is based on the original Return@Work intervention, and adapted for the current study by incorporating the Dutch guidelines for employees with common mental disorders [22, 30]. Participants will follow either a psychological or a physical pathway, with or without extra psychoeducational, cognitive behavioural and/or problem solving modules, depending on their scores on navigating questions at baseline. Figure 1 displays a simplified representation of the navigation of eHealth pathways. The eHealth pathways are determined on participants' responses to two navigating questions and the 4DSQ in the baseline measurement. The 4DSQ is a self-report questionnaire designed to assess stress-related complaints across four dimensions: distress, depression, anxiety, and somatisation [29]. For each dimension, previously used cut-off scores are used to determine the tailored pathways [29, 31]. Depending on their responses, the participants may also receive psychoeducational, cognitive behavioural and/or problem solving modules 'helping thoughts' and/or 'RTW with physical complaints' from the other pathway. Pathways are individually tailored, as it is expected this will lead to a higher adherence within the intervention.

The eHealth modules, based on the tailored pathways, potentially encompass the following courses:

- Psychoeducation on support for (work-related) psychological complaints,
- Problem solving exercises for a bottleneck analysis by mapping work activities and related hindering factors,
- Problem solving exercises using a stepwise approach to identify and cope with RTW barriers and other hindering factors at work,
- Information and exercises for early detection and/or maintaining good mental health,
- Cognitive behavioural exercises to identify (un)helpful thinking and reframing unhelpful thoughts, based on the CBT principles *(extra module)*,
- Information about RTW with physical complaints and the impact of poor mental health on physical health *(extra module)*.

Regardless of tailored pathway, all participants receive eHealth modules focusing on psychoeducation, perceptions of RTW including problem-solving exercises, relapse prevention and continuous monitoring. The modules serve as preparation for formulating a RTW plan, which is addressed in the

For peer review only - http://bmjopen.bmj.com/site/about/guidelines.xhtml

PA that is offered if one is not fully returned to work after 6 weeks. Hence, participants can use their responses in the modules during the PA. For those fully resumed to work within 6 weeks, the eHealth program and RTW plan can serve as reflection and useful reference in case of relapse. The participants progress through the eHealth program independently and each module must be completed before progressing to the next one. The assigned process facilitator will reach out 2 weeks after start to check in on their progress and encourage module completion. Ideally, the eHealth program is completed within 6 weeks after start. After 6 weeks, the participants are asked if they have returned to work or not. If participants have not yet fully returned to work, the PA is started regardless of completion of the eHealth modules.

Participatory approach

After 6 weeks in the eHealth program, all participants not returned to work receive the PA. The PA is a stepwise process between the sick-listed worker (participant) and their supervisor aimed at identifying obstacles for RTW, and subsequently developing a consensus-based RTW plan. The process facilitator will guide this process to enhance equal participation. Also in the PA tailoring is applied, as two variants of the PA are offered. That is: *individual work modifications* or *job coaching*, based on the participants' perception of whether their complaints are work-related or not. This perception is questioned in the baseline screening questionnaire. If the participant perceives their stress-related complaints as work-related, the PA variant: *individual work modifications* will be offered. Conversely, if the issues are perceived to be unrelated to work, the participant will receive the PA variant: *job coaching.* Below, the six steps of PA explained in detail:

- *Step 1 – Preparation:* The process facilitator will contact the participant and their supervisor to inform about the PA and the upcoming steps. Also, meetings for execution of the PA are planned. When the participant has already fully returned to work within 6 weeks, the PA is not started. The process facilitator will then inform the occupational physician (when permission is granted) and ends their role as facilitator.
- *Step 2 – Barriers:* In this step, the most important barriers for returning to work are identified. First, individually between the process facilitator and the participant, and secondly between the process facilitator and the supervisor. This is done in two separate meetings in order to guide the participant and their supervisor in determining the most important and pressing task-related barriers to work. Further, the main responsibilities and activities of the participant are mapped out, indicating how often each task occurs and to what extent it impedes RTW.
- *Step 3 – Solutions:* In a three-way meeting (participant, supervisor and process facilitator) the participant and supervisor discuss the most important barriers for RTW. The aim is to reach consensus on which barriers should be addressed first for the RTW plan, guided by the process

For peer review only - http://bmjopen.bmj.com/site/about/guidelines.xhtml

facilitator. Subsequently, both the participant and the supervisor prioritise solutions for the selected barriers.

- *Step 4 – RTW plan:* The selected barriers and solutions, as identified in steps 1-3, are documented in the RTW plan, including deadlines and responsibilities.
- *Step 5 – Implementation:* In this phase the RTW plan is implemented within a time frame of 3 months.
- *Step 6 – Evaluation:* During the evaluation session, the RTW plan is evaluated. If necessary, the RTW plan will be adjusted.

The difference of the two PA variants lies in the presence of the process facilitator during the meetings. In the individual work modifications variant, the process facilitator will be physically present during all meetings with the participant and supervisor and guides the process. While for the job coaching variant, the process facilitator will remotely coach the process of the PA, and can be consulted by the participant and/or supervisor when needed.

Sample size

The sample size calculation is based on the primary outcome: time to sustainable RTW. Based on previous, similar research, time to sustainable RTW needs at least a hazard ratio (HR) of 2.0 to detect a clinical difference and to be relevant from a societal point of view [24]. An HR of 2.0 indicates that participants in the intervention condition RTW twice as quickly as participants in the control condition. In the power analysis, an alpha of 0.05, power of 80%, and a potential dropout rate of 40% was considered. Hence, based on both the power analysis and comparable research, a sample size of 208 participants is needed for this study [22-24]. A total of 208 participants is regarded as highly feasible, as the four participating organisations have a pool of more than 20,000 employees and a sickness absence rate of 2-5% each.

Randomisation

Participants will be allocated to either the intervention or control group with a randomisation ratio of 1:1. Randomisation will be carried out via Castor EDC, an electronic system that manages data collection and security [32]. The randomisation will be performed after the participants signed the informed consent. Table 1 shows the timeline for enrolment, interventions and assessment procedure of the RCT. This is a single-blinded study, in which the participants are unaware of their allocation to the intervention or control group. Due to the need to assign participants to the PA intervention, it is not possible to blind research team members and the process facilitators. All parties involved will be instructed not to disclose the detailed research design to the participants.

For peer review only - http://bmjopen.bmj.com/site/about/guidelines.xhtml

Table 1 Participant timeline: Schedule of enrolment, interventions, and assessments

	STUDY PERIOD					
	**Enrolment**	**Post-allocation**				**Close-out**
**TIMEPOINT**		***t_0* baseline**	***6 weeks***	***t_1* 3 months**	***t_2* 6 months**	***t_3* 12 months**
**ENROLMENT:**						
**Eligibility screening**	x					
**Informed consent**	x					
**Randomisation**	x					
**INTERVENTIONS:**						
**eHealth modules**		———	———			
**Participatory approach**			———	———		
**Care as usual (all participants)**		———	———	———	———	———
**ASSESSMENTS:**						
**Participant characteristics**		x				
**RTW**	x	x	x	x	x	x
**Stress-related complaints**	x	x		x	x	x
**Self-reported health**		x		x	x	x
**Psychosocial job characteristics**		x		x	x	x
**Self-efficacy for RTW**		x		x	x	x
**Behavioural change for RTW**		x				

For peer review only

Primary outcome

Return to work

The primary outcome is time to sustainable RTW, defined as a return to the employee's previous or a similar position for at least 28 consecutive days without a partial or full relapse. RTW will be assessed through monthly self-reports via the online personal accounts in the eHealth program Werk@Herstel. Participants will receive 12 monthly self-reports during the trial period. The report starts with the question, *'Are you currently completely absent from work?'*, and the participant indicates absence status with a 'yes' or 'no' and notes any work adjustments. Those not fully absent specify the percentage (%)

For peer review only - http://bmjopen.bmj.com/site/about/guidelines.xhtml

of work resumed. Permission will be requested to obtain this data through sick leave register systems from the OHS of the employed organisation after 12 months (end of trial). Additionally, other work-related outcomes will be similarly measured after 12 months, such as RTW in any type of work and number of sick days.

Secondary outcomes

Stress-related complaints

Stress-related complaints will be measured using the 4DSQ self-report questionnaire across the four dimensions distress, depression, anxiety, and somatisation [29]. The questionnaire consists of 50 items, measuring the severity of stress-related symptoms experienced in the past seven days. The 4DSQ is scored on a 5-point Likert scale ranging from ‘no complaints’ to ‘very often or continuously’. The scores are converted to a 3-point scale with (0) ‘no complaints’ to (2) ‘regularly’ and upwards. The 4DSQ has demonstrated both validity and reliability in general practice, mental healthcare, and research settings (Cronbach’s alpha of respective domains: 0.96; 0.96; 0.96; and 0.92) [33, 34] . The 4DSQ will be disseminated at baseline, and after 3, 6 and 12 months. The Patient Reported Outcomes Measurement Information System Questionnaire (PROMIS) will be used to measure the remaining self-reported health outcomes [35]. The PROMIS in this study consists of a short version with 10 questions measuring physical, mental and social well-being across various domains. The questions about experienced well-being in the past seven days are scored on a 5-point Likert scale ranging from (1) ‘poor well-being’ to (5) ‘excellent well-being’. In addition, a slider ranging from (0) 'no pain' to (10) 'worst pain imaginable' can be used to indicate pain experienced in the past week. In a previous study, the PROMIS short form showed excellent internal consistency (Cronbach’s alpha: >0.906) [36]. The PROMIS will be disseminated at baseline, and after 3, 6 and 12 months.

Psychosocial job characteristics

Psychosocial job characteristics will be measured by the Job Content Questionnaire (JCQ), a validated instrument for measuring psychosocial job characteristics [37]. Psychosocial job characteristics refer to the psychological and social aspects of work and the work environment. These include the mental workload, decision latitude, social support, physical demands, and job insecurity. A 4-point Likert scale, ranging from (1) ‘strongly disagree’ to (4) ‘strongly agree’, will be used to assess levels of agreements to certain statements. An example of a statement is: *'I have many opportunities to participate in decisions about what happens in my work'*. The degree of job insecurity in the past year will be asked in direct questions. The scale reliability of the JCQ is dependent of occupation, but overall acceptable (Cronbach’s alpha: 0.61-0.87) [38]. The JCQ will be administered at baseline, and after 3, 6 and 12 months.

For peer review only - http://bmjopen.bmj.com/site/about/guidelines.xhtml

Behaviour change

Behaviour change will be assessed by means of the ASE (Attitude, Social influence and Self-Efficacy) and Return-to-Work Self-Efficacy (RTW-SE) [22, 39]. The ASE is a questionnaire that identifies the three factors influencing behaviour, specifically in the context of returning to work. For this questionnaire a 5-point Likert scale is used, ranging from (1) 'strongly agree' to (5) 'strongly disagree', for statements like *'Despite my complaints, I am going back to work, because that contributes to my recovery'*. The RTW-SE is a similar questionnaire, but focuses on an individual's self-efficacy regarding their confidence in successfully returning to work. The RTW-SE is scored on a 6-point Likert scale ranging from (1) 'strongly disagree' to (6) 'strongly agree'. The RTW-SE has proven to be of excellent internal consistency in a similar setting (Cronbach's alpha: 0.91) [40]. The ASE will be administered only at baseline, whilst the RTW-SE will be administered at baseline, as well after 3, 6 and 12 months.

Covariates

The following covariates will be collected: 1) demographic data, 2) job characteristics, 3) psychosocial risk-factors. Demographic data includes age, gender, nationality, educational level, and family situation. Job characteristics include type of work, type of employment contract (permanent or temporary), and working hours. Both demographic data and job characteristics will be administered at baseline. Psychological risk-factors will be measured by the JCQ, which will be administered at baseline and after 3, 6, and 12 months.

Process evaluation

The process and feasibility of the intervention will be assessed by means of a process evaluation to gain insight in the adoption as well as the barriers and facilitators to implementation of the intervention among the four organisations, based on the RE-AIM and PRISM frameworks [25-27]. The aim of this process evaluation is to study the adoption and implementation of the intervention to gain insight into factors that improve or restrain sustainable implementation among sick-listed workers, supervisors, and process facilitators. Therefore, the following process indicators will be studied: satisfaction, acceptability, reach, adoption, dose delivered and dose received, and penetration and sustainability among different end-users (sick-listed worker, supervisor, and process facilitator). Data will be gathered and analysed both quantitatively and qualitatively. Via logs the process measures reach, adoption, and dose delivered and received will be gathered. To illustrate, the usage of the eHealth program and attendance logs will be tracked. At approximately 9 months after commencement of the study, qualitatively the satisfaction, acceptability and barriers and facilitators to penetration and sustainability of the implementation of the intervention in individual semi-structured interviews with employees (n~10), a focus group interview or individual interview with supervisors (n~6) (depending on participant preference), and a focus group interview among process facilitators (n~6) as the implementers of the

For peer review only - http://bmjopen.bmj.com/site/about/guidelines.xhtml

intervention will be conducted. The interviews will be audio-recorded and transcribed verbatim. Analyses will be performed in SPSS/R for the quantitative measures and MAXQDA software for the qualitative measures.

Statistical analyses

Analyses will be conducted according to the intention-to-treat principle (ITT), meaning that participants will be analysed in the groups to which they were originally assigned. A descriptive analysis will be conducted for baseline characteristics and other variables, such as gender, age, educational level, occupation, and sick leave duration. Longitudinal survival analyses will be conducted to compare the effect of the intervention group with the control group over time, including multiple subgroup analyses. The Cox proportional hazard model will be employed to compute hazard ratios and standard errors will be appropriately adjusted for clustering if needed. Furthermore, confounding and/or effect modification of several factors (e.g., gender, age, education) will be checked. The quantitative data will be analysed using SPSS/R, regarding the qualitative MAXQDA will be used.

Patient and public involvement

Patients and the public were involved at several stages of this study. Prior to the start of the trial, focus groups with employees, supervisors, and management of the participating organisations were conducted. During these focus groups, barriers and insights for implementation and intervention readiness were discussed to shape the current study design [23]. The process evaluation of Volker et al. [23] also found that the occupational physicians, who acted as process facilitators, provided limited support and applied the intervention only to a limited extent [22]. Therefore, in the current study, other occupational health professionals working at the participating organisation have been trained and deployed as dedicated process facilitator.

Ethics and dissemination

The trial has been approved by the Medical Ethics Committee of Amsterdam UMC, which ruled that the Medical Research Involving Human Subjects Act (WMO) does not apply to this study (approval number: 2024.0021). All participants will be informed that participation is voluntary and that they can withdraw from the study at any time. Data collected prior to withdrawal will still be used. Participants will receive both written and verbal information and will have the opportunity to ask questions prior, during and after the study. Access to participant data is restricted to the researchers directly involved in the study. All participants receive an individual login code for the eHealth program. Only the researcher team members and the web administrator can access all individual accounts. At the start of the study, each participant will be assigned a unique study code under which all collected data will be stored.

For peer review only - http://bmjopen.bmj.com/site/about/guidelines.xhtml

Identification keys will be stored in a separate location and secured with a password. The trial is registered at ClinicalTrials.gov on August 8, 2024 (trial registration number: NCT06543732). Recruitment of the study started on 6 January 2025 and the trial is expected to be completed in December 2026.

Results are expected to be published in peer-reviewed journals, and will be presented at national and international conferences. In addition, interim and final results will be shared with the participating organisations involved in this study. During committee meetings with these organisations, also the potential continuation of the intervention within their organisation will be discussed. Participants may receive updates or results of the study upon request.

For peer review only

Discussion

In this paper, the design of an RCT evaluating the effectiveness of the Vital@Work reintegration program for sick-listed workers with stress-related complaints, is described. Participants in the intervention group, who receive a tailored reintegration program, will be compared with a control group. This intervention combines the effective components of an eHealth program and the PA from previous research [22, 24]. By combining and using a tailored approach, we strive to fill the research gaps of effective RTW programs for workers suffering from stress-related complaints. A key strength of this study lies in its tailored study design, which allows to study different subgroups and contextual factors in participants with stress-related complaints. Another strength concerns the follow-up period in our study. Assessments take place at baseline after 3, 6 and 12 months, providing the opportunity to study participants' RTW and stress-related complaints over the long-term. This is relevant as relapse within one year after RTW frequently occurs [41, 42], and thus evaluating the intervention's effectiveness in the long-term is significant. A few limitations should also be considered. One of the limitations is that the researchers and the process facilitators cannot be blinded due to intervention allocation. However, all involved parties will be instructed not to disclose the research design to the participants, ensuring that the participants remain blinded as the control group is provided with general information via the eHealth program. Another limitation is that the outcome measures are based on self-reported questionnaires (4DSQ, PROMIS, JCQ, ASE, and RTW-SE). Participants may experience recall problems with the monthly reports and a response bias may occur. Hence, to limit potential recall bias for the primary outcome (time to sustainable return to work), sick leave data from the register systems of the OHS of the employed organisation will be obtained (when permission is granted). By using both self-reported and register data, validity could be increased [43].

Authors' contributions

Guarantor is Johannes R. Anema. All authors will participate in the collection and execution of the trial; EEMvD and TTJ are primary executers. Authors AWdS, RMCS, ATFB, JRA and TTJ were responsible

For peer review only - http://bmjopen.bmj.com/site/about/guidelines.xhtml

for the development of the trial intervention, with AWdS writing the non-WMO declaration. Authors RMCS, ATFB, and JRA obtained funding for this study. Author EEMvD wrote the first version of the paper; supervised by TTJ. Author TTJ provided content on the process evaluation. All authors have reviewed and approved the final manuscript.

Funding

This work was supported by Health~Holland, reference number LSHM22035-H025. The Vital@Work project is part of a PPP Allowance, made available by Health~Holland, Top Sector Life Sciences & Health, to stimulate public-private partnerships (https://www.health-holland.com). All involved organisations in the PPP (Amsterdam UMC, GGZ inGeest, Transavia, Rijksschoonmaakorganisatie) contributed in kind. Rijksschoonmaakorganisatie also contributed in cash.

Competing interests

JRA is consultant and certificate-holder of a spin-out company for implementation of the mobile application concerning the IkHerstel intervention in the Netherlands. JRA holds a chair in insurance medicine paid by the Dutch Social Security Agency; and has received grants from ZonMw, Nederlandse Organisatie voor Wetenschappelijk Onderzoek, Instituut Gak, Uitvoeringsinstituut Werknemersverzekeringen, Sociale Zaken en Werkgelegenheid, VWS (Volksgezondheid, Welzijn en Sport), Pfizer, Achmea, CVZ (College voor Zorgverzekeringen), and Zorginstituut; all outside the submitted work. The other authors have no competing interests.

References

1. WHO. World mental health today: latest data. World Health Organization; 2025. Report No.: 9240113819.

2. WHO. World mental health report: Transforming mental health for all. World Health Organization; 2022. Report No.: 9240049339.

3. Niedhammer I, Bertrais S, Witt K. Psychosocial work exposures and health outcomes: a meta-review of 72 literature reviews with meta-analysis. Scandinavian journal of work, environment & health. 2021;47(7):489.

4. de Oliveira C, Saka M, Bone L, Jacobs R. The role of mental health on workplace productivity: a critical review of the literature. Applied health economics and health policy. 2023;21(2):167–93.

5. Wolvetang S, van Dongen JM, Speklé E, Coenen P, Schaafsma F. Sick leave due to stress, what are the costs for Dutch employers? Journal of Occupational Rehabilitation. 2022;32(4):764–72.

6. VZinfo. Ziekteverzuim | Naar oorzaak 2025 [Available from: https://www.vzinfo.nl/ziekteverzuim/naar-oorzaak.

7. Hassard J, Teoh KRH, Visockaite G, Dewe P, Cox T. The cost of work-related stress to society: A systematic review. Journal of occupational health psychology. 2018;23(1):1.

For peer review only - http://bmjopen.bmj.com/site/about/guidelines.xhtml

8. Chisholm D, Sweeny K, Sheehan P, Rasmussen B, Smit F, Cuijpers P, et al. Scaling-up treatment of depression and anxiety: a global return on investment analysis. The Lancet Psychiatry. 2016;3(5):415–24.

9. Van den Heuvel SG, Fernandez Beiro L, Van Dam LMC. Nationale enquête arbeidsomstandigheden 2021: Resultaten in vogelvlucht. TNO & CBS; 2022.

10. Goh J, Pfeffer J, Zenios SA. The relationship between workplace stressors and mortality and health costs in the United States. Management Science. 2016;62(2):608–28.

11. Vogel N, Schandelmaier S, Zumbrunn T, Ebrahim S, de Boer WEL, Busse JW, et al. Return-to-work coordination programmes for improving return to work in workers on sick leave. Cochrane Database of Systematic Reviews. 2017(3).

12. Prinz C. Sickness, disability and work: lessons from reforms and lack of change across the OECD countries. 2010.

13. Mikkelsen MB, Rosholm M. Systematic review and meta-analysis of interventions aimed at enhancing return to work for sick-listed workers with common mental disorders, stress-related disorders, somatoform disorders and personality disorders. Occupational and environmental medicine. 2018;75(9):675–86.

14. Tingulstad A, Meneses-Echavez J, Evensen LH, Bjerk M, Berg RC. Effectiveness of work-related interventions for return to work in people on sick leave: a systematic review and meta-analysis of randomized controlled trials. Systematic Reviews. 2022;11(1):192.

15. Blonk RWB, Brenninkmeijer V, Lagerveld SE, Houtman ILD. Rehabilitation in case of work-related psychological complaints: A field experiment among the self-employed. Work and Stress. 2006;20(2):129–44.

16. Dalgaard VL, Aschbacher K, Andersen JH, Glasscock DJ, Willert MV, Carstensen O, et al. Return to work after work-related stress: a randomized controlled trial of a work-focused cognitive behavioral intervention. Scandinavian Journal of Work, Environment & Health. 2017:436–46.

17. Hedlund Å, Kristofferzon M-L, Boman E, Nilsson A. Are return to work beliefs, psychological well-being and perceived health related to return-to-work intentions among women on long-term sick leave for common mental disorders? A cross-sectional study based on the theory of planned behaviour. BMC public health. 2021;21(1):535.

18. Buys NJ, Selander J, Sun J. Employee experience of workplace supervisor contact and support during long-term sickness absence. Disability and Rehabilitation. 2019;41(7):808–14.

19. Netterstrøm B, Friebel L, Ladegaard Y. Effects of a multidisciplinary stress treatment programme on patient return to work rate and symptom reduction: results from a randomised, wait-list controlled trial. Psychotherapy and psychosomatics. 2013;82(3):177–86.

20. Martin MHT, Nielsen MBD, Madsen IEH, Petersen SMA, Lange T, Rugulies R. Effectiveness of a coordinated and tailored return-to-work intervention for sickness absence beneficiaries with mental health problems. Journal of occupational rehabilitation. 2013;23(4):621–30.

21. Bültmann U, Sherson D, Olsen J, Hansen CL, Lund T, Kilsgaard J. Coordinated and tailored work rehabilitation: a randomized controlled trial with economic evaluation undertaken with workers on sick leave due to musculoskeletal disorders. Journal of occupational rehabilitation. 2009;19(1):81–93.

For peer review only - http://bmjopen.bmj.com/site/about/guidelines.xhtml

22. Volker D, Zijlstra-Vlasveld MC, Anema JR, Beekman ATF, Brouwers EPM, Emons WHM, et al. Effectiveness of a blended web-based intervention on return to work for sick-listed employees with common mental disorders: results of a cluster randomized controlled trial. Journal of medical Internet research. 2015;17(5):e116.

23. Volker D, Zijlstra-Vlasveld MC, Brouwers EPM, Van der Feltz-Cornelis CM. Process evaluation of a blended web-based intervention on return to work for sick-listed employees with common mental health problems in the occupational health setting. Journal of Occupational Rehabilitation. 2017;27(2):186–94.

24. van Oostrom SH, van Mechelen W, Terluin B, de Vet HCW, Knol DL, Anema JR. A workplace intervention for sick-listed employees with distress: results of a randomised controlled trial. Occupational and environmental medicine. 2010;67(9):596–602.

25. Gaglio B, Shoup JA, Glasgow RE. The RE-AIM framework: a systematic review of use over time. American journal of public health. 2013;103(6):e38–e46.

26. Feldstein AC, Glasgow RE. A practical, robust implementation and sustainability model (PRISM) for integrating research findings into practice. The joint commission journal on quality and patient safety. 2008;34(4):228–43.

27. Glasgow RE, Vogt TM, Boles SM. Evaluating the public health impact of health promotion interventions: the RE-AIM framework. American journal of public health. 1999;89(9):1322–7.

28. Chan A-W, Boutron I, Hopewell S, Moher D, Schulz KF, Collins GS, et al. SPIRIT 2025 statement: updated guideline for protocols of randomised trials. The Lancet. 2025;405(10491):e19–e27.

29. Terluin B, van Marwijk HWJ, Adèr HJ, de Vet HCW, Penninx BWJH, Hermens MLM, et al. The Four-Dimensional Symptom Questionnaire (4DSQ): a validation study of a multidimensional self-report questionnaire to assess distress, depression, anxiety and somatization. BMC psychiatry. 2006;6(1):34.

30. NVAB. Richtlijn Psychische problemen. 2019.

31. Terluin B, Oosterbaan DB, Brouwers EP, Van Straten A, Van de Ven PM, Langerak W, et al. To what extent does the anxiety scale of the Four-Dimensional Symptom Questionnaire (4DSQ) detect specific types of anxiety disorder in primary care? A psychometric study. BMC psychiatry. 2014;14(1):121.

32. CastorEDC. 2024 [Available from: https://www.castoredc.com/].

33. Kleinstäuber M, Exner A, Lambert MJ, Terluin B. Validation of the Four-Dimensional Symptom Questionnaire (4DSQ) in a mental health setting. Psychology, health & medicine. 2021;26(sup1):1–19.

34. Terluin B, Verbraak M. Validiteit van de Vierdimensionale Klachtenlijst (4DKL) in de ambulante geestelijke gezondheidszorg. Tijdschrift voor klinische psychologie. 2014;44(3):198–211.

35. Terwee CB, Roorda LD, De Vet HCW, Dekker J, Westhovens R, Van Leeuwen J, et al. Dutch–Flemish translation of 17 item banks from the patient-reported outcomes measurement information system (PROMIS). Quality of Life Research. 2014;23(6):1733–41.

36. Bartlett SJ, Orbai A-M, Duncan T, DeLeon E, Ruffing V, Clegg-Smith K, et al. Reliability and validity of selected PROMIS measures in people with rheumatoid arthritis. PloS one. 2015;10(9):e0138543.

For peer review only - http://bmjopen.bmj.com/site/about/guidelines.xhtml

37. Karasek R, Brisson C, Kawakami N, Houtman I, Bongers P, Amick B. The Job Content Questionnaire (JCQ): an instrument for internationally comparative assessments of psychosocial job characteristics. Journal of occupational health psychology. 1998;3(4):322.

38. Choi B, Kurowski A, Bond M, Baker D, Clays E, De Bacquer D, et al. Occupation-differential construct validity of the Job Content Questionnaire (JCQ) psychological job demands scale with physical job demands items: a mixed methods research. Ergonomics. 2012;55(4):425–39.

39. de Rijk A, Janssen N, van Lierop B, Alexanderson K, Nijhuis F. A behavioral approach to RTW after sickness absence: the development of instruments for the assessment of motivational determinants, motivation and key actors' attitudes. Work. 2009;33(3):273–85.

40. Gjengedal RGH, Lagerveld SE, Reme SE, Osnes K, Sandin K, Hjemdal O. The return-to-work self-efficacy questionnaire (RTW-SE): a validation study of predictive abilities and cut-off values for patients on sick leave due to anxiety or depression. Journal of occupational rehabilitation. 2021;31(3):664–73.

41. Koopmans PC, Bültmann U, Roelen CAM, Hoedeman R, van der Klink JJL, Groothoff JW. Recurrence of sickness absence due to common mental disorders. International archives of occupational and environmental health. 2011;84(2):193–201.

42. Spronken M, Brouwers EPM, Vermunt JK, Arends I, Oerlemans WGM, Van Der Klink JJL, et al. Identifying return to work trajectories among employees on sick leave due to mental health problems using latent class transition analysis. BMJ open. 2020;10(2):e032016.

43. Lavreysen O, Bakusic J, Abatzi T-A, Geerts A, Mateusen M, Bashkin O, et al. An overview of work-related stress assessment. Journal of affective disorders. 2025.

Figure legends

Fig. 1 Decision aid of tailored eHealth pathways intervention group

Figure 1 displays a simplified representation of eHealth pathways. The pathways are determined on participants' randomisation, followed by the two navigating questions and 4DSQ. Cut-off scores are used to determine the tailored pathways [29, 31]. The figure was created by EEMvD using Lucidchart.

For peer review only

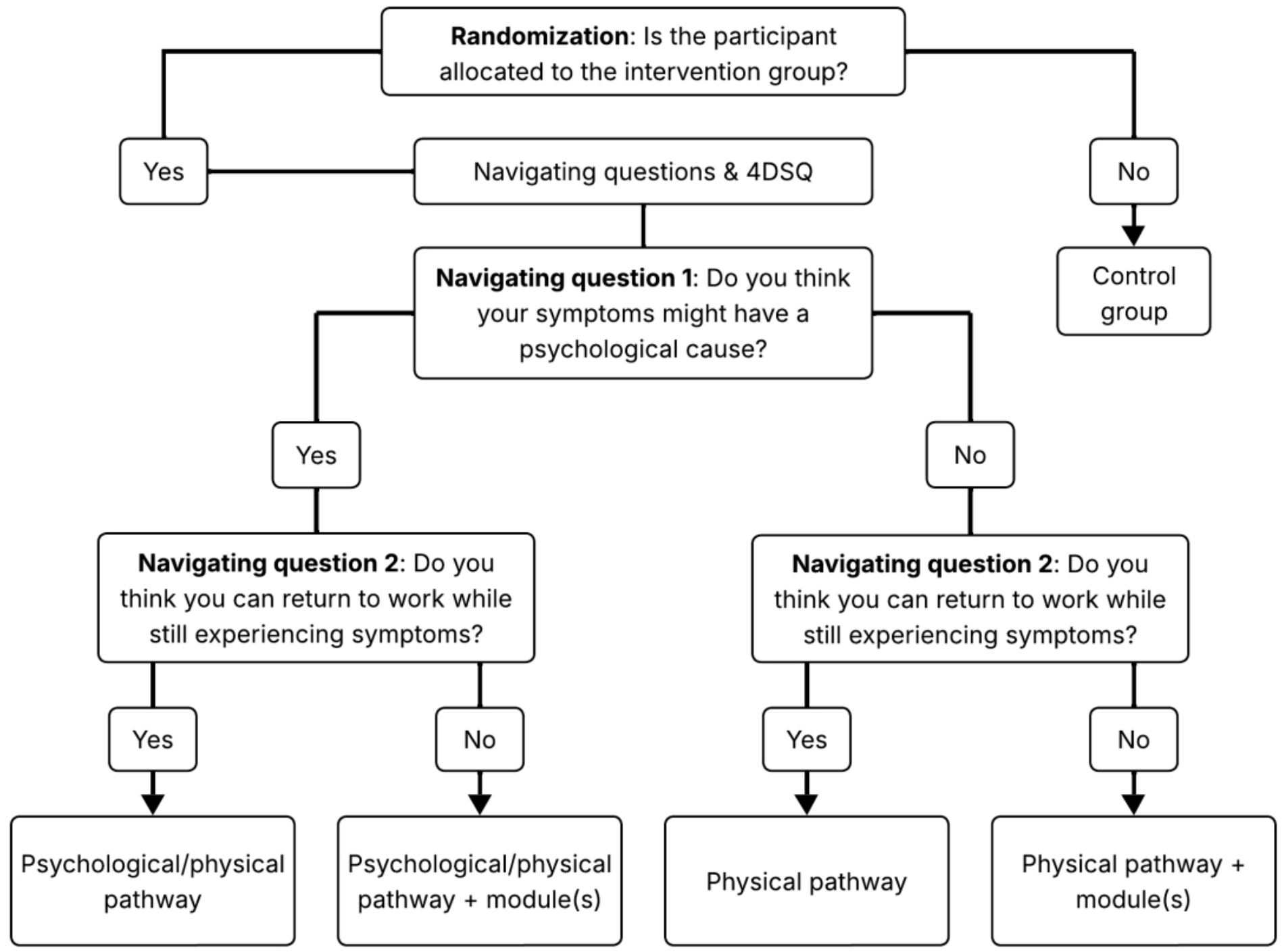

Fig. 1 Decision aid of tailored eHealth pathways intervention group

500x379mm (118 x 118 DPI)

SPIRIT 2025 checklist of items to address in a randomized trial protocol*

Section / Topic	No	SPIRIT 2025 checklist item description	Reported on page no.
**Administrative information**			
Title and structured summary	1a	Title stating the trial design, population, and interventions, with identification as a protocol	1
	1b	Structured summary of trial design and methods, including items from the World Health Organization Trial Registration Data Set	1-2
Protocol version	2	Version date and identifier	2
Roles and responsibilities	3a	Names, affiliations, and roles of protocol contributors	1 and 14
	3b	Name and contact information for the trial sponsor	14
	3c	Role of trial sponsor and funders in design, conduct, analysis, and reporting of trial; including any authority over these activities	14
	3d	Composition, roles, and responsibilities of the coordinating site, steering committee, endpoint adjudication committee, data management team, and other individuals or groups overseeing the trial, if applicable	14
**Open science**			
Trial registration	4	Name of trial registry, identifying number (with URL), and date of registration. If not yet registered, name of intended registry	2 and 13
Protocol and statistical analysis plan	5	Where the trial protocol and statistical analysis plan can be accessed	13
Data sharing	6	Where and how the individual de-identified participant data (including data dictionary), statistical code, and any other materials will be accessible	13
Funding and conflicts of interest	7a	Sources of funding and other support (e.g., supply of drugs)	14
	7b	Financial and other conflicts of interest for principal investigators and steering committee members	14
Dissemination policy	8	Plans to communicate trial results to participants, healthcare professionals, the public, and other relevant groups (e.g., reporting in trial registry, plain language summary, publication)	12-13
**Introduction**			
Background and rationale	9a	Scientific background and rationale, including summary of relevant studies (published and unpublished) examining benefits and harms for each intervention	2-4
	9b	Explanation for choice of comparator	2-4
Objectives	10	Specific objectives related to benefits and harms	12-13
**Methods: Patient and public involvement, trial design**			
Patient and public involvement	11	Details of, or plans for, patient or public involvement in the design, conduct, and reporting of the trial	12-13
Trial design	12	Description of trial design including type of trial (e.g., parallel group, crossover), allocation ratio, and framework (e.g., superiority, equivalence, non-inferiority, exploratory)	4

For peer review only - http://bmjopen.bmj.com/site/about/guidelines.xhtml

**Methods: Participants, interventions, and outcomes**			
Trial setting	13	Settings (e.g., community, hospital) and locations (e.g., countries, sites) where the trial will be conducted	4
Eligibility criteria	14a	Eligibility criteria for participants	4
	14b	If applicable, eligibility criteria for sites and for individuals who will deliver the interventions (e.g., surgeons, physiotherapists)	5-6
Intervention and comparator	15a	Intervention and comparator with sufficient details to allow replication including how, when, and by whom they will be administered. If relevant, where additional materials describing the intervention and comparator (e.g., intervention manual) can be accessed	5-8
	15b	Criteria for discontinuing or modifying allocated intervention/comparator for a trial participant (e.g., drug dose change in response to harms, participant request, or improving/worsening disease)	n/a
	15c	Strategies to improve adherence to intervention/comparator protocols, if applicable, and any procedures for monitoring adherence (e.g., drug tablet return, sessions attended)	5-7
	15d	Concomitant care that is permitted or prohibited during the trial	n/a
Outcomes	16	Primary and secondary outcomes, including the specific measurement variable (e.g., systolic blood pressure), analysis metric (e.g., change from baseline, final value, time to event), method of aggregation (e.g., median, proportion), and time point for each outcome	9-11
Harms	17	How harms are defined and will be assessed (e.g., systematically, non-systematically)	n/a
Participant timeline	18	Time schedule of enrollment, interventions (including any run-ins and washouts), assessments, and visits for participants. A schematic diagram is highly recommended (see Figure)	9 (table 1)
Sample size	19	How sample size was determined, including all assumptions supporting the sample size calculation	8
Recruitment	20	Strategies for achieving adequate participant enrollment to reach target sample size	5-6 and 8
**Methods: Assignment of interventions**			
Randomization:			
Sequence generation	21a	Who will generate the random allocation sequence and the method used	8
	21b	Type of randomization (simple or restricted) and details of any factors for stratification. To reduce predictability of a random sequence, other details of any planned restriction (e.g., blocking) should be provided in a separate document that is unavailable to those who enroll participants or assign interventions	8
Allocation concealment mechanism	22	Mechanism used to implement the random allocation sequence (e.g., central computer/telephone; sequentially numbered, opaque, sealed containers), describing any steps to conceal the sequence until interventions are assigned	8
Implementation	23	Whether the personnel who will enroll and those who will assign participants to the interventions will have access to the random allocation sequence	8
Blinding	24a	Who will be blinded after assignment to interventions (e.g., participants, care providers, outcome assessors, data analysts)	5 and 8

For peer review only - http://bmjopen.bmj.com/site/about/guidelines.xhtml

	24b	If blinded, how blinding will be achieved and description of the similarity of interventions	5 and 8
	24c	If blinded, circumstances under which unblinding is permissible, and procedure for revealing a participant's allocated intervention during the trial	n/a
**Methods: Data collection, management, and analysis**			
Data collection methods	25a	Plans for assessment and collection of trial data, including any related processes to promote data quality (e.g., duplicate measurements, training of assessors) and a description of trial instruments (e.g., questionnaires, laboratory tests) along with their reliability and validity, if known. Reference to where data collection forms can be accessed, if not in the protocol	9-12
	25b	Plans to promote participant retention and complete follow-up, including list of any outcome data to be collected for participants who discontinue or deviate from intervention protocols	n/a
Data management	26	Plans for data entry, coding, security, and storage, including any related processes to promote data quality (e.g., double data entry; range checks for data values). Reference to where details of data management procedures can be accessed, if not in the protocol	12-13
Statistical methods	27a	Statistical methods used to compare groups for primary and secondary outcomes, including harms	12
	27b	Definition of who will be included in each analysis (e.g., all randomized participants), and in which group	12
	27c	How missing data will be handled in the analysis	12
	27d	Methods for any additional analyses (e.g., subgroup and sensitivity analyses)	12
**Methods: Monitoring**			
Data monitoring committee	28a	Composition of data monitoring committee (DMC); summary of its role and reporting structure; statement of whether it is independent from the sponsor and funder; conflicts of interest and reference to where further details about its charter can be found, if not in the protocol. Alternatively, an explanation of why a DMC is not needed	14
	28b	Explanation of any interim analyses and stopping guidelines, including who will have access to these interim results and make the final decision to terminate the trial	12-13
Trial monitoring	29	Frequency and procedures for monitoring trial conduct. If there is no monitoring, give explanation	6-7
**Ethics**			
Research ethics approval	30	Plans for seeking research ethics committee/institutional review board approval	12-13
Protocol amendments	31	Plans for communicating important protocol modifications to relevant parties	n/a
Consent or assent	32a	Who will obtain informed consent or assent from potential trial participants or authorized proxies, and how	5
	32b	Additional consent provisions for collection and use of participant data and biological specimens in ancillary studies, if applicable	5
Confidentiality	33	How personal information about potential and enrolled participants will be collected, shared, and maintained in order to protect confidentiality before, during, and after the trial	12-13
Ancillary and post-trial care	34	Provisions, if any, for ancillary and post-trial care, and for compensation to those who suffer harm from trial participation	n/a

For peer review only - http://bmjopen.bmj.com/site/about/guidelines.xhtml

*We strongly recommend reading this checklist in conjunction with the SPIRIT 2025 Explanation and Elaboration and the SPIRIT 2025 Expanded Checklist for important clarifications on all the items. We also recommend reading relevant SPIRIT extensions. See www.consort-spirit.org

Citation: Chan A-W, Boutron I, Hopewell S, Moher D, Schulz KF, et al. SPIRIT 2025 statement: updated guideline for protocols of randomised trials. BMJ 2025;389:e081477. https://dx.doi.org/10.1136/bmj-2024-081477

© 2025 Chan A-W et al. This is an Open Access article distributed under the terms of the Creative Commons Attribution License (https://creativecommons.org/licenses/by/4.0/), which permits unrestricted use, distribution, and reproduction in any medium, provided the original work is properly cited.

For peer review only

For peer review only - http://bmjopen.bmj.com/site/about/guidelines.xhtml

Informed consent form (participant)

Vital@Work: a re-integration programme for employees with stress-related complaints

- I have read the information letter. I was also able to ask questions. My questions have been sufficiently answered. I had enough time to decide whether I want to participate.
- I understand that participation is voluntary. I also understand that I can withdraw from the study at any time. I do not have to provide a reason for this.
- I give permission for my data to be collected and used in the manner and for the purposes as described in the information letter.
- I give permission for my data to be stored within Amsterdam UMC for another 10 years after this study.
- I would like to participate in this study.

I give permission for the collection of my data:

O yes
O no

Date of permission (dd-mm-yyyy):

____

I give permission for requesting my (partial) recovery notifications from the absence database at my occupational health service:

O yes
O no

I give permission to inform my occupational physician about my participation in the study:

O yes
O no

I give permission to be contacted to take part in an interview:

O yes
O no

I give permission to be contacted again after this study for follow-up research:

O yes
O no

For peer review only - http://bmjopen.bmj.com/site/about/guidelines.xhtml
