# Supplementary material for: Vital@Work reintegration programme for sick-listed workers with stress-related complaints in occupational healthcare in the Netherlands: study protocol of a randomised controlled trial
Source: BMJ Open. 2026 Sep 30;16(9):e116576. doi: 10.1136/bmjopen-2026-116576 (PMC13636312; doi:10.1136/bmjopen-2026-116576)
Supplement: Supplementary file 4 [file bmjopen-2026-116576.reviewer_comments.pdf]

PEER REVIEW HISTORY

BMJ Open publishes all reviews undertaken for accepted manuscripts. Reviewers are asked to complete a checklist review form and are provided with free text boxes to elaborate on their assessment. These free text comments are reproduced below.

ARTICLE DETAILS

Title (Provisional)

Vital@Work reintegration program for sick-listed workers with stress-related complaints in Occupational Healthcare in the Netherlands: study protocol of a randomised controlled trial

Authors

van Doeveren, Eva E. M.; de Smalen, Allard W; Schelvis, Roosmarijn M. C.; Beekman, Aartjan T. F.; Anema, Johannes R.; Juurlink, Trees T

VERSION 1 - REVIEW

**Reviewer**	**1**
**Name**	**Goulden, Nia**
**Affiliation**	**Bangor University, NWORTH CTU**
**Date**	**27-Mar-2026**
**COI**	**None**

This manuscript describes a protocol to describe a randomised controlled trial of a work reintegration program for those who have been on sick leave due to stress. The manuscript is well written and thorough and present an interesting trial.

**Reviewer**	**2**
**Name**	**Chaudhury, Suprakash**
**Affiliation**	**Pravara Institute of Medical Sciences (Deemed University) Rural Medical College and Pravara's Rural Hospital, Department of Psychiatry,**
**Date**	**07-Jul-2026**
**COI**	**None**

A well planned study.

However, the exclusion criteria include: Exclusion criteria are:

1) suffering from severe psychiatric disorders (e.g. suicidality, schizophrenia) or other chronic

conditions (e.g. terminal illness) that prevent RTW, 2) insufficient Dutch language skills to complete

eHealth modules and/or questionnaires, 3) in legal conflict with the employer, 4) pregnancy

It is not clear if subjects with substance use disorders, depression, dysthymia, adjustment disorders, PTSD etc will be included. Since they will receive CAU, that means the study group will have one group that receives medications + intervention and another group that will receive only medications.

Presumably, the control group will similarly have 2 groups. Will these groups be homogenous and comparable ?

VERSION 1 - AUTHOR RESPONSE

Reviewer 1

This manuscript describes a protocol to describe a randomised controlled trial of a work reintegration program for those who have been on sick leave due to stress. The manuscript is well written and thorough and present an interesting trial.

We thank the reviewer for their encouraging comment, and are pleased that the manuscript was considered to be well written and the trial of interest.

Reviewer 2

A well planned study.

However, the exclusion criteria include: Exclusion criteria are:

1) suffering from severe psychiatric disorders (e.g. suicidality, schizophrenia) or other chronic conditions (e.g. terminal illness) that prevent RTW, 2) insufficient Dutch language skills to complete eHealth modules and/or questionnaires, 3) in legal conflict with the employer, 4) pregnancy

It is not clear if subjects with substance use disorders, depression, dysthymia, adjustment disorders, PTSD etc will be included. Since they will receive CAU, that means the study group will have one group that receives medications + intervention and another group that will receive only medications. Presumably, the control group will similarly have 2 groups. Will these groups be homogenous and comparable ?

We thank the reviewer for their comment. We would like to clarify that individuals will be excluded if they have a severe psychiatric disorder or a chronic condition that is expected to impede their return to work. This can include, for example, chronic conditions or psychiatric disorders requiring treatment, such as substance use disorder, severe depression, or PTSD, however only if the condition

itself or its treatment is anticipated to substantially interfere with the return to work process. For clarification we have amended the section enumerating the exclusion criteria (p. 4): "Exclusion criteria are: 1) suffering from severe psychiatric disorders (e.g. suicidality, schizophrenia) or other chronic conditions (e.g. terminal illness) that substantially intervenes with RTW,…."
Furthermore, care as usual (CAU) in this study refers to the standard care provided by the occupational health service. In the Dutch context, under the Dutch Gatekeeper Improvement Act, employees are entitled to occupational health care, whereby an occupational physician conducts a problem analysis and provides guidance based on the employee's medical condition, to facilitate (eventual) return to work. In general, occupational physicians do not prescribe medications. We have further described this in the methods section, under heading 'Intervention' (p. 5): "In the Dutch context, under the Dutch Gatekeeper Improvement Act, CAU consists of employees' entitlement to occupational healthcare, whereby an occupational physician conducts a problem analysis and provides guidance based on the employee's medical condition, to facilitate (eventual) return to work. Participants assigned to the intervention group will, in addition to CAU by the occupational healthcare provider and eventual other care or treatment they receive for their condition, receive the Vital@Work reintegration program, consisting of a tailored eHealth program (see below under
eHealth program) and followed by the PA (see below under Participatory approach) if full RTW has not been achieved after 6 weeks."
The participants will be randomised into two groups: the intervention group (receiving CAU + intervention) or the control group (only CAU).

VERSION 2 - REVIEW

**Reviewer**	**2**
**Name**	**Chaudhury, Suprakash**
**Affiliation**	**Pravara Institute of Medical Sciences (Deemed University) Rural Medical College and Pravara's Rural Hospital, Department of Psychiatry,**
**Date**	**16-Aug-2026**
**COI**	

This manuscript describes a protocol to describe a randomised controlled trial of a work reintegration

program for those who have been on sick leave due to stress. The manuscript is well written and

thorough and present an interesting trial.

VERSION 2 - AUTHOR RESPONSE

Reviewer 2

This manuscript describes a protocol to describe a randomised controlled trial of a work reintegration program for those who have been on sick leave due to stress. The manuscript is well written and thorough and present an interesting trial.

We thank the reviewer for their comment, and are pleased that the clarification of our exclusion criteria in the previous revision has been adequately addressed.
